# A human single-cell atlas identifies OLR1^+^ scar-associated macrophages as a potential therapeutic target for chronic liver disease

**DOI:** 10.1038/s41588-026-02774-w

**Published:** 2026-09-28

**Authors:** Eleni Papachristoforou, Kexin Kong, Fabio Colella, Jacky Tam, Juliet Luft, Ravi Parhar, Yingxin Liang, Ailish McCafferty-Brown, George Finney, Keshav Rao, Jayamary Divya Ravichandar, Elena F. Sutherland, Stefan Veizades, Malgorzata Grzelka, Pak Kwan Qiu, Ayma Asif, Ines Battle, Max Hammer, Theresa Kuschnereit, Raktaprabal Kashyap, Sebastian J. Wallace, John R. Wilson-Kanamori, Ginerva Pistocchi, Tabitha Turner-Stokes, Alvile Kasarinaite, Josepmaria Argemi, Elisa Pose, Ramon Bataller, David Lopez, Anna Zagorska, Grant Budas, Gareth-Rhys Jones, Calum C. Bain, Catalina A. Vallejos, Ashis Mukhopadhya, Neil C. Henderson, David C. Hay, James C. Lee, Laura J. Pallett, Volker M. Lauschke, Timothy J. Kendall, Jonathan A. Fallowfield, Prakash Ramachandran

**Affiliations:** 1 Centre for Inflammation Research, Institute for Regeneration and Repair, University of Edinburgh, Edinburgh, UK; 2 Department of Physiology and Pharmacology, Center for Molecular Medicine, Karolinska Institutet and University Hospital, Stockholm, Sweden; 3 College of Pharmaceutical Sciences, Zhejiang University, Hangzhou, China; 4 Institute of Infection, Immunity and Transplantation, University College London, London, UK; 5 Gilead Sciences, Foster City, CA USA; 6 Centre for Cardiovascular Science, University of Edinburgh, Edinburgh, UK; 7 Genetic Mechanisms of Disease Laboratory, The Francis Crick Institute, London, UK; 8 Department of Immunology and Inflammation, Imperial College London, London, UK; 9 Centre for Regenerative Medicine, Institute for Regeneration and Repair, University of Edinburgh, Edinburgh, UK; 10 CIBERehd, Madrid, Spain; 11 Instituto de Investigaciones Sanitarias de Navarra IdiSNA, Pamplona, Spain; 12 RNA Biology and Therapeutics Program, CIMA, CCUN, University of Navarra, Pamplona, Spain; 13 Institut d’Investigacions Biomediques August Pi i Sunyer (IDIBAPS), Hospital Clinic de Barcelona, Barcelona, Spain; 14 Institute of Genetics and Cancer, University of Edinburgh, Edinburgh, UK; 15 Digestive Disorders Department, Aberdeen Royal Infirmary, Aberdeen, UK; 16 MRC Human Genetics Unit, Institute of Genetics and Cancer, University of Edinburgh, Edinburgh, UK; 17 Department of Gastroenterology, Royal Free Hospital, London, UK; 18 Institute for Liver and Digestive Health, Division of Medicine, University College London, London, UK; 19 Dr. Margarete Fischer-Bosch Institute of Clinical Pharmacology, Stuttgart, Germany; 20 University of Tübingen, Tubingen, Germany; 21 Department of Pharmacy, The Second Xiangya Hospital, Central South University, Changsha, China; 22 Edinburgh Pathology, University of Edinburgh, Edinburgh, UK

**Keywords:** Liver fibrosis, Inflammatory diseases, Computational biology and bioinformatics, Immunology, Transcriptomics

## Abstract

Chronic liver disease (CLD) results in liver fibrosis, leading to adverse clinical outcomes. Macrophages regulate fibrosis and represent an attractive therapeutic target. However, the specific macrophage subpopulation that drives fibrosis in CLD remains unclear. Here we generate a liver single-cell atlas comprising 649,295 cells from 42 healthy and 35 CLD patients, identifying 125 transcriptionally distinct cellular states. We resolve two types of disease-expanded TREM2^+^ scar-associated macrophages (SAMac), including a pro-inflammatory subpopulation that expresses the scavenger receptor OLR1 and spatially accumulates in the CLD fibrotic niche. High hepatic OLR1 expression was associated with increased fibrosis across multiple CLD etiologies and adverse patient outcomes in metabolic dysfunction-associated steatotic liver disease. Corollary pro-inflammatory OLR1^+^ SAMac expanded in mouse CLD, while targeting of macrophage OLR1 attenuated fibrogenic activity in human coculture and multilineage spheroid models. Overall, our atlas provides a reference to study disease-associated cell states in CLD and identifies OLR1 as a potential therapeutic target to attenuate liver fibrosis.

## Abstract (web summary)

This study provides a single-cell RNA-sequencing atlas of 77 healthy and chronic liver disease livers and identifies a subpopulation of pro-inflammatory scar-associated macrophages enriched for OLR1 expression with therapeutic potential.

## Main

Chronic liver disease (CLD) is a major global healthcare problem resulting in approximately 2 million deaths per year^1^. Irrespective of cause, chronic liver damage causes progressive scarring (fibrosis) that can ultimately advance to cirrhosis, leading to clinical complications. Higher levels of liver fibrosis are associated with poor clinical outcomes^2,3^, whereas improvements in fibrosis correlate with a better prognosis^4^. Due to this close relationship between fibrosis and outcomes, there is significant interest in developing new antifibrotic therapies for CLD patients^5,6^

Single-cell RNA sequencing (scRNA-seq) is transforming our understanding of human CLD pathogenesis and the identification of therapeutic targets^5,6^. However, most existing human liver scRNA-seq studies include relatively small cell numbers from limited patient cohorts, constraining their ability to resolve subtle transcriptional states relevant to CLD pathogenesis. Large-scale single-cell atlases have started to address this issue in other human organs^7–9^, but a comprehensive human liver scRNA-seq atlas spanning healthy and diseased states remains unavailable.

Immune cell accumulation is a feature of human CLD and regulates tissue injury responses. Specifically, cells of the mononuclear phagocyte (MP) lineage have been shown to have a crucial role in regulating liver fibrosis^10–12^. Previous human scRNA-seq studies identified disease-expanded TREM2^+^ CD9^+^ scar-associated macrophages (SAMacs) as drivers of scar-producing myofibroblast activation in the liver^13,14^, making them a potential antifibrotic target. However, hepatic macrophages also regulate fibrosis regression and tissue repair^15–18^, with SAMac-expressed molecules such as TREM2 and SPP1 that actually show anti-inflammatory and antifibrotic properties^19–22^. It remains unclear whether distinct SAMac subpopulations mediate divergent functions and how pro-inflammatory, pro-fibrotic SAMacs might be selectively targeted, without compromising reparative macrophage activity.

To address this, we generate a human liver scRNA-seq atlas from healthy and CLD livers. We reveal previously unrecognized SAMac heterogeneity, identifying a distinct subpopulation of OLR1^+^ SAMacs that expand in CLD and show pro-inflammatory properties. High hepatic expression of OLR1 is predictive of adverse clinical outcomes in patients with CLD, while selective targeting of OLR1 reduces fibrogenic activity in human in vitro models. Our work provides a foundational resource to study disease-associated cell states in CLD and highlights OLR1 inhibition as a potential antifibrotic therapeutic target.

## Results

### Establishing a human CLD single-cell atlas

**Fig. 1:**
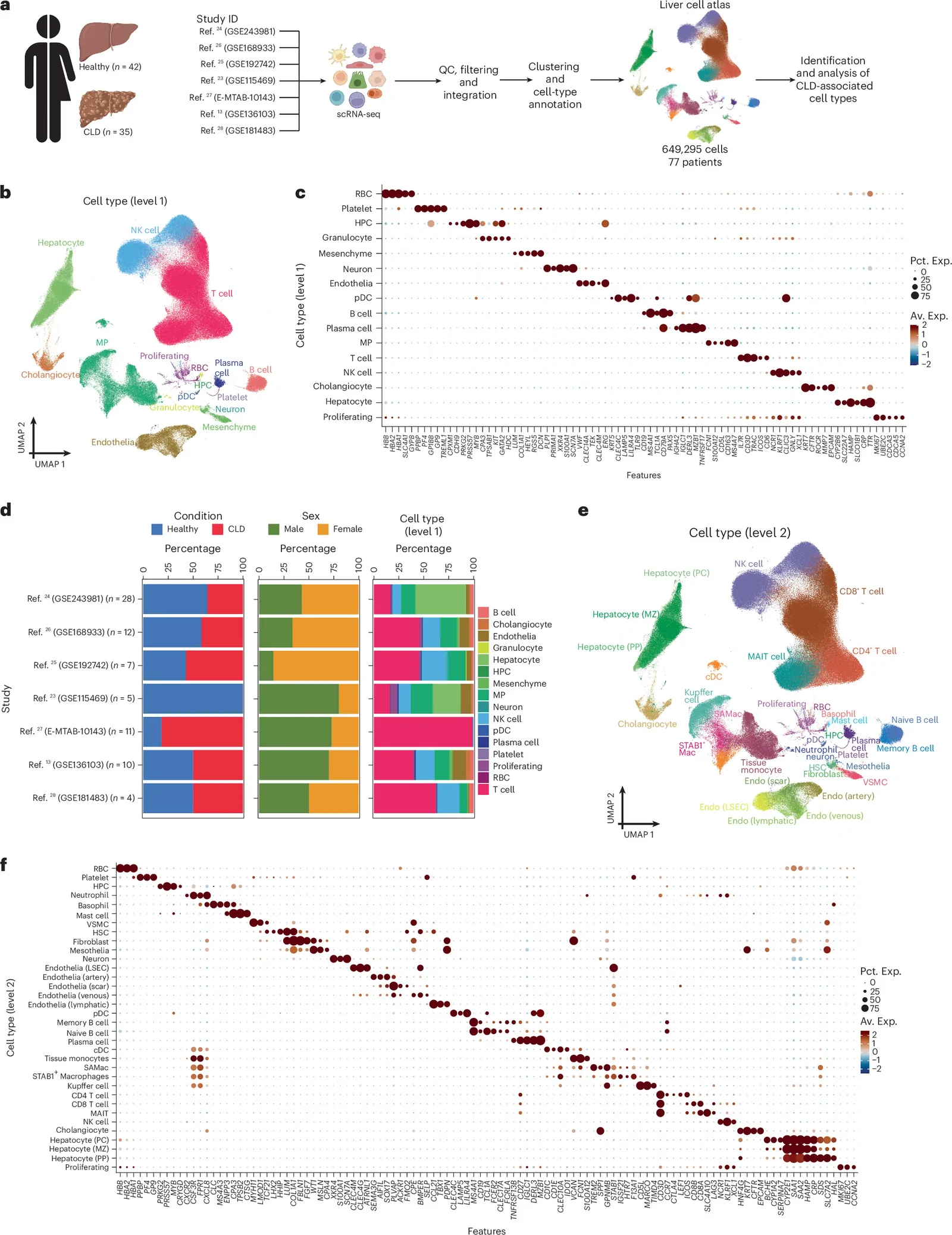
Developing a human liver single-cell atlas. **a**, Overview of analytical workflow for scRNA-seq atlas generation. Schematic in **a** created in BioRender; Colella, F. https://BioRender.com/1ejtusn (2025). **b**, UMAP of scRNA-seq data (649,295 cells; 77 patients) colored and labeled by level 1 annotation. **c**, Dot plot showing mean expression (Av. Exp.) of marker genes and percentage of cells expressing (Pct. Exp.) them for each level 1 cell type. **d**, Stacked bar plots of proportions of patients of each condition (left: healthy, blue and CLD, red), patients of each sex (middle; male, green and female, yellow) and cells of each level 1 cell type (right) across the seven scRNA-seq studies. **e**, UMAP of scRNA-seq data (649,295 cells; 77 patients) colored and labeled by level 2 annotation. **f**, Dot plot showing mean expression of marker genes (Av. Exp.) and percentage of cells expressing (Pct. Exp.) them for each level 2 cell type. RBC, red blood cell; pDC, plasmacytoid dendritic cell; HPC, hematopoietic progenitor cell; Endo, endothelial cell; VSMC, vascular smooth muscle cell; PC, pericentral; MZ, midzonal; PP, periportal; Av. Exp., average expression; Pct. Exp., percent expressed; UMAP, uniform manifold approximation and projection.

We conducted integrated analysis of seven published human scRNA-seq studies (Fig. 1a and Supplementary Table 1)^13,23–28^, combining data from 77 (42 healthy and 35 CLD) patients, derived from 38 (21 healthy and 17 CLD) males and 39 (21 healthy and 18 CLD) females. After quality control (QC), iterative subclustering and manual cluster annotation (Supplementary Note 1), we established a final level 3 liver atlas containing 649,295 single cells.

To facilitate downstream applications, three levels of atlas annotation were established, based on the transcriptional similarity across level 3 clusters (Supplementary Fig. 2a–c). Broad level 1 clusters consisted of 16 cell types (Fig. 1b,c and Supplementary Table 2). All studies contributed to level 1 annotation, with expected study-specific differences based on cell isolation methods from the original publications, such as more epithelial cells in the studies from refs. ^24,23^, and only T cells from the study discussed in ref. ^27^ (Fig. 1d). Apart from the study discussed in ref. ^23^, all studies contained a mixture of healthy individuals and patients with CLD. All studies contributed both male and female donors to the atlas (Fig. 1d).

**Extended Data Fig. 1:**
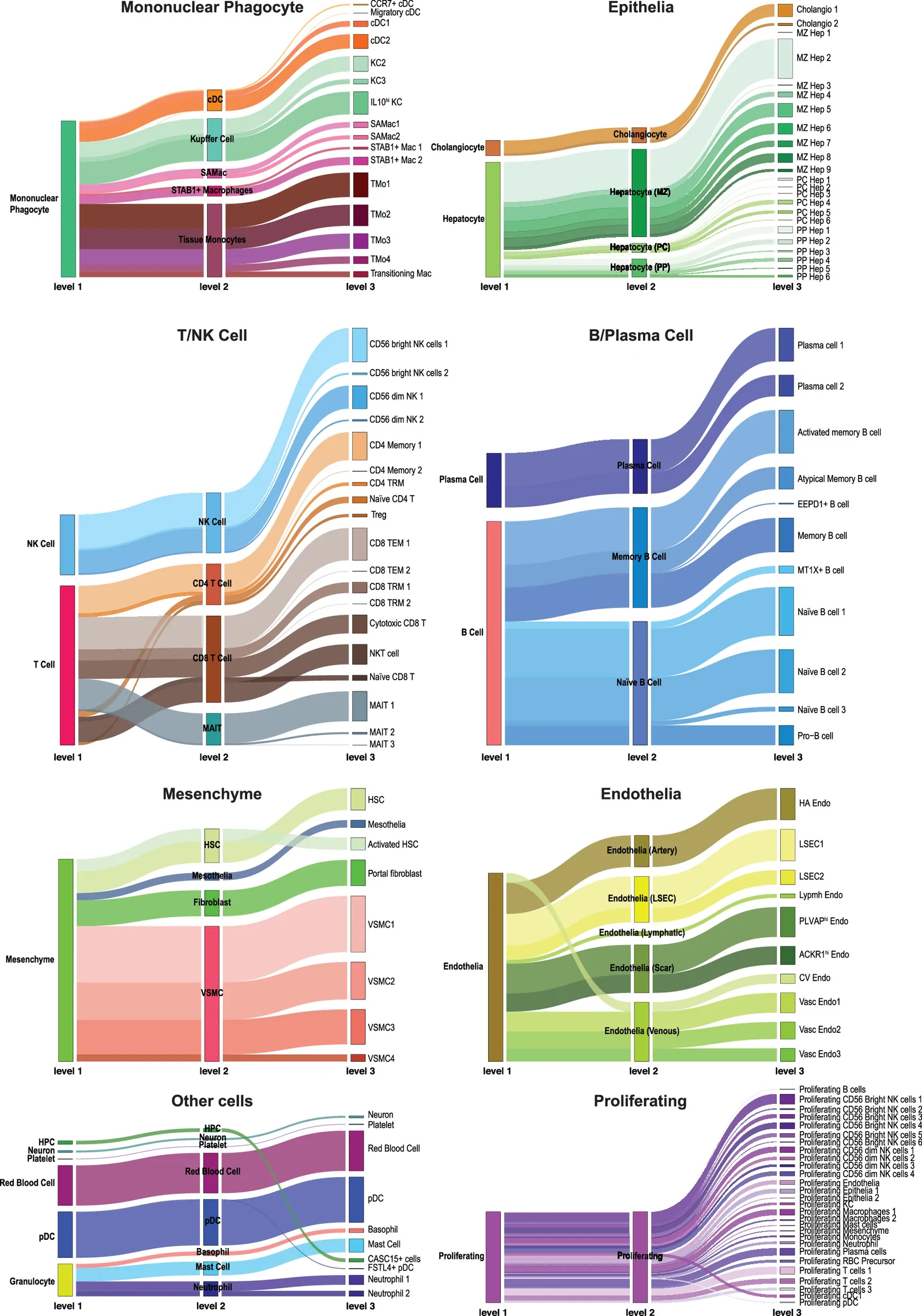
Structure of human liver single-cell atlas. Chord diagrams showing hierarchical structure of human liver single-cell atlas. Level 1 annotation represents broad cell lineages (16 cell types) that are subdivided into a moderate degree of cell type specificity (level 2 annotation; 34 cell types), which are further subdivided into a fine-grained annotation of different cell states (level 3 annotation; 125 cell states).

Intermediate level 2 annotation resulted in 34 cell types (Fig. 1e,f and Supplementary Table 3). Fine-grained unsupervised level 3 clustering resulted in a total of 125 final cell states (Supplementary Fig. 2c and Supplementary Table 4). Overall, atlas structure and hierarchy are summarized in Extended Data Fig. 1. Atlas gene expression profiles can be browsed at https://shiny.igc.ed.ac.uk/Human_Liver_scRNA-seq_Atlasv2/.

Notably, our approach identified rare cell types such as neurons, mast cells, basophils, neutrophils and hematopoietic progenitor cells (Fig. 1e,f), which are not well described in individual scRNA-seq studies. Level 3 clusters from each cell lineage were then used to assess changes in cell-type composition and gene expression between healthy and CLD liver tissue.

### Identifying SAMac heterogeneity in human CLD

**Fig. 2:**
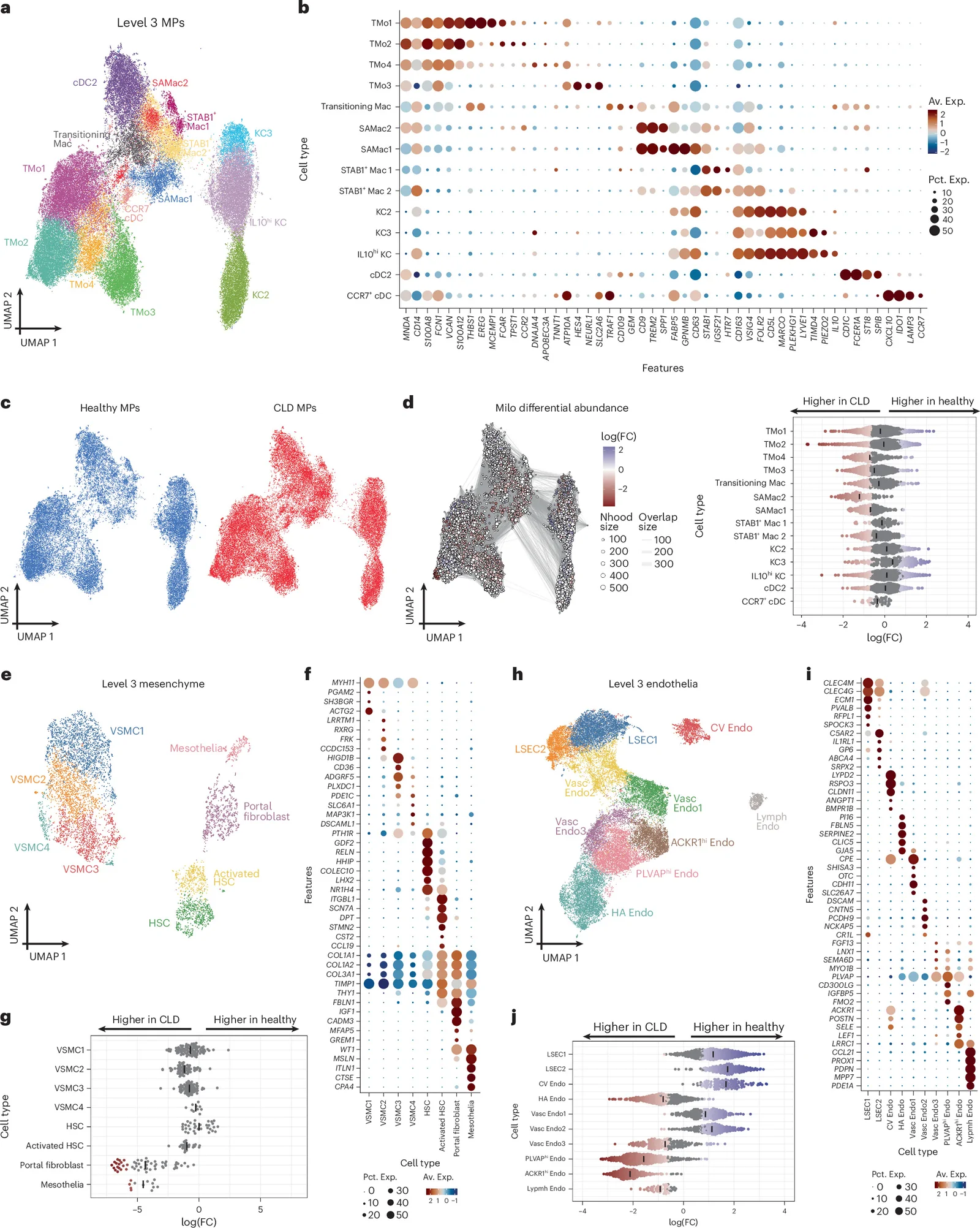
Defining changes in nonparenchymal cell composition in CLD. **a**, UMAP of MPs (81,577 cells) colored and labeled by level 3 annotation. **b**, Dot plot showing mean expression of marker genes and percentage of cells expressing them for each level 3 MP cell type. **c**, UMAP of MPs colored by patient condition. **d**, Milo cell neighborhood differential abundance plots (left) of the significant (FDR < 0.05) changes in the MP composition between healthy (*n* = 39) and CLD (*n* = 26). Color gradient scale represents log(FC). Blue represents enrichment in healthy, while red denotes expansion in CLD. Beeswarm plot (right) of the log(FC) in the Milo neighborhoods with CLD status grouped into each level MP cell-type subcluster. Neighborhoods with a significant change in cellular abundance are colored as indicated. Black lines denote mean log(FC) for each cell type. **e**, UMAP of mesenchymal cells (7,301 cells) colored and labeled by level 3 annotation. **f**, Dot plot showing mean expression of marker genes and percentage of cells expressing them for each level 3 mesenchymal cell type. **g**, Beeswarm plot of the log(FC) in Milo cell neighborhoods in mesenchymal cells between healthy (*n* = 27) individuals and patients with CLD (*n* = 22). Milo neighborhoods grouped into each level 3 mesenchymal cell-type subcluster. Neighborhoods with a significant (FDR < 0.05) change in cellular abundance are colored blue, representing enrichment in healthy, and red, denoting expansion in CLD. Black lines denote mean log(FC) for each cell type. **h**, UMAP of endothelial cells (36,591 cells) colored and labeled by level 3 annotation. **i**, Dot plot showing mean expression of marker genes and percentage of cells expressing them for each level 3 endothelial cell type. **j**, Beeswarm plot of the log(FC) in Milo cell neighborhoods in endothelial cells between healthy (*n* = 33) individuals and patients with CLD (*n* = 24). Milo neighborhoods grouped into each level 3 endothelial cell-type subcluster. Neighborhoods with a significant (FDR < 0.05) change in cellular abundance are colored, with blue representing enrichment in healthy and red denoting expansion in CLD. Black lines denote mean log(FC) for each cell type. TMo, tissue monocyte; log(FC), log fold change; LSEC, liver sinusoidal endothelial cell; CV, central vein; HA, hepatic artery; lymph, lymphatic; Vasc endo, vascular endothelial cell; FDR, false discovery rate.

**Extended Data Fig. 2:**
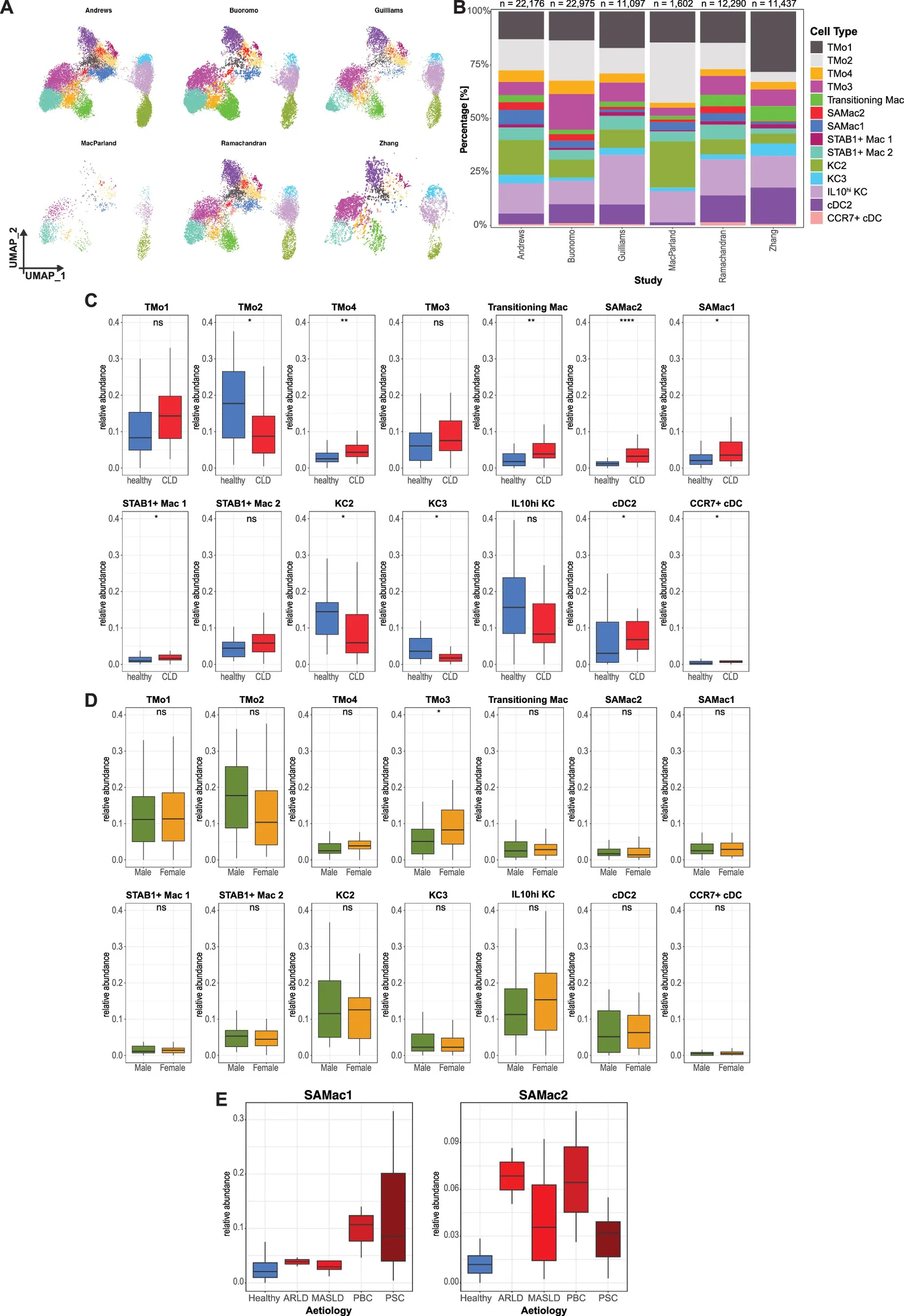
SAMac subpopulations expand in CLD livers. **a**, UMAPs of mononuclear phagocytes (81,577 cells) colored and labeled by level 3 annotation. UMAPs split by original study. **b**, Stacked bar plots of proportions of cells of each level 3 MP cell type across the scRNA-seq studies. Bars colored by level 3 MP cell type. Total number of MPs from each study is stated at the top of each bar. **c**, Differential abundance analysis of each level 3 MP subtype between healthy (blue bars; *n* = 38) and CLD (red bars; *n* = 25) patients across all of the samples using two-sided Wilcoxon rank-sum tests. Box plots show the median (centerline), first and third quartiles (lower and upper box limits), and whiskers extending to the smallest and largest observations within 1.5× interquartile range (IQR). *P < 0.05, **P < 0.01, ****P < 0.0001, ns *P* ≥ 0.05. **d**, Differential abundance analysis of each level 3 MP subtype between male (green bars, *n* = 29) and female (yellow bars, *n* = 34) patients across all of the samples using two-sided Wilcoxon rank-sum tests. Box plots show the median (centerline), first and third quartiles (lower and upper box limits), and whiskers extending to the smallest and largest observations within 1.5× IQR. *P < 0.05, ns *P* ≥ 0.05. **e**, Differential abundance of SAMac1 and SAMac2 between healthy (blue) patients and patients with different etiologies of CLD (red). Box plots show the median (centerline), first and third quartiles (lower and upper box limits), and whiskers extending to the smallest and largest observations within 1.5× IQR. Healthy *n* = 38; ARLD (alcohol-related liver disease) *n* = 2; MASLD (metabolic dysfunction-associated steatotic liver disease) *n* = 4; PBC (primary biliary cholangitis) *n* = 3, PSC (primary sclerosing cholangitis) *n* = 8.

To study MP changes in CLD, we analyzed level 3 atlas data. In total, 81,577 nonproliferating MPs were included, partitioned into 14 clusters (Fig. 2a). MP subpopulations were detected from each study, apart from the T-cell-focused study in ref. ^27^ (Extended Data Fig. 2a,b). MP clusters could broadly be separated into four *MNDA*^+^ *FCN1*^+^ tissue monocyte subpopulations (TMo1–4), two conventional dendritic cell (cDC) populations (*CD1C*^+^ *FCER1A*^+^ cDC2 and migratory *IDO1*^+^ *CCR7*^+^ cDC), three *CD5L*^+^ *MARCO*^+^ Kupffer cell (KC) populations (IL10^hi^ KCs that are similar to recently described periportal KCs^29^, *TIMD4*^−^ *PIEZO2*^−^ KC2 and *TIMD4*^+^ *PIEZO2*^+^ KC3) and two macrophage populations that were high in *STAB1* and *IGSF21* and reminiscent of monocyte-derived KCs in mice^25^. Furthermore, we distinguished two distinct populations of macrophages that expressed high levels of previously defined SAMac marker genes *TREM2*, *CD9*, *CD63* and *SPP1* (refs. ^13,14^; Fig. 2b and Supplementary Table 5). These subpopulations were annotated SAMac1 and SAMac2.

Differential abundance analysis using the Milo package^30^ demonstrated that both SAMac1 and SAMac2 expanded in CLD livers compared to healthy livers (Fig. 2c,d), a finding we confirmed using an alternative statistical approach (Extended Data Fig. 2c). No differences were observed in MP composition between male and female patients (Extended Data Fig. 2d). SAMac2 expansion was consistent across all etiologies of CLD, while SAMac1 expansion was more notable in patients with the biliary disorders primary biliary cholangitis (PBC) and primary sclerosing cholangitis (PSC; Extended Data Fig. 2e). These data suggest that expansion of the SAMac2 population is a common feature of CLD.

### Annotation of other hepatic cell types

To identify other CLD-associated cell states, we replicated the level 3 analysis for other major cell lineages. A total of 7,301 mesenchymal cells were partitioned into eight clusters (Fig. 2e,f and Supplementary Table 6). We resolved four distinct subpopulations of *MYH11*^+^ vascular smooth muscle cells, *LHX2*^+^ *NR1H4*^+^ quiescent and activated hepatic stellate cells (HSC), *THY1*^+^ *FBLN1*^+^ *IGF1*^+^ portal fibroblasts and *MSLN*^+^ mesothelial cells (Fig. 2f). Differential abundance analysis demonstrated expansion of portal fibroblasts in CLD (Fig. 2g). This population, along with activated HSCs, expressed the highest levels of fibrillar collagens (*COL1A1*, *COL1A2*, *COL3A1*; Fig. 2f), suggesting that they are the main scar-producing populations in the CLD liver.

Vascular changes are a characteristic feature of advanced CLD and regulate fibrosis^13,31,32^. A total of 36,591 liver endothelial cells were partitioned into ten clusters (Fig. 2h). Distinguishing marker genes (Fig. 2i and Supplementary Table 7) enabled annotation of liver sinusoidal endothelial cells, *RSPO3*^+^ central vein endothelia, *FBLN5*^+^ hepatic artery endothelia, *CCL21*^+^ *PDPN*^+^ lymphatic endothelia and three additional subpopulations of vascular endothelial cells (Vasc. endo 1–3). We also confirmed the presence of *PLVAP*^hi^ and *ACKR1*^hi^ disease-associated endothelial cells, as previously described^13^. Milo analysis demonstrated expansion of arterial, lymphatic, PLVAP^hi^ and ACKR1^hi^ subpopulations in CLD, as well as a reduction in homeostatic sinusoidal and vascular subpopulations (Fig. 2j).

**Extended Data Fig. 3:**
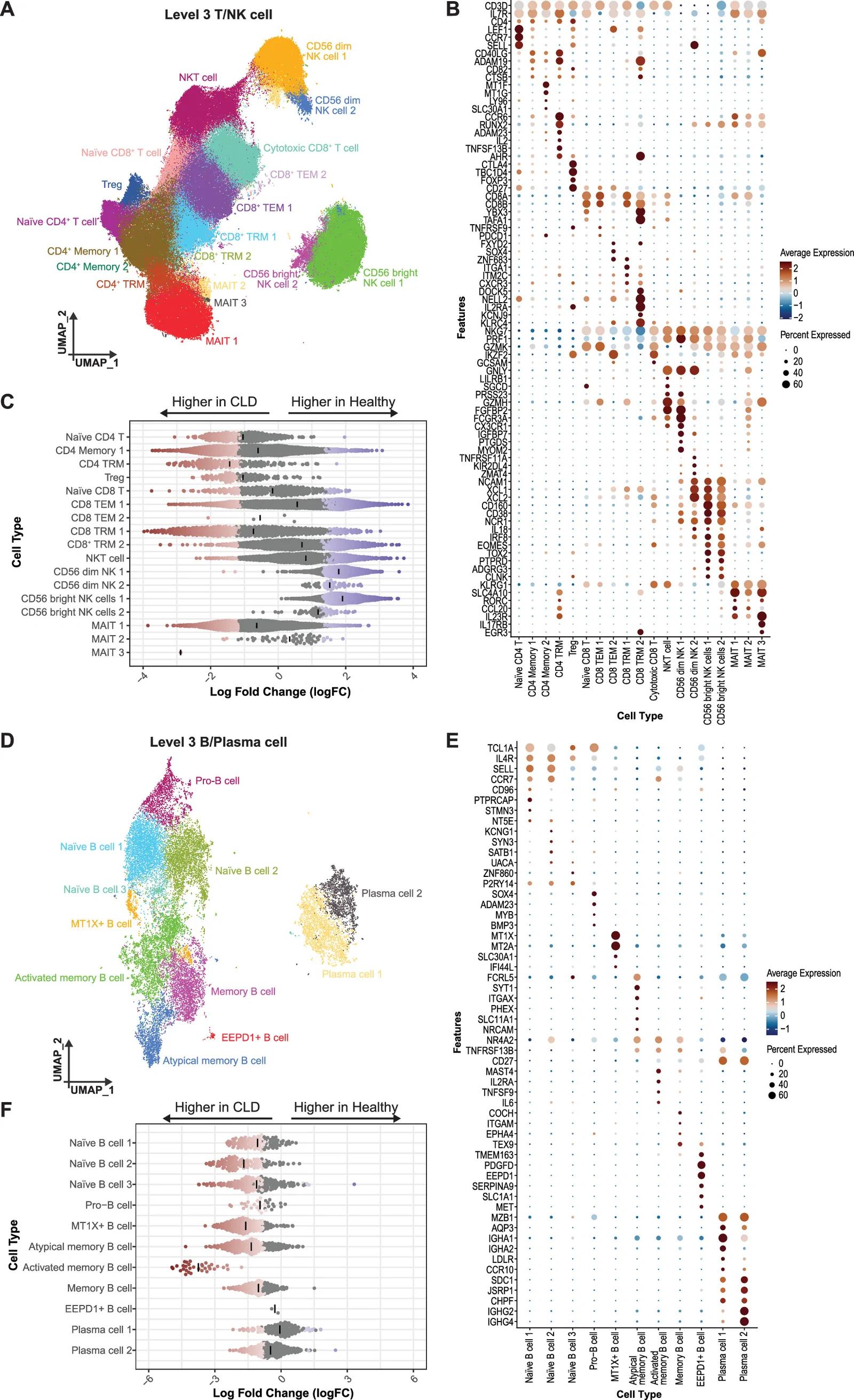
Alterations in hepatic lymphoid cells in CLD patients. **a**, UMAP of T/NK cells (393,035 cells) colored and labeled by level 3 annotation. **b**, Dot plot showing mean expression of marker genes and percentage of cells expressing them for each level 3 T/NK cell type. **c**, Beeswarm plot of the log fold changes in Milo cell neighborhoods in T/NK cells between healthy (*n* = 35) and CLD (*n* = 37) patients. Milo neighborhoods grouped into each level 3 T/NK cell type subcluster. Neighborhoods with a significant (FDR < 0.05) change in cellular abundance are colored, with blue representing enrichment in healthy and red denoting expansion in CLD. Black lines denote mean logFC for each cell type. **d**, UMAP of B/plasma cells (22,854 cells) colored and labeled by level 3 annotation. **e**, Dot plot showing mean expression of marker genes and percentage of cells expressing them for each level 3 B/plasma cell type. **f**, Beeswarm plot of the log fold changes in Milo cell neighborhoods in B/plasma cells between healthy (*n* = 32) and CLD (*n* = 26) patients. Milo neighborhoods grouped into each level 3 B/plasma cell type subcluster. Neighborhoods with a significant (FDR < 0.05) change in cellular abundance are colored, with blue representing enrichment in healthy and red denoting expansion in CLD. Black lines denote mean log(FC) for each cell type.

We resolved 19 subpopulations of T/NK cells (Extended Data Fig. 3a,b and Supplementary Table 8). Identified T cell subpopulations included naive, memory, tissue resident memory and regulatory (Treg) CD4^+^ T cells (Extended Data Fig. 3b). Similar heterogeneity was identified in the CD8^+^ T cell compartment, with subpopulations of naive, effector memory (T_EM_), resident memory and cytotoxic cells (Extended Data Fig. 3b). We also defined subpopulations *GNLY*^+^ CD56^dim^ NK cells, *CD160*^+^ *EOMES*^+^ CD56^bright^ NK cells and three clusters of *SLC4A10*^+^ *IL23R*^+^ mucosal-associated invariant T cells (Extended Data Fig. 3b). Differential abundance analysis showed a loss of NK and cytotoxic CD8^+^ T cells and shift toward a CD4-rich environment with expansion of CD4^+^ naive, CD4^+^ tissue resident memory and Tregs in CLD (Extended Data Fig. 3c).

Heterogeneity of the hepatic B cell compartment was also observed, with distinct subpopulations of *TCL1A*^+^ *IL4R*^+^ naive B cells, *NR4A2*^+^ *TNFRSF13B*^+^ memory B cells and two clusters of *MZB1*^+^ *SDC1*^+^ plasma cells (Extended Data Fig. 3d,e and Supplementary Table 9). All naive and memory B cell subpopulations expanded in CLD livers, most strikingly *IL6*^+^ *MAST4*^+^ activated memory B cells (Extended Data Fig. 3f).

**Extended Data Fig. 4:**
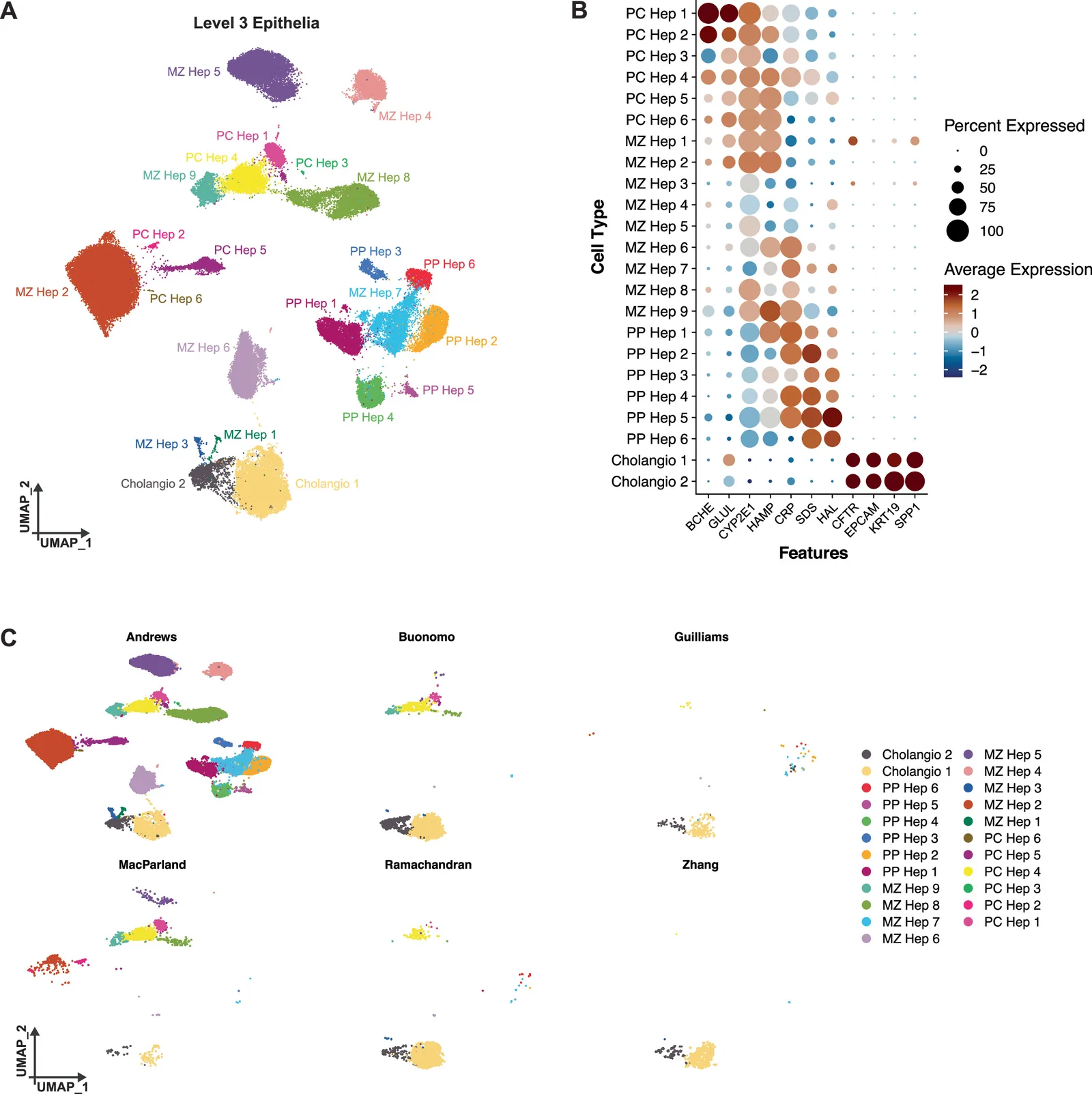
Defining liver epithelial cell heterogeneity. **a**, UMAP of liver epithelial cells (91,716 cells) colored and labeled by level 3 annotation. PP, periportal; MZ, midzonal; PC, pericentral; Hep, hepatocyte; Cholangio, cholangiocyte. **b**, Dot plot showing mean expression of marker genes and percentage of cells expressing them for each level 3 epithelial cell type. **c**, UMAPs of epithelial cells colored and labeled by level 3 annotation. UMAPs split by original study.

Finally, we annotated epithelial (hepatocyte and cholangiocyte) subpopulations and identified gene expression patterns indicative of hepatocyte zonation (Extended Data Fig. 4a,b and Supplementary Table 10). Hepatocytes were mainly derived from the studies in refs. ^23,24^ (Extended Data Fig. 4c), so conclusions about CLD-associated changes in hepatocyte composition or transcription cannot be drawn. Cholangiocytes were captured from all the included studies (Extended Data Fig. 4c).

### OLR1^+^ SAMac2 has a more pro-inflammatory pro-fibrotic profile

**Fig. 3:**
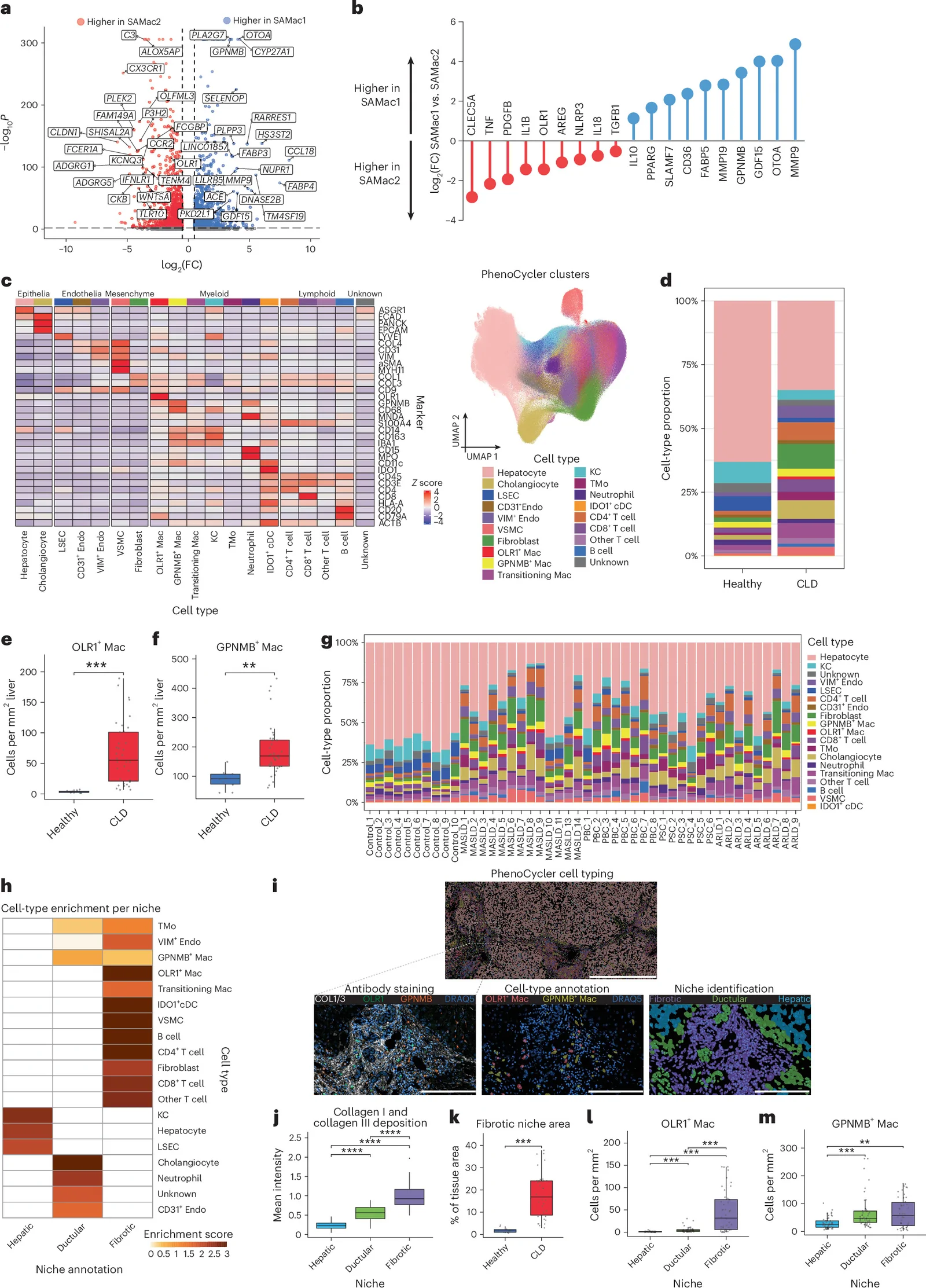
High *OLR1* expression distinguishes pro-inflammatory SAMacs that spatially accumulate in the CLD fibrotic niche. **a**, Volcano plot of differential gene expression between SAMac1 and SAMac2 in the level 3 MP atlas. Two-sided MAST hurdle model. *P* values adjusted for multiple testing using Bonferroni correction. Significant genes (log_2_(FC) > 0.5 and *P*_adj_ < 0.01) colored in blue if enriched in SAMac1 and red if enriched in SAMac2. Selected genes are labeled. **b**, log_2_(FC) plot of selected significant genes from **a**. Circles in red are genes enriched in SAMac2 and circles in blue are genes enriched in SAMac1. **c**–**m**, Analysis of PCF spatial proteomics data from 10 healthy and 36 CLD patient livers. **c**, Heatmap (right) and UMAP (left) showing annotated liver cell types and marker expression. **d**, Stacked bar plot of proportions of each PCF-annotated cell type in healthy versus CLD livers. Color scale as in **c**. **e**,**f**, Box and whiskers plots of OLR1^+^ Mac (**e**), and GPNMB^+^ Mac (**f**), cell number per mm^2^ liver tissue from PCF data, comparing healthy (*n* = 10) versus CLD (*n* = 36) livers for each cell type. Box plots show median (centerline), first and third quartiles (lower and upper box limits) and 1.5× IQR (whiskers). Individual dots represent each patient. Two-sided *t* test; **P < 0.01 (*P* = 0.0015), ***P < 0.001 (*P* = 0.0004). **g**, Stacked bar plot of proportions of PCF-annotated cell types in each patient, grouped by etiology of CLD. **h**, Cellular neighborhood analysis on PCF data using scNiche distinguishes three niches. Heatmap showing enrichment score of cell types in each niche. **i**, Representative images from PCF analysis of CLD liver tissue. Top: low-magnification image (scale bar = 1,000 µm) showing cell-type mapping, colored as in **c**. Bottom: high-magnification images (scale bar = 200 µm) showing selected antibody staining (left), mapping of OLR1^+^ Mac in red and GPNMB^+^ Mac in yellow (middle) and niche identification (right). **j**, Box and whiskers plots of combined collagen I and collagen III mean fluorescence staining intensity per patient in each spatial niche across all PCF samples (*n* = 46). Box plots show median (centerline), first and third quartiles (lower and upper box limits) and 1.5× IQR (whiskers). KW test with pairwise Wilcoxon and BH correction; ****P < 0.0001. **k**, Box and whiskers plots of area of the fibrotic niche as a percentage of total liver area comparing healthy (*n* = 10) individuals versus patients with CLD (*n* = 36). Dots represent each patient. Box plots show median (centerline), first and third quartiles (lower and upper box limits) and 1.5× IQR (whiskers). Two-sided Wilcoxon rank-sum test; ***P < 0.001 (*P* = 3.43 × 10^−9^). **l**, Box and whiskers plot of OLR1^+^ Mac cell number per mm^2^ tissue in each niche across all PCF samples (*n* = 46). **m**, Box and whiskers plots of GPNMB^+^ Mac cell number per mm^2^ tissue in each niche across all PCF samples (*n* = 46). For **l** and **m**, box plots show median (centerline), first and third quartiles (lower and upper box limits) and 1.5× IQR (whiskers); dots represent each patient; KW test with pairwise Wilcoxon and BH correction; **P < 0.01 ***P < 0.001. IQR, interquartile range; KW, Kruskal–Wallis; MAST, model-based analysis of single-cell transcriptomics. Source data

**Extended Data Fig. 5:**
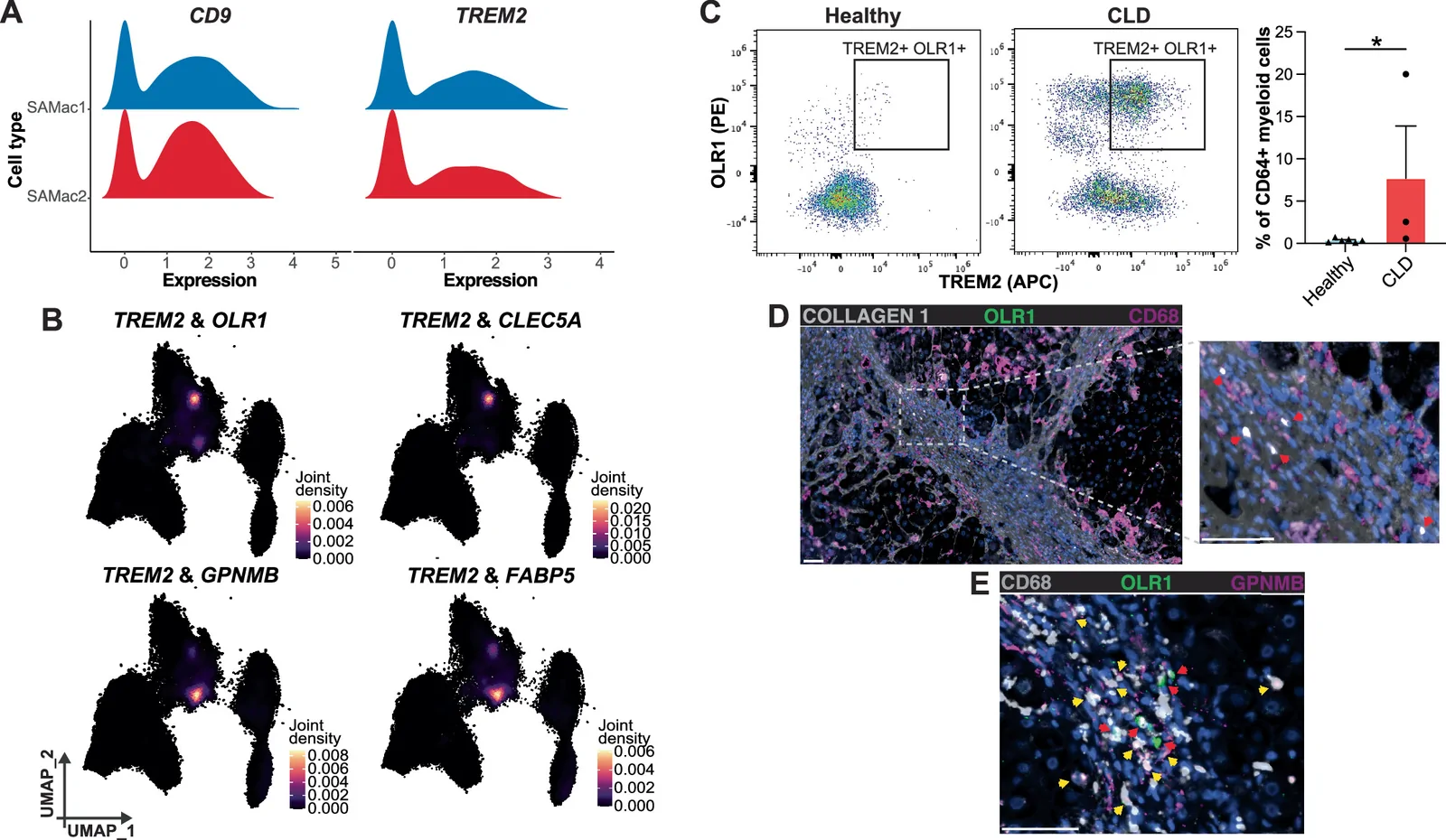
OLR1 and GPNMB expression distinguish SAMac subpopulations. **a**, Ridge plots showing scaled gene expression of *CD9* and *TREM2* across SAMac1 or SAMac2 cells. **b**, Density plots showing joint expression of stated genes in level 3 MP atlas. **c**, Flow cytometry of healthy and CLD human liver tissue. Representative plots of TREM2 versus OLR1 expression, pregated on viable CD64^+^ myeloid cells (left). Quantitation of TREM2^+^ OLR1^+^ SAMacs in healthy (*n* = 6) versus CLD (*n* = 3) livers. Bars indicate mean ± s.e.m. Two-sided Mann–Whitney test; *P < 0.05 (*P* = 0.0476). **d**,**e**, Representative immunofluorescence images of human liver fibrotic niche. For **d**, collagen 1 (gray), CD68 (magenta), OLR1 (green), DAPI (blue); red arrows indicate OLR1^+^ CD68^+^ SAMacs. For **e**, CD68 (gray), GPNMB (magenta), OLR1 (green), DAPI (blue); red arrows indicate OLR1^+^ CD68^+^ SAMacs; yellow arrows indicate GPNMB^+^ CD68^+^ SAMacs. For **d** and **e**, images are representative of 3 independent patient samples; scale bars = 50 μm. Source data

Having identified two subpopulations of *TREM2*^+^ *CD9*^+^ SAMac, we defined the transcriptional differences between SAMac1 and SAMac2, showing 903 genes significantly enriched in SAMac1 and 1,176 genes in SAMac2 (Fig. 3a and Supplementary Table 11). SAMac1 expressed higher levels of *GPNMB* as well as several anti-inflammatory antifibrotic factors such as *MMP9*, *IL10*, *GDF15*, *CD36* and *PPARG* (Fig. 3b). SAMac1 is therefore transcriptionally similar to lipid-associated macrophages described in mice^21,33^ and humans^34^. In contrast, SAMac2 demonstrated higher expression of pro-inflammatory and pro-fibrotic mediators (*IL1B*, *TNF*, *IL18*, *NLRP3*, *AREG*, *PDGFB*), and scavenger receptors including *OLR1* and *CLEC5A* (Fig. 3b). No differences in *TREM2* or *CD9* expression were observed between SAMac1 and SAMac2 (Extended Data Fig. 5a), but SAMac2 showed coexpression of *TREM2*/*OLR1* or *TREM2*/*CLEC5A*, while SAMac1 coexpressed TREM2/GPNMB or TREM2/FABP5 (Extended Data Fig. 5b). Flow cytometry confirmed expansion of TREM2^+^ OLR1^+^ macrophages in CLD livers (Extended Data Fig. 5c). Immunofluorescence staining indicated that OLR1^+^ CD68^+^ cells were spatially located in areas of fibrosis in CLD and represented a subpopulation of CD68^+^ SAMacs (Extended Data Fig. 5d). OLR1^+^ CD68^+^ SAMac2 (Extended Data Fig. 5e, red arrows) was distinct from GPNMB^+^ CD68^+^ SAMac1 (Extended Data Fig. 5e, yellow arrows), emphasizing SAMac heterogeneity.

**Extended Data Fig. 6:**
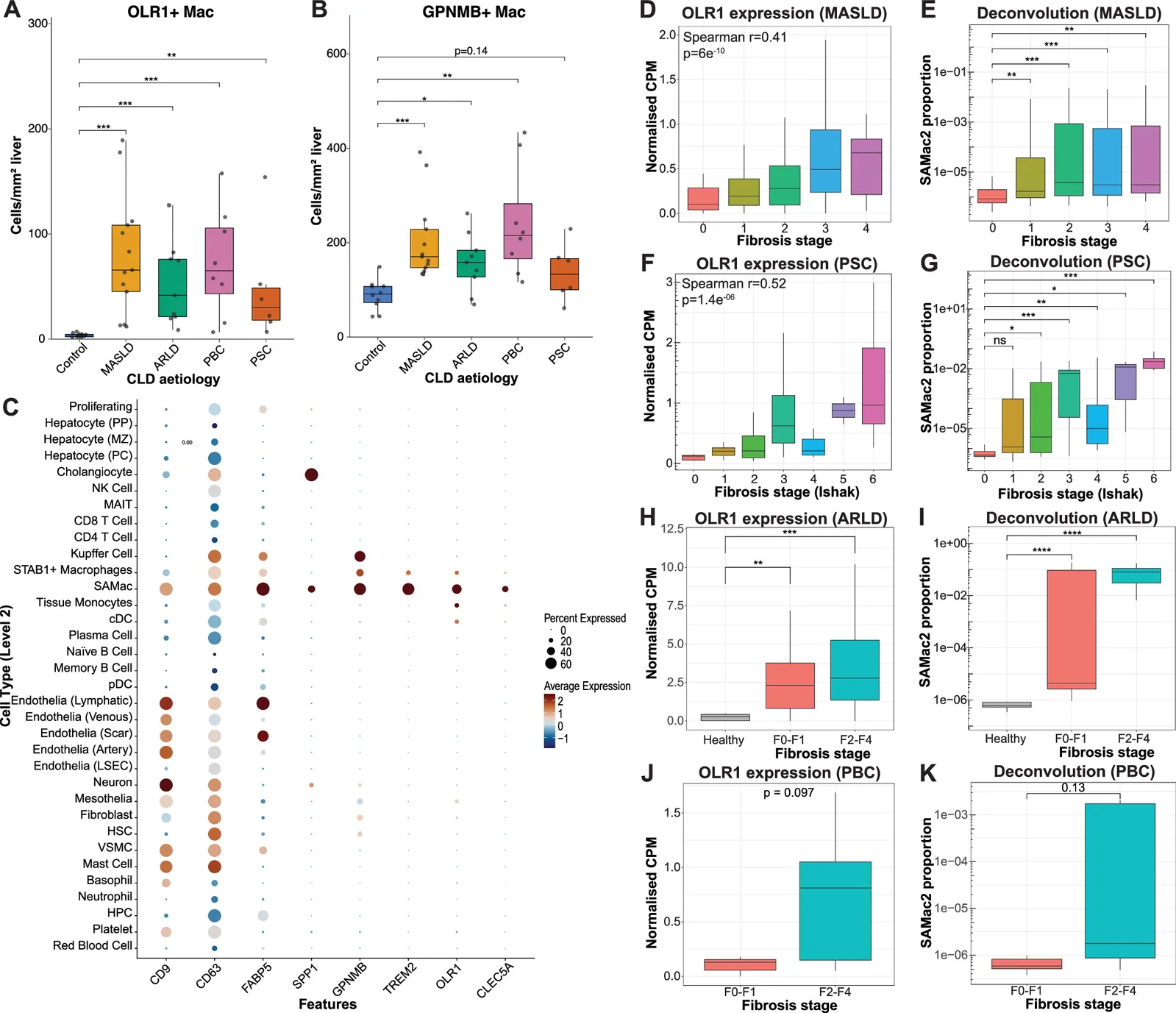
Increased OLR1 expression and SAMac2 expansion are conserved across multiple CLD etiologies. **a**,**b**, PhenoCycler-Fusion quantiation of OLR1^+^ Mac (**a**), and GPNMB^+^ Mac (**b**), cell number per mm^2^ liver tissue, comparing healthy (*n* = 10) versus CLD livers of differing etiologies (MASLD = 13; ARLD = 9; PBC = 8; PSC = 6). Box plots show median (centerline), first and third quartiles (lower and upper box limits), 1.5× interquartile range (whiskers). Dots represent patients. Kruskal–Wallis test with pairwise Wilcoxon and BH correction; *P < 0.05, **P < 0.01 ***P < 0.001. **c**, Dot plot showing mean expression of stated genes and percentage of cells expressing them for each level 2 cell type. **d**,**e**, Analysis of bulk liver RNA-seq data from 206 MASLD patients stratified by fibrosis (F) stage (*n* = 38 (F0), 47 (F1), 53 (F2), 54 (F3), 14 (F4))^37^. *OLR1* gene expression (normalized counts per million (CPM); **d**), and SAMac2 proportion (derived from deconvolution; **e**), across fibrosis stages. For **d**, two-sided Spearman correlation of normalized *OLR1* CPM versus F-stage. For **e**, Kruskal–Wallis test with pairwise Wilcoxon and BH correction, **P < 0.01, ***P < 0.001. **f**,**g**, Analysis of bulk liver RNA-seq data from 75 PSC cases stratified by Ishak fibrosis stage (*n* = 8 (F0), 18 (F1), 16 (F2), 15 (F3), 7 (F4), 3 (F5), 8 (F6))^38^. *OLR1* gene expression (**f**) and deconvolution-derived SAMac2 proportion (**g**), according to fibrosis stage. For **f**, two-sided Spearman correlation of normalized OLR1 CPM versus F-stage. For **g**, two-sided pairwise Wilcoxon rank-sum tests and BH correction, *P < 0.05, **P < 0.01, **P < 0.001. **h**,**i**, Analysis of liver bulk RNA-seq data from 59 patients (*n* = 10 healthy, 11 ARLD F0-F1, 38 ARLD F2-F4)^39^. *OLR1* gene expression (**h**) and deconvolution-derived SAMac2 proportion (**i**), according to conditon and fibrosis stage. For **h** and **i**, two-sided pairwise Wilcoxon rank-sum tests and BH correction, **P < 0.01, ***P < 0.001, ****P < 0.0001. **j**,**k**, Analysis of liver bulk RNA-seq data from 14 PBC patients stratified by fibrosis stage (*n* = 7 F0–F1, 7 F2–F4)^40^. *OLR1* gene expression (**j**) and deconvolution-derived SAMac2 proportion (**k**), according to fibrosis stage. For **j** and **k**, two-sided Wilcoxon rank-sum test. For **d**–**k**, box plots show median (centerline), first and third quartiles (lower and upper box limits) and 1.5× interquartile range (whiskers). Source data

To define the spatial distribution of SAMac subpopulations, we performed spatial proteomics on 46 patient liver samples (10 healthy (8 resection and 2 biopsy) and 36 CLD (9 metabolic dysfunction-associated steatotic liver disease (MASLD) explant, 4 MASLD biopsy, 9 alcohol-related liver disease (ARLD) explant, 8 PBC explant and 6 PSC explant)) through a 36-plex antibody panel on the PhenoCycler-Fusion (PCF). We annotated major liver cell types including OLR1^+^ and GPNMB^+^ macrophages (Fig. 3c). Assessment of cellular composition across patients demonstrated loss of hepatocytes and expansion of fibroinflammatory cells in CLD (Fig. 3d). Both OLR1^+^ and GPNMB^+^ macrophages accumulated significantly in CLD (Fig. 3e,f), with no differences in abundance observed between different etiologies (Fig. 3g and Extended Data Fig. 6a,b). Neighborhood analysis identified three cellular niches, including a fibrotic niche that was enriched in fibroinflammatory cells (Fig. 3h,i), fibrillar collagen deposition (Fig. 3i,j) and expanded in CLD (Fig. 3k). Examination of OLR1 and GPNMB staining showed occasional double-positive cells but predominantly discrete expression, mirroring our scRNA-seq data (Fig. 3i). Both OLR1^+^ and GPNMB^+^ macrophages preferentially accumulated within the fibrotic niche (Fig. 3i,l,m). Hence, OLR1 and GPNMB label distinct macrophage subpopulations, both of which spatially accumulate in the CLD fibrotic niche.

### High OLR1 expression is associated with adverse clinical outcomes

OLR1 (LOX-1), a class E scavenger receptor, has been suggested as a therapeutic target for cardiovascular disease^35,36^. *OLR1* expression, alongside *CLEC5A*, was specific to MPs in the human liver and highly enriched in SAMacs (Extended Data Fig. 6c), suggesting that it may offer a route to selective targeting of SAMacs.

**Fig. 4:**
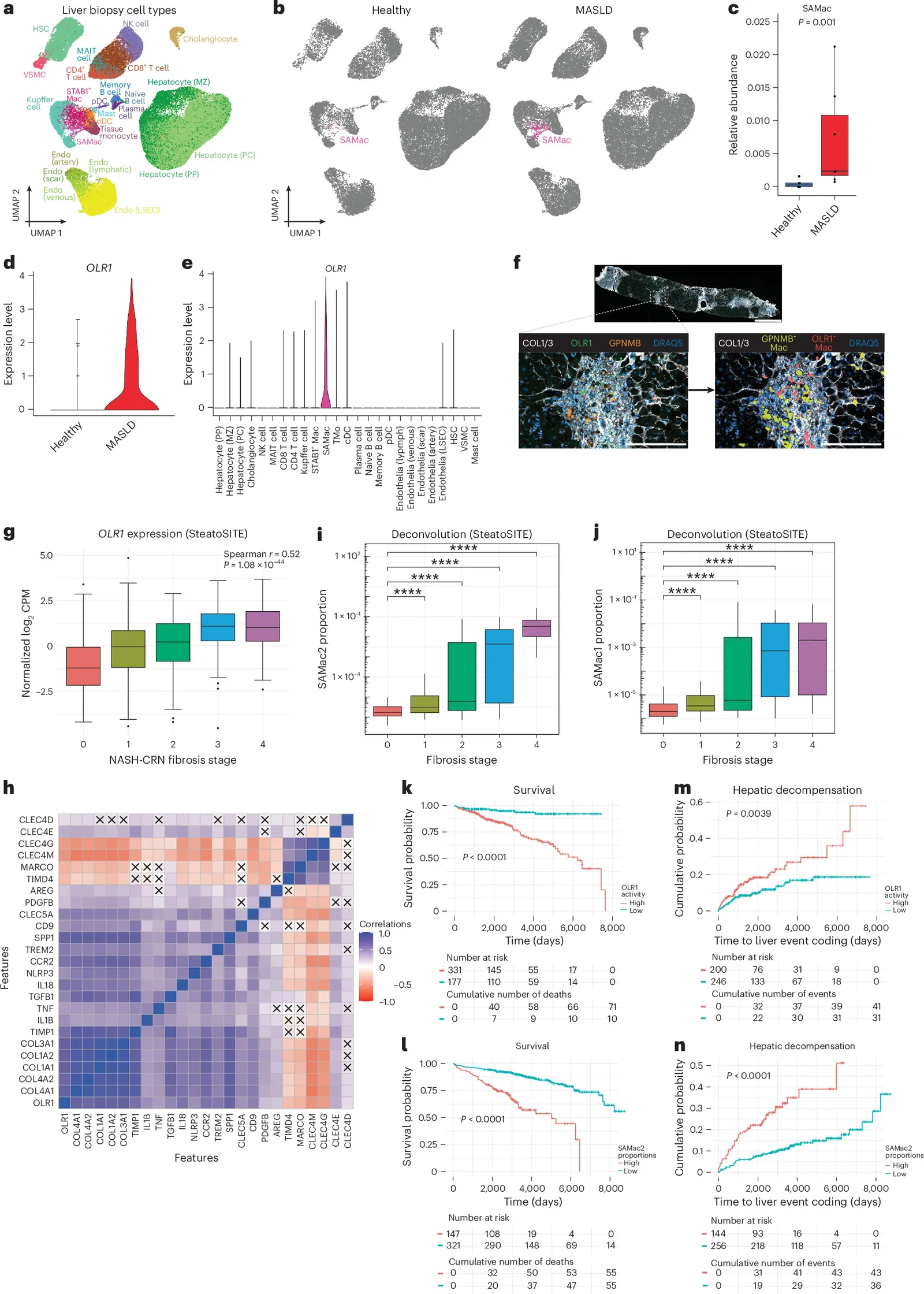
OLR1^+^ SAMacs expand progressively with fibrosis stage in MASLD and predict adverse disease outcomes. **a**–**e**, scRNA-seq analysis of liver needle biopsies from six healthy (histologically normal) and seven patients with MASLD. **a**, UMAP of 71,062 liver biopsy cells colored and labeled by cell-type annotation. **b**, UMAPs of biopsy scRNA-seq data split by stated condition. SAMac is labeled in pink. Other cell types are labeled in gray. **c**, Differential abundance analysis of SAMac is between healthy (*n* = 6) and MASLD (*n* = 7) patient biopsies. Box plots show median (centerline), first and third quartiles (lower and upper box limits) and highest and lowest values (whiskers). Empirical Bayes moderated *t* statistic test performed using propeller function from R package speckle. **d**, Violin plot of *OLR1* expression in SAMacs split by stated condition. **e**, Violin plot of *OLR1* expression in stated cell types from liver needle biopsies. **f**, Representative images from PCF analysis of MASLD liver needle biopsy tissue. Low-magnification image (top: scale bar = 1,000 µm) and high-magnification images (bottom: scale bars = 200 µm) showing selected antibody staining (left) and mapping of OLR1^+^ Mac in red and GPNMB^+^ Mac in yellow (right). Images representative of four independent patient biopsies. **g**, Box and whiskers plot of *OLR1* gene expression (shown as normalized log_2_ CPM) according to histological fibrosis stage (NASH-CRN) in 632 cases (*n* = 172 (F0), 134 (F1), 96 (F2), 111 (F3), 119 (F4)) from the SteatoSITE dataset. All box plots show median (centerline), first and third quartiles (lower and upper box limits), 1.5× IQR (whiskers) and outliers (dots). Two-sided Spearman correlation of normalized *OLR1* CPM versus ordinal NASH-CRN F stage of cases was calculated. **h**, Correlation matrix of normalized gene expression counts for stated genes in the complete *n* = 663 set of cases (including controls) with RNA-seq from the SteatoSITE dataset. Statistically significant correlations (*P* < 0.05) are shown as shaded boxes, and nonsignificant correlations are marked with an X. Boxes are colored by two-sided Spearman correlation coefficients (blue, positive and red, negative correlation). **i**, Box and whiskers plot of SAMac2 proportion according to histological fibrosis stage (NASH-CRN), derived from deconvolution analysis of bulk RNA-seq data from SteatoSITE. **j**, Box and whiskers plot of SAMac1 proportion according to the histological fibrosis stage (NASH-CRN), derived from deconvolution analysis of bulk RNA-seq data from SteatoSITE. For **i** and **j**, bulk RNA-seq of 632 SteatoSITE cases (*n* = 172 (F0), 134 (F1), 96 (F2), 111 (F3), 119 (F4)) is analyzed. Box plots show median (centerline), first and third quartiles (lower and upper box limits) and 1.5× IQR (whiskers). Two-sided pairwise Wilcoxon rank-sum tests and BH correction, ****P < 0.0001. **k**, Biopsy cases from the SteatoSITE dataset (*n* = 508) with available RNA-seq were used for time-to-all-cause mortality analysis. Normalized gene expression counts for *OLR1* was used to divide cases into low and high risk of death (Kaplan–Meier estimator curves with log-rank test *P* value shown). **l**, Biopsy cases from the SteatoSITE dataset with available RNA-seq and deconvolution data (*n* = 468) were used for time-to-all-cause mortality analysis. SAMac2 proportions were used to divide cases into low and high risk of death (Kaplan–Meier estimator curves with log-rank test *P* value shown). **m**, Biopsy cases from the SteatoSITE dataset (*n* = 446) with available RNA-seq and no hepatic decompensation-related coding event before the time of the biopsy. Normalized gene expression counts for *OLR1* were used to divide cases into low and high risk of hepatic decompensation (Kaplan–Meier estimator curves with log-rank test *P* value shown). **n**, Biopsy cases from the SteatoSITE dataset with available RNA-seq and deconvolution data and no hepatic decompensation-related coding event before the time of the biopsy (*n* = 400) were used for time-to-event analysis. SAMac2 proportions were used to divide cases into low and high risk of death (Kaplan–Meier estimator curves with log-rank test *P* value shown). Source data

To determine whether OLR1 could represent a therapeutic target across the CLD severity spectrum, we performed scRNA-seq on liver needle biopsies from patients with MASLD compared to those with histologically normal liver. A total of 71,062 cells from 13 patients (7 MASLD and 6 healthy; Supplementary Table 12) were annotated using our level 2 atlas (Fig. 4a). SAMacs expanded in MASLD biopsies (Fig. 4b,c) and upregulated *OLR1* (Fig. 4d). *OLR1* expression remained specific to macrophages (Fig. 4e). Similar to more advanced disease, PCF proteomic analysis confirmed that OLR1^+^ and GPNMB^+^ macrophages accumulate in the fibrotic niche of MASLD biopsies (Fig. 4f).

To assess OLR1 expression across disease stages in a larger patient cohort, we analyzed bulk RNA-seq data from MASLD samples in SteatoSITE^2^. Hepatic *OLR1* expression correlated positively with histological fibrosis stage (Fig. 4g) and other fibroinflammatory genes (Fig. 4h), and negatively with genes expressed by homeostatic nonparenchymal cell populations such as KCs (*TIMD4*, *MARCO*) or liver sinusoidal endothelial cells (*CLEC4M*, *CLEC4G*; Fig. 4h). To confirm SAMac2 expansion across fibrosis stages, we performed deconvolution analysis of SteatoSITE bulk RNA-seq data using MP scRNA-seq data. SAMac2 and SAMac1 proportions both increased with fibrosis stage in MASLD (Fig. 4i,j). We confirmed these findings in bulk liver RNA-seq data from additional histologically staged CLD cohorts. *OLR1* upregulation and SAMac2 expansion were associated with increasing fibrosis stage in additional patients with MASLD^37^ (Extended Data Fig. 6d,e), PSC^38^ (Extended Data Fig. 6f,g), ARLD^39^ (Extended Data Fig. 6h,i) and PBC^40^ (Extended Data Fig. 6j,k).

The SteatoSITE cohort includes linked longitudinal clinical outcome data^2^. Strikingly, high hepatic *OLR1* expression and expansion of the SAMac2 population were both associated with higher overall mortality (Fig. 4k,l) and increased rates of liver disease progression (Fig. 4m,n) in MASLD. Overall, these data indicate that OLR1^+^ SAMacs may promote hepatic inflammation and fibrogenesis across the full spectrum of severity in different CLD etiologies.

### OLR1^+^ SAMacs are conserved across species

**Fig. 5:**
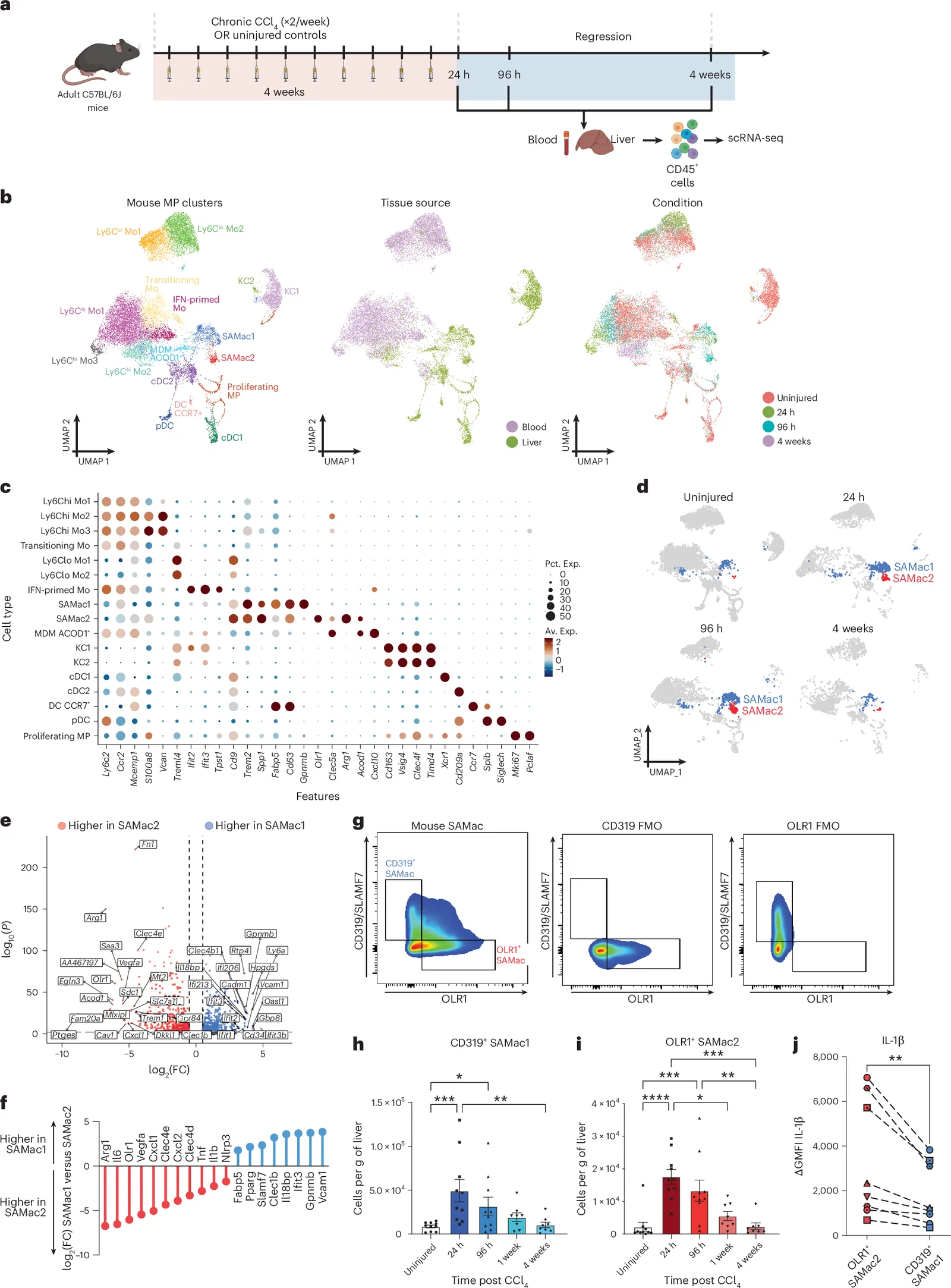
Pro-inflammatory OLR1^+^ SAMacs accumulate in mouse CLD. **a**, Overview of experimental workflow for mouse scRNA-seq data generation. Schematic in **a** created in BioRender; Colella, F. https://BioRender.com/su1qjok (2025). **b**, UMAPs of mouse MP scRNA-seq data (18,177 cells). Colored and labeled by MP cell-type annotation (left); colored by tissue source blood versus liver (middle); colored by condition/CCl4 model time point (right). **c**, Dot plot showing mean expression of marker genes and percentage of cells expressing them for each mouse MP cell type. **d**, UMAPs of mouse MP scRNA-seq data split by stated condition. SAMac1 is labeled in blue and SAMac2 is labeled in red. Other MP cell types are labeled in gray. **e**, Volcano plot of differential gene expression between SAMac1 and SAMac2 in the mouse MP atlas. Two-sided MAST hurdle model. *P* values are adjusted for multiple testing using Bonferroni correction. Significant genes (log_2_(FC) > 0.5 and *P*_adj_ < 0.01) are colored in blue if enriched in SAMac1 and red if enriched in SAMac2. Selected genes are labeled. **f**, log_2_(FC) plot of selected significant genes from **e**. Circles in red are genes enriched in SAMac2 and circles in blue are genes enriched in SAMac1. **g**, Representative flow cytometry plots showing gating strategy for identification of OLR1^+^ SAMac2 and CD319^+^ SAMac1 in mouse liver. Samples pregated on viable mouse liver SAMacs (Extended Data Fig. 8a). Full stained, CD319 FMO and OLR1 FMO controls are shown. **h**, Bar plot of CD319^+^ SAMac numbers (quantitated using flow cytometry) in livers of uninjured (control) mice and in mice at stated time point after 4 weeks of chronic CCl_4_ administration. **i**, Bar plot of OLR1^+^ SAMac numbers (quantitated using flow cytometry) in livers of uninjured (control) mice and in mice at stated time point after 4 weeks of chronic CCl_4_ administration. For **h** and **i**, bars indicate mean ± s.e.m. with individual dots representing independent mice (*n* = 10 uninjured, *n* = 10 24 h, *n* = 10 96 h, *n* = 8 1 week, *n* = 8 4 weeks). KW test with Dunn’s multiple comparison; *P < 0.05, **P < 0.01, ***P < 0.001, ****P < 0.0001. **j**, IL-1β production in OLR1^+^ SAMac2 and CD319^+^ SAMac1 isolated from chronic CCl_4_ treated mouse livers (*n* = 8) was assessed by intracellular flow cytometry. Symbols represent independent mice, with SAMac2 colored in red and SAMac1 colored in blue for each mouse. To measure active IL-1β production, Δ geometric mean fluorescence intensity (ΔGMFI) of IL-1β was calculated for SAMac1 and SAMac2 for each sample, by subtracting the IL-1β GMFI of control cells (incubated with momensin at 4 °C for 3 h) from the IL-1β GMFI after incubation with momensin at 37 °C for 3 h. Two-sided Wilcoxon matched-pairs signed-rank test; **P < 0.001 (*P* = 0.0078). Mo, monocyte; FMO, fluorescence minus one. Source data

**Extended Data Fig. 7:**
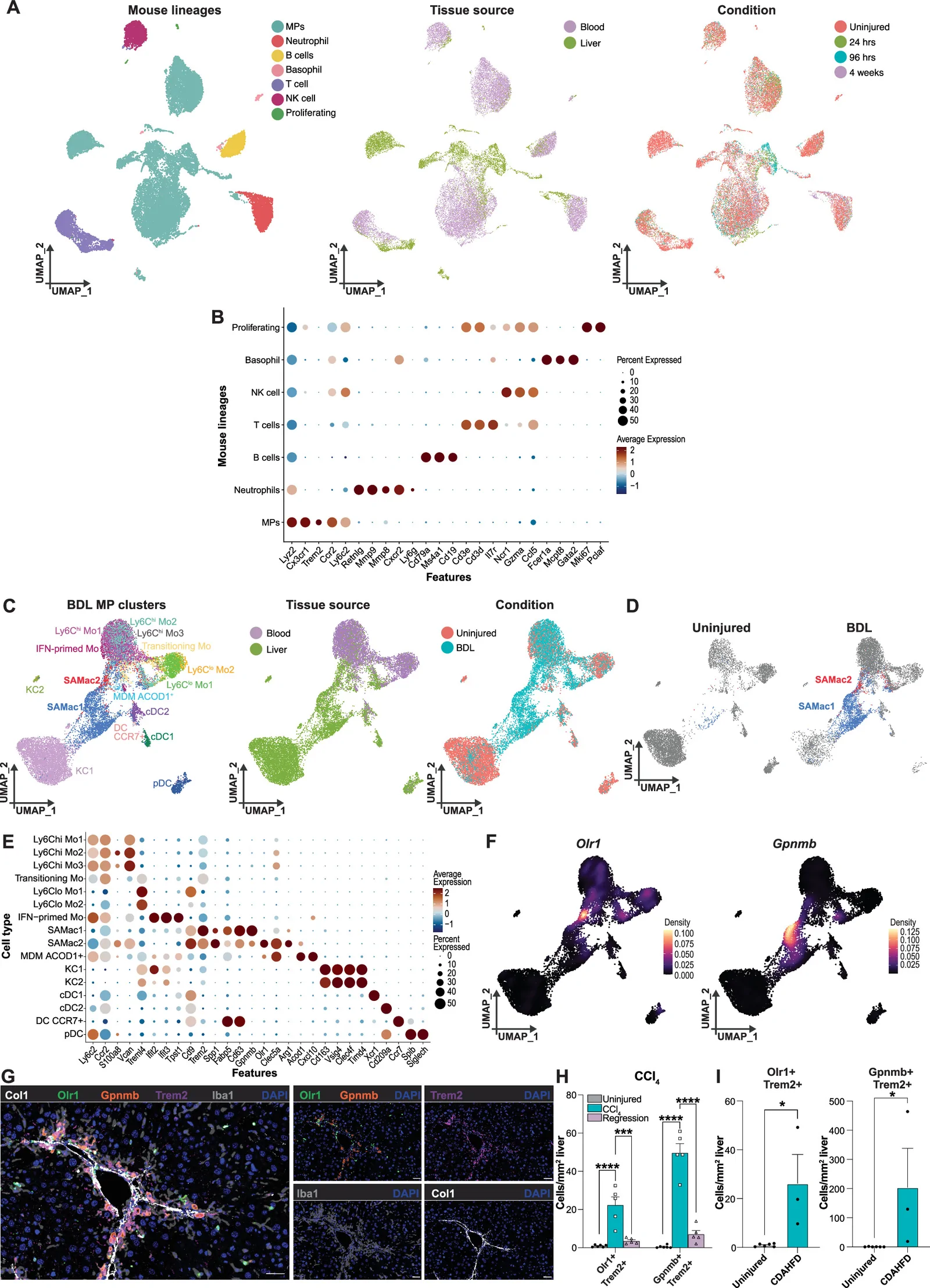
SAMac heterogeneity is conserved in mouse CLD. **a**, UMAPs of mouse immune cell scRNA-seq data (27,070 cells) from blood and liver of chronic carbon tetrachloride (CCl_4_) model (Fig. 4a). UMAPs colored and labeled by cell lineage annotation (left), by tissue source blood versus liver (middle) and by condition/CCl_4_ model time point (right). **b**, Dot plot showing mean expression of marker genes and percentage of cells expressing them for each mouse cell lineage. **c**, UMAPs of mouse bile duct ligation (BDL) MP scRNA-seq data (17,240 cells). Colored and labeled by MP cell type annotation (left). Colored by tissue source blood versus liver (middle). Colored by condition (right). **d**, UMAPs of mouse BDL MP scRNA-seq data split by stated condition. SAMac1 labeled in blue and SAMac2 labeled in red. Other MP cell types labeled in gray. **e**, Dot plot showing mean expression of marker genes and percentage of cells expressing them for each mouse BDL MP cell type. **f**, Density plot showing expression of *Olr1* and *Gpnmb* in mouse BDL MPs. **g**, Representative merged and separate channel multiplex immunofluorescence images of the fibrotic niche in mouse CCl_4_ liver tissue. Markers are collagen 1 (white), Olr1 (green), Gpnmb (orange), Trem2 (purple), Iba1 (gray) and DAPI (blue). Scale bar = 50 μm. **h**, Bar plots of Iba1^+^ Trem2^+^ Olr1^+^ and Iba1^+^ Trem2^+^ Olr1^−^ Gpnmb^+^ SAMac numbers/mm^2^ mouse liver tissue (quantitated using immunofluorescence) in healthy (*n* = 6), chronic CCl_4_ (*n* = 5) and following 4 weeks regression from chronic CCl_4_ (*n* = 5). Bars indicate mean ± s.e.m. Individual dots represent independent mice. One-way ANOVA with Tukey’s multiple comparison; ***P < 0.001, ****P < 0.0001. **i**, Bar plots of Iba1^+^ Trem2^+^ Olr1^+^ and Iba1^+^ Trem2^+^ Olr1^−^ Gpnmb^+^ SAMac numbers/mm^2^ mouse liver tissue (quantitated using immunofluorescence) in healthy (*n* = 6) and MASH (*n* = 3) mouse livers. MASH was induced via 10 weeks of CDAHFD. Bars indicate mean ± s.e.m. with individual dots representing independent mice. Two-tailed Mann–Whitney test; *P < 0.05 (Olr1^+^ Trem2^+^ *P* = 0.0238; Gpnmb^+^ Trem2^+^ *P* = 0.0238). Source data

To assess cross-species conservation, we used the mouse carbon tetrachloride (CCl_4_) mouse model of liver fibrosis progression and regression^17^, performing scRNA-seq on blood and liver CD45^+^ immune cells from uninjured, liver fibrosis (24 and 96 h) and fibrosis regression (4 weeks) groups (Fig. 5a). After QC and lineage annotation (Extended Data Fig. 7a,b), 18,177 MPs partitioned grouped into 17 cell clusters, revealing both the expected subpopulations of monocytes, macrophages and DCs and previously unreported mouse MP subpopulations, such as an *Acod1*^+^ *Cxcl10*^+^ liver macrophage cluster (MDM Acod1^+^; Fig. 5b,c and Supplementary Table 13). We identified two subpopulations of *Trem2*^+^ *Cd9*^+^ *Cd63*^+^ *Spp1*^+^ SAMacs, termed SAMac1 and SAMac2 (Fig. 5b,c). Both mouse SAMac subpopulations were liver derived (Fig. 5b) and expanded at fibrotic time points (24 and 96 h), but had returned to baseline following regression (4 weeks; Fig. 5d). As with human SAMacs, differential expression between mouse SAMac1 and SAMac2 revealed transcriptional heterogeneity (Fig. 5e and Supplementary Table 14), with SAMac1 being enriched for *Gpnmb*, *Slamf7* and other anti-inflammatory mediators (for example, *Il18bp* and *Pparg*), while mouse SAMac2 showed elevated expression of *Olr1* and pro-inflammatory factors including inflammasome components *Il1b* and *Nlrp3* (Fig. 5f). We confirmed our findings using scRNA-seq of blood and liver CD45^+^ cells from bile duct ligation (BDL) mouse model, demonstrating expansion of SAMac1 and SAMac2 in BDL livers (Extended Data Fig. 7c,d) and highlighting a similar MP marker gene profile across models (Extended Data Fig. 7e), including the utility of *Olr1* and *Gpnmb* as markers to distinguish SAMac2 and SAMac1, respectively (Extended Data Fig. 7e,f). Mouse CCl_4_ MP data can be browsed at https://shiny.igc.ed.ac.uk/mouseMPshinyApp/.

**Extended Data Fig. 8:**
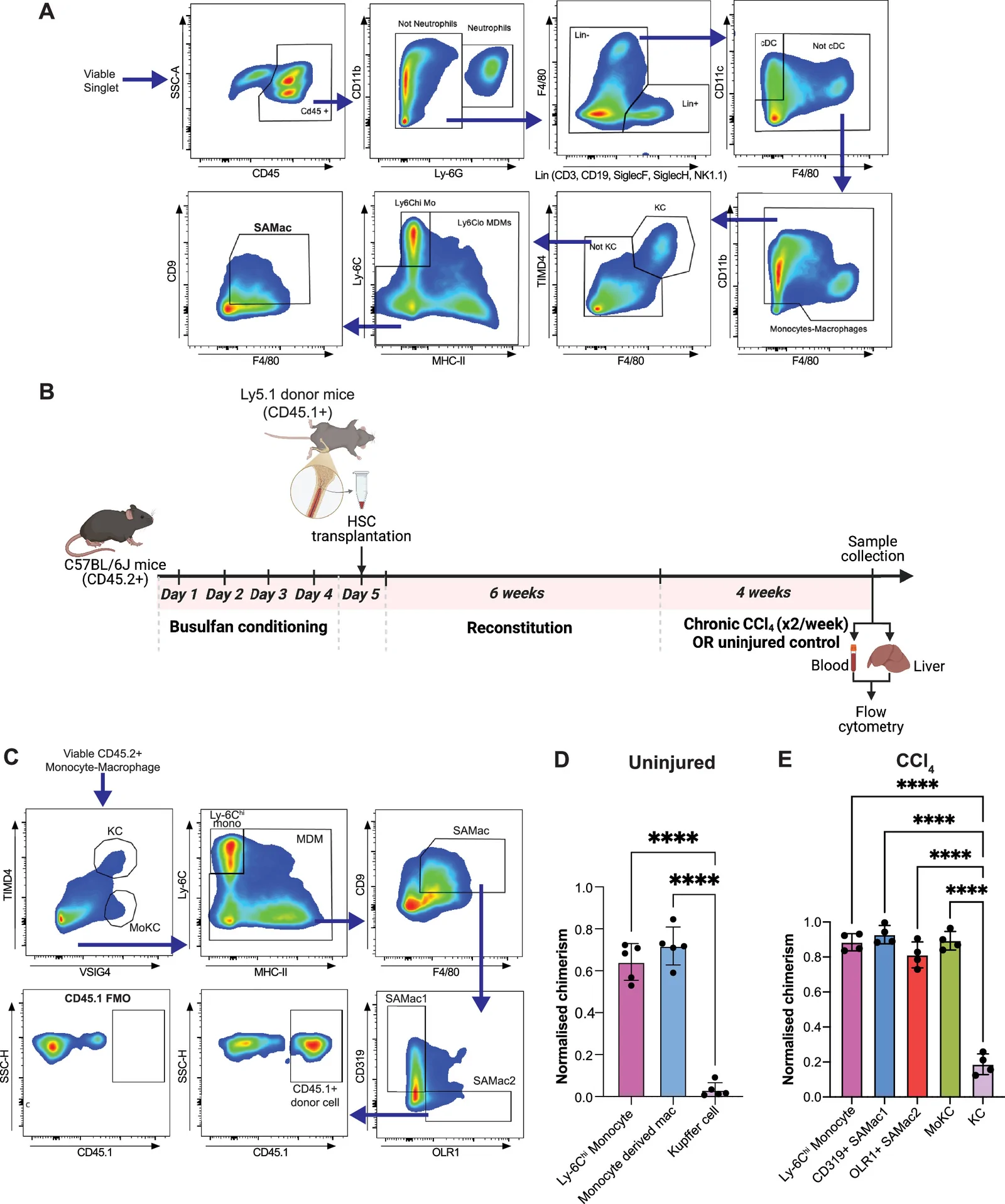
Dissecting SAMac origin and kinetics in mouse CLD. **a**, Flow cytometry gating strategy for study of SAMac kinetics in mouse liver. Cells were pregated as viable (DAPI negative) singlets (based on FSC-A versus FSC-H and SSC-A versus SSC-H). Identified SAMac population is then gated for OLR1 and CD319 expression as shown Fig. 5g. **b**, Overview of experimental workflow for mouse bone marrow chimera CLD model. Wild-type mice (CD45.2^+^) were used as recipients and CD45.1^+^ mice as bone marrow donors. Schematic in **b** created in BioRender; Colella, F. https://BioRender.com/fnfo96e (2025). HSC, hematopoietic stem cell. **c**, Flow cytometry gating strategy for bone marrow chimera studies to quantitate macrophage subpopulation chimerism in mouse liver. Cells were pregated as viable (DAPI negative) singlets (based on FSC-A versus FSC-H and SSC-A versus SSC-H) and monocyte-macrophages defined as shown in **a**. The gated macrophage subpopulations shown were then all assessed for the degree of chimerism (% CD45.1^+^). MDM, monocyte-derived macrophage; KC, Kupffer cell; moKC, monocyte-derived Kupffer cell. **d**, Bar plot of degree of chimerism of liver macrophage subpopulations, quantitated by flow cytometry of uninjured mice (*n* = 5) following the busulfan chimera protocol. Bars indicate mean ± s.d. with individual dots representing independent mice. Normalized chimerism calculated for each mouse by normalizing to the % of peripheral blood monocytes which are donor-derived (CD45.1^+^). Ordinary one-way ANOVA with Tukey’s multiple comparisons test; ****P < 0.0001. **e**, Bar plot of degree of chimerism of liver macrophage subpopulations, quantitated by flow cytometry of chronic CCl_4_ treated mice (*n* = 4) following the busulfan chimera protocol. Bars indicate mean ± s.d. with individual dots representing independent mice. Normalized chimerism calculated for each mouse by normalizing to the % of peripheral blood monocytes that are donor-derived (CD45.1^+^). Ordinary one-way ANOVA with Tukey’s multiple comparisons test. ****P < 0.0001. Source data

To study mouse SAMac1 and SAMac2 kinetics, we conducted flow cytometry to distinguish CD319/Slamf7^+^ SAMac1 and Olr1^+^ SAMac2 in the CCl_4_ model (Extended Data Fig. 8a and Fig. 5g). Both Olr1^+^ SAMacs and CD319^+^ SAMacs expanded during active liver injury but returned to baseline 4 weeks after CCl_4_ cessation (Fig. 5h,i). Flow cytometry also showed that Olr1^+^ SAMacs had increased production of IL-1β compared to CD319^+^ SAMacs (Fig. 5j), mirroring transcriptional findings.

Multiplex immunofluorescence distinguished Iba1^+^ Trem2^+^ Olr1^+^ SAMac2 and Iba1^+^ Trem2^+^ Olr1^−^ Gpnmb^+^ SAMac1 in situ (Extended Data Fig. 7g). Both Olr1^+^ and Gpnmb^+^ SAMacs were located adjacent to the collagen 1 positive fibrotic niche (Extended Data Fig. 7g) and accumulated in the CCl_4_ and CDAHFD models of CLD (Extended Data Fig. 7h,i). However, after the cessation of liver injury in the CCl_4_ model, both SAMac subpopulations declined toward baseline (Extended Data Fig. 7h), indicating a temporal and spatial association with fibrogenic activity.

Previous studies have demonstrated that SAMacs derive from circulating monocytes rather than resident KCs^13,33,41^. To confirm the origin of SAMac subpopulations, we generated busulfan-conditioned bone-marrow chimeric mice^42^, using CD45.1^+^ expression to track bone-marrow-derived donor cells (Supplementary Note 2 and Extended Data Fig. 8b–e). These data demonstrate that both SAMac subpopulations are derived from the recruitment and differentiation of circulating monocytes.

### OLR1^+^ SAMacs are enriched for distinct transcription factor (TF) motifs

**Fig. 6:**
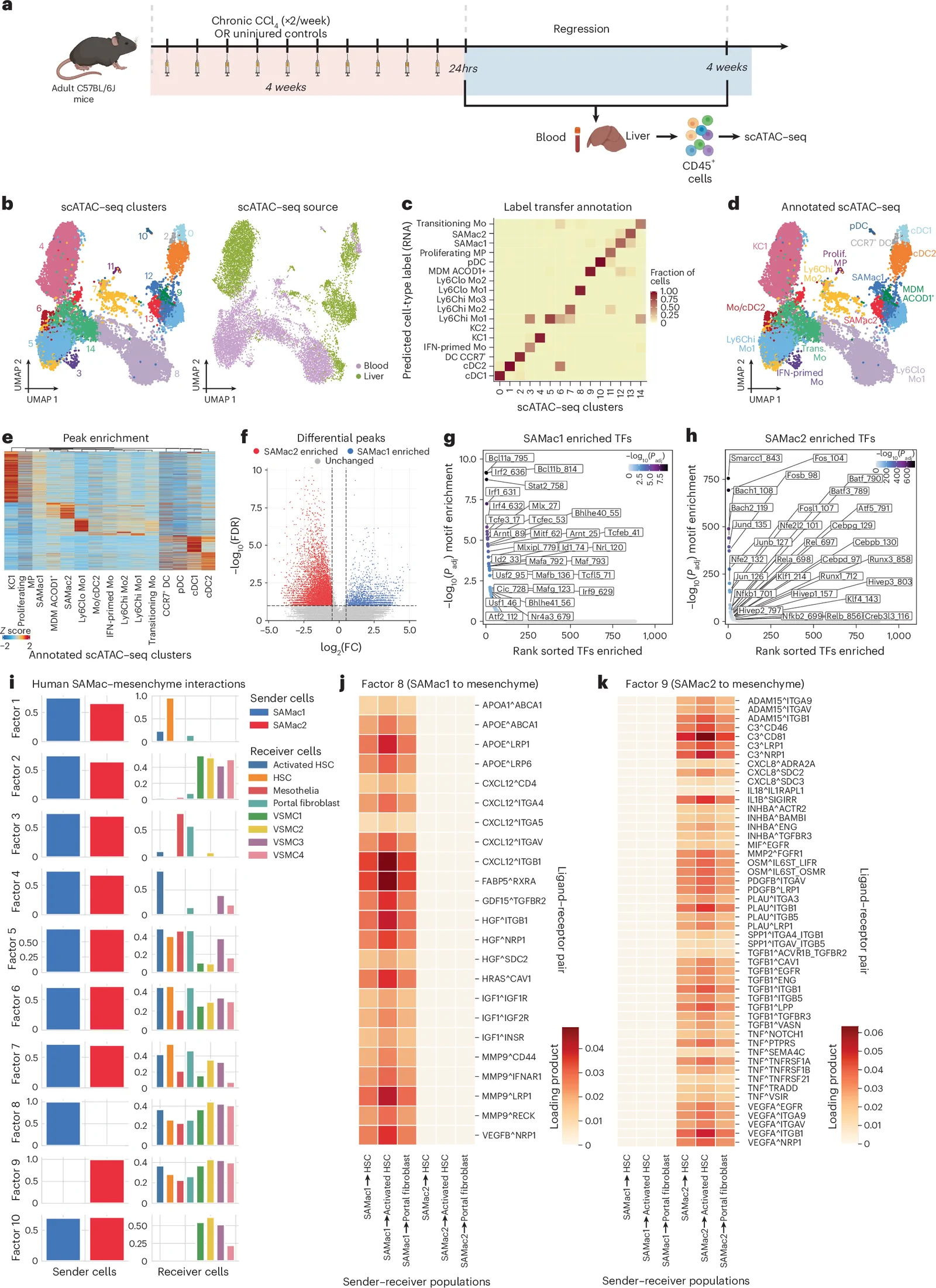
OLR1^+^ SAMacs are enriched for pro-inflammatory TF motifs and cell–cell interactions with mesenchymal cells. **a**, Overview of experimental workflow for mouse scATAC–seq data generation. Schematic created in BioRender; Colella, F. https://BioRender.com/xzgt2un (2025). **b**, UMAPs of mouse MP scATAC–seq data (14,613 cells). Colored and labeled by scATAC–seq clusters (left) and by tissue source blood versus liver (right). **c**, Heatmap of label transfer annotation analysis between mouse MP scATAC–seq clusters and annotated mouse MP scRNA-seq clusters (RNA). Color scale indicates prediction score of annotation for each scATAC–seq cluster. The highest prediction scores were used to annotate scATAC–seq clusters. **d**, UMAP of mouse MP scATAC–seq data is colored and labeled by cell-type annotation. **e**, Heatmap representation of marker chromatin accessibility peak enrichment in each annotated MP cell type from the scATAC–seq peak set. Each row represents a peak, and each column represents an annotated cluster in the scATAC–seq dataset. Color scale indicates *z*-score. **f**, Volcano plot of differential peak enrichment between SAMac1 and SAMac2 in the mouse scATAC–seq MP data. Significant peaks (log_2_(FC) > 0.5 and FDR < 0.1) are colored in blue if enriched in SAMac1 and red if enriched in SAMac2. **g**, Rank significance plot of TF motifs enriched in SAMac1 versus SAMac2 from scATAC–seq data. Selected TF motifs are labeled. TF motifs are colored by significance. **h**, Rank significance plot of TF motifs enriched in SAMac2 versus SAMac1 from scATAC–seq data. Selected TF motifs are labeled. TF motifs are colored by significance. For **g** and **h**, TF motif enrichment was assessed using one-sided hypergeometric test; *P* values were adjusted for multiple testing using the BH FDR; motifs with FDR ≤ 0.1 were considered significant. **i**, Ligand–receptor interaction analysis in human level 3 atlas, between SAMac1 and SAMac2 (as senders) and mesenchymal cell clusters (as receivers). Ten factors were identified representing different cell–cell communication programs. Loadings (*y* axis) for different sender and receiver cell types are shown for each factor. **j**, Heatmap of selected ligand–receptor pairs in factor 8. Rows represent ligand–receptor pairs; columns represent specific cell-type interactions; color scale represents the loading product (degree of enrichment). **k**, Heatmap of selected ligand–receptor pairs in factor 9. Rows represent ligand–receptor pairs; columns represent specific cell-type interactions; color scale represents the loading product (degree of enrichment).

**Extended Data Fig. 9:**
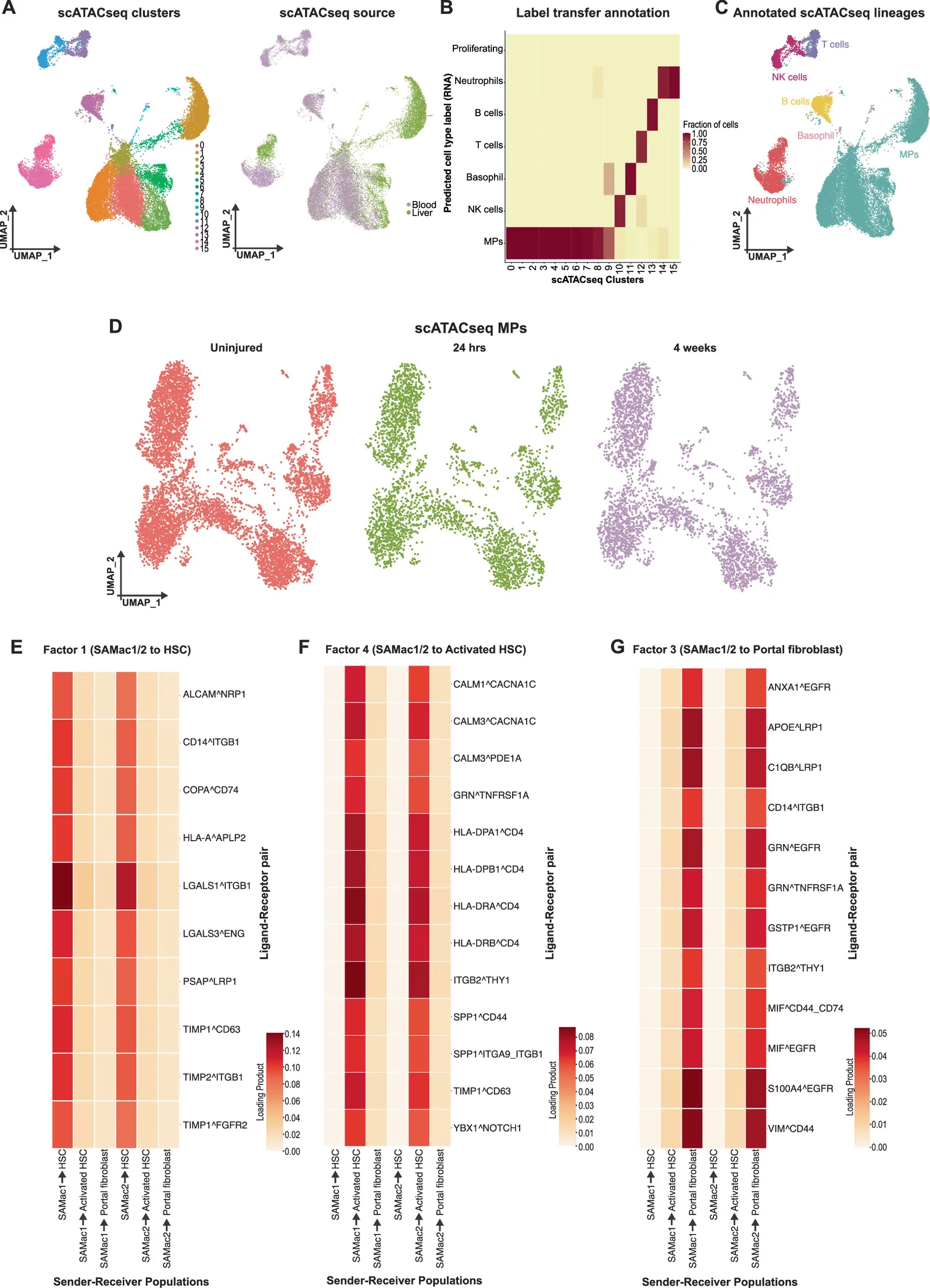
Identifying mouse MPs for subsequent scATAC–seq analysis. **a**, UMAPs of mouse immune cell single-cell ATAC-seq (scATAC–seq) data (27,272 cells) from blood and liver of chronic CCl_4_ model (Fig. 6a). Colored and labeled by immune cell scATAC–seq clusters (left) and by tissue source blood versus liver (right). **b**, Heatmap of label transfer annotation analysis between mouse immune cell scATAC–seq clusters and annotated mouse immune cell scRNA-seq clusters (from Extended Data Fig. 7a). Color scale indicates prediction score of annotation of each scATAC–seq cluster. The highest prediction scores were used to annotate immune cell scATAC–seq clusters. **c**, UMAP of mouse immune cell scATAC–seq data colored and labeled by cell type annotation. MPs were then subsetted for further analysis. **d**, UMAPs of mouse MP scATAC–seq data (Fig. 5b), colored and split by condition/CCl_4_ model time point. **e**–**g**, Supplementary heatmaps of ligand–receptor interaction analysis in human level 3 atlas shown in Fig. 5i. Each factor represents a different cell–cell communication program. **e**, Heatmap of selected ligand–receptor pairs in factor 1. Rows represent ligand–receptor pairs; columns represent specific cell type interactions; color scale represents the loading product (degree of enrichment). **f**, Heatmap of selected ligand–receptor pairs in factor 4. Rows represent ligand–receptor pairs; columns represent specific cell type interactions; color scale represents the loading product (degree of enrichment). **g**, Heatmap of selected ligand–receptor pairs in factor 3. Rows represent ligand–receptor pairs; columns represent specific cell type interactions; color scale represents the loading product (degree of enrichment).

To assess the transcriptional regulation of SAMac subpopulations, we performed single-cell assay for transposase-accessible chromatin using sequencing (scATAC–seq) on blood and liver CD45^+^ cells from the CCl_4_ CLD mouse model (Fig. 6a). After the identification of MPs (Supplementary Note 3 and Extended Data Fig. 9a–c), they partitioned into 15 scATAC–seq MP clusters (Fig. 6b and Extended Data Fig. 9d). Clusters were annotated through label transfer from mouse scRNA-seq data, distinguishing SAMac1 and SAMac2 (Fig. 6c,d). Mouse MP subpopulations demonstrated distinct chromatin accessibility profiles (Fig. 6e), with significant differences observed between SAMac1 and SAMac2 (Fig. 6f). TF motif analysis on SAMac1-enriched peaks revealed enrichment for *Bcl11A* and *Bcl11B* motifs, as well as several interferon-responsive TFs including *Irf1*, *Irf2*, *Irf4* and *Stat2* (Fig. 6g and Supplementary Table 15). In contrast, Olr1^+^ SAMac2 showed enrichment for several pro-inflammatory TF motifs including *Fos*, *Fosb*, *Jun*, *NfkB* and family members *Rel* and *Rela* (Fig. 6h and Supplementary Table 15). SAMac2 was also enriched for several transcriptional repressors such as *Nfe2*, *Nfe2l2* and *Bach2*, suggesting a balance between activation and regulatory signals (Fig. 6h). Hence, Olr1^+^ SAMac2 has an open chromatin profile primed for inflammatory responses in the injured liver.

### OLR1^+^ SAMacs show pro-inflammatory interactions with mesenchyme

To understand how SAMac subpopulations regulate fibrosis, we used the LIANA^+^ package^43^ to perform interactome analysis between the two human SAMac subpopulations (as senders) and mesenchymal cell clusters (as receivers). Focusing on HSCs, activated HSCs and portal fibroblasts as the main mesenchymal cells that express extracellular matrix and regulate liver fibrosis, we identified three groups (factors) of ligand–receptor pairs where ligands were expressed by both SAMac1 and SAMac2 (Fig. 6i and Supplementary Table 16). Common SAMac-derived ligands included galectins and TIMPs between SAMac1/SAMac2 and HSCs (Extended Data Fig. 9e), calmodulins and osteopontin (*SPP1*) between SAMac1/SAMac2 and activated HSCs (Extended Data Fig. 9f), and proganulin (*GRN*) and macrophage migration inhibitory factor between SAMac1/SAMac2 and portal fibroblasts (Extended Data Fig. 9g). We also identified two factors that were specific to SAMac1 and SAMac2 as senders, factor 8 and factor 9, respectively (Fig. 6i and Supplementary Table 16). Factor 8 (SAMac1) ligands contained several reported antifibrotic factors, including *HGF*^44^, *IGF1* (ref. ^45^), *MMP9* (ref. ^46^) and *GDF15* (ref. ^47^; Fig. 6j). In contrast, factor 9 (SAMac2) ligands included *TNF*^48^, *TGFB1*, *IL1B*^49–51^, *IL18*, *C3* and *PDGFB* (Fig. 6k), mediators that have been associated with promoting fibrogenesis in the liver and/or other organs. Hence, SAMac heterogeneity has potential consequences for signaling to mesenchymal cells, with OLR1^+^ SAMac2 predicted to promote activation of scar-producing activated HSCs and portal fibroblasts.

To determine whether OLR1^+^ monocyte-derived macrophages (MDMs) have a functional role in liver fibrosis, we used a human multilineage spheroid culture system^52–54^, supplemented with the addition of primary human monocytes from patients with CLD. This model demonstrated that human OLR1^+^ MDMs have a pro-fibrotic function (Supplementary Note 4 and Supplementary Fig. 3a–e).

**Fig. 7:**
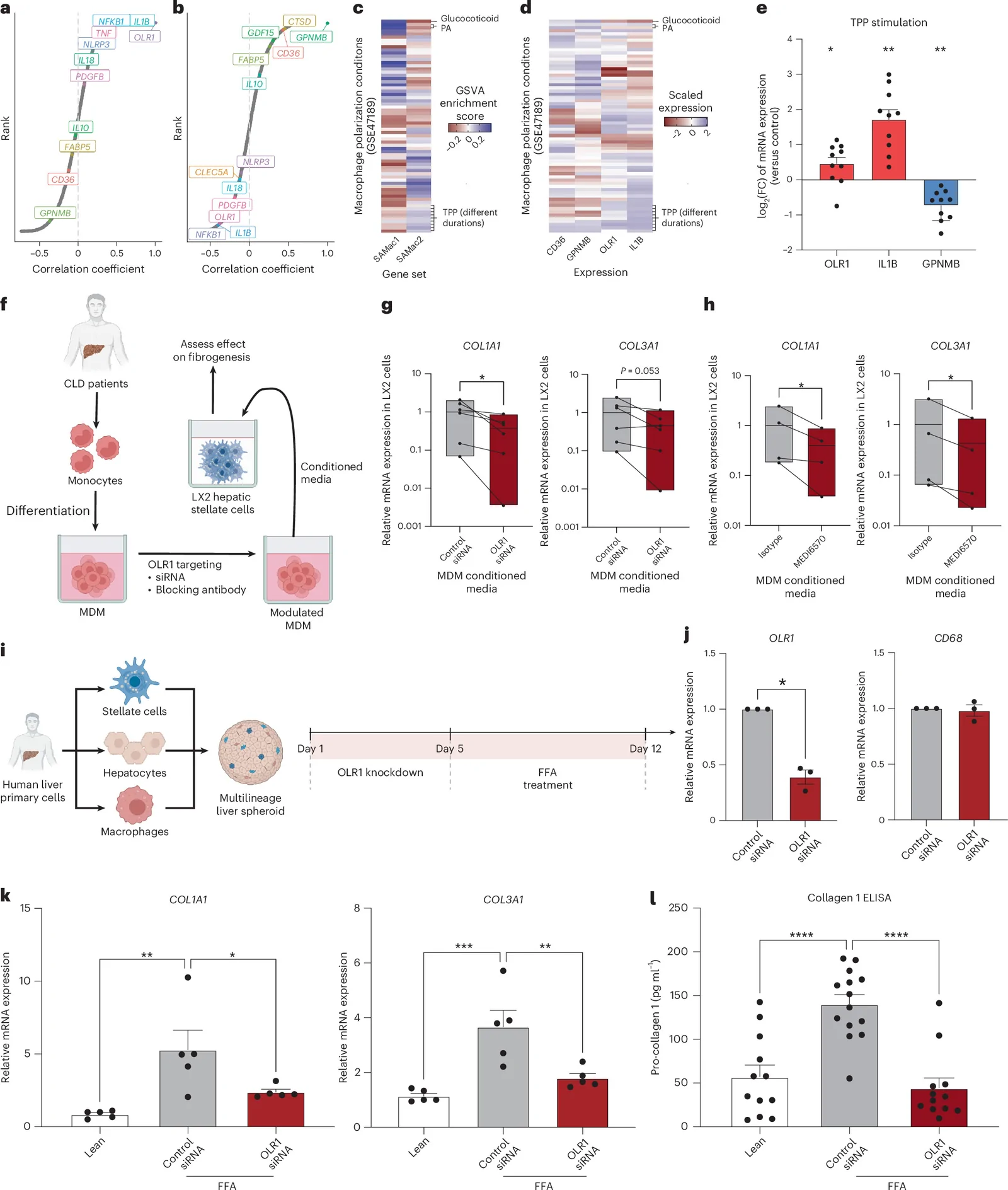
Targeting OLR1^+^ MDMs attenuates fibrogenic activity. **a**, Genes coexpressed with *OLR1* across 67 monocyte/macrophage activation conditions, ranked by Pearson correlation coefficient. Selected genes are highlighted. **b**, Genes coexpressed with *GPNMB* across 67 monocyte/macrophage activation conditions, ranked by Pearson correlation coefficient. Selected genes are highlighted. **c**, GSVA enrichment scores for 67 different monocyte/macrophage activation conditions to identify stimuli that phenocopy SAMac1 or SAMac2 gene sets derived from liver scRNA-seq data. Selected activation stimuli are highlighted. **d**, Scaled gene expression for selected genes across 67 different monocyte/macrophage activation conditions. Selected activation stimuli are highlighted. **e**, qPCR of primary human MDMs (*n* = 10) showing effects of TPP stimulation on expression of selected genes shown as log_2_(FC) relative to expression in control samples. Dots represent separate patients. Bars indicate mean ± s.e.m. Two-sided Wilcoxon matched-pairs signed-rank test for each gene versus control; *P < 0.05, **P < 0.01 (*OLR1* *P* = 0.0371; *IL1B* *P* = 0.0020; *GPNMB* *P* = 0.0020). **f**, Overview of experimental workflow for assessing effect of MDM OLR1 modulation on HSC fibrogenic activity. **g**, Relative *COL1A1* and *COL3A1* mRNA expression of LX2 HSCs after incubation with conditioned media from primary MDMs (*n* = 6 patients) pretreated with OLR1 targeting or control siRNA. Boxes are min-to-max expression with line at mean. Dots connected by lines represent data from MDMs of six different patients with CLD. Two-sided ratio paired *t* test, *P < 0.05 (*P* = 0.0291 for *COL1A1*; *P* = 0.0532 for *COL3A1*). **h**, Relative *COL1A1* and *COL3A1* mRNA expression of LX2 HSCs after incubation with conditioned media from primary MDMs pretreated with OLR1 blocking antibody (MEDI6570) or isotype control antibody. Boxes are min-to-max expression with line at mean. Dots connected by lines represent data from MDMs of four different patients with CLD. Two-sided ratio paired *t* test, *P < 0.05 (*P* = 0.0496 for *COL1A1*; *P* = 0.0218 for *COL3A1*). **i**, Overview of experimental workflow for assessing effects of OLR1 modulation on fibrogenic activity in primary human 3D multilineage spheroids after MASH induction by FFA. Schematics in **f** and **i** created in BioRender: **f**, Colella, F. https://BioRender.com/l42u16a (2025); **i**, Colella, F. https://BioRender.com/pjoyobq (2026). **j**, qPCR of MASH spheroids (*n* = 3) treated with OLR1 or control siRNA. Dots represent different spheroid cultures. Expression of stated gene in each OLR1 siRNA-treated spheroid culture relative to control siRNA-treated spheroid. Bars indicate mean ± s.e.m. Two-sided Welsh’s *t* test; *P < 0.05 (*P* = 0.0106). **k**, qPCR of *COL1A1* and *COL3A1* in lean spheroids (no FFA treatment) and MASH primary liver spheroids treated with OLR1 or control siRNA. Dots represent different spheroid cultures, *n* = 5 per group. Bars indicate mean ± s.e.m. One-way ANOVA with Dunnett’s multiple comparison test; *P < 0.05, **P < 0.01, ***P < 0.001. **l**, ELISA for pro-collagen 1 from lean spheroids (*n* = 12) and MASH primary liver spheroids treated with OLR1 (*n* = 12) or control (*n* = 14) siRNA. Dots represent different spheroid cultures. One-way ANOVA with Dunnett’s multiple comparison test; ****P < 0.0001. ANOVA, analysis of variance; GSVA, gene set variation analysis. Source data

**Extended Data Fig. 10:**
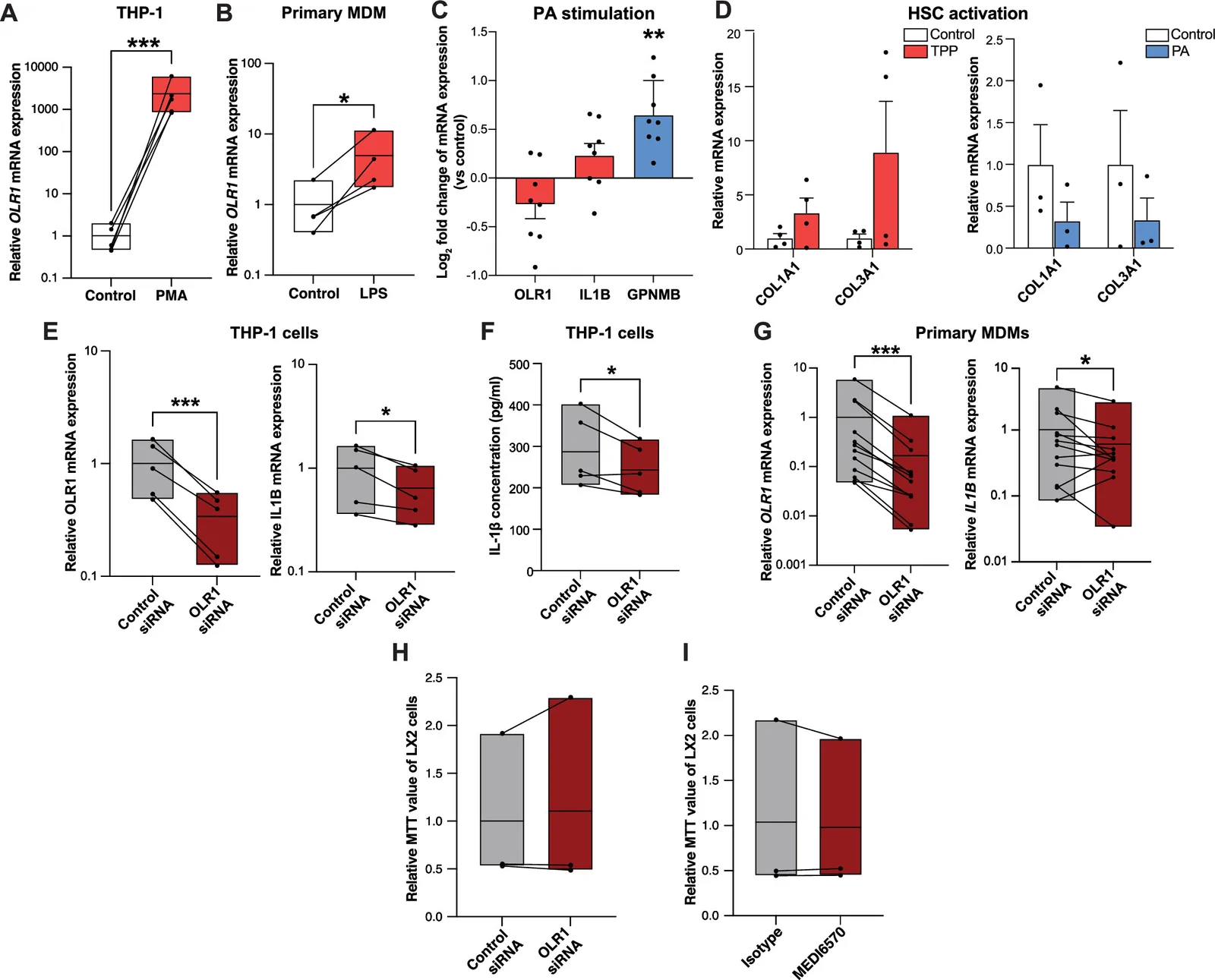
Modulation of macrophage OLR1 in vitro. **a**, Relative *OLR1* mRNA expression of THP-1 cells treated with PMA (phorbol 12-myristate-13-acetate) or vehicle. Dots connected by lines represent 5 independent experiments. **b**, Relative *OLR1* mRNA expression of primary MDM following treatment with lipopolysaccharide (LPS) or vehicle control. Dots connected by lines represent 4 CLD patients. For **a** and **b**, bars are min-to-max expression with line at mean. Two-sided ratio paired t-test, ***P < 0.001 (*P* = 0.002), *P < 0.05 (*P* = 0.0168). **c**, qPCR of primary human MDMs (*n* = 8) comparing palmitic acid (PA) versus control. Expression of selected genes shown as log_2_ fold change relative to expression in controls. Dots represent separate patients. Bars indicate mean ± s.e.m. Two-sided Wilcoxon matched-pairs signed rank test for each gene versus control; **P < 0.01 (*P* = 0.0078). **d**, Relative *COL1A1* and *COL3A1* mRNA expression in LX2 hepatic stellate cells following incubation with conditioned media from MDMs pretreated with TPP (*n* = 4) or PA (*n* = 3) versus respective control. Bars indicate mean ± s.e.m.; dots represent individual donors. **e**, Relative *OLR1* and *IL1B* mRNA expression in THP-1 cells following treatment with OLR1 targeting or control siRNA. Dots connected by lines represent 5 independent experiments. Two-sided ratio paired t-test, *P < 0.05 (*P* = 0.0135), ***P < 0.001 (*P* = 0.004). **f**, IL-1β concentration measured by ELISA in the supernatant of differentiated THP-1 cells following treatment with OLR1 targeting or control siRNA. Dots connected by lines represent 5 independent experiments. Two-sided ratio paired t-test, *P < 0.05 (*P* = 0.0349). **g**, Relative *OLR1* and *IL1B* mRNA expression of primary MDM following treatment with OLR1 targeting or control siRNA. Dots connected by lines represent 12 different CLD patients. Two-sided Wilcoxon matched-pairs signed rank test, *P < 0.05 (*P* = 0.0425), ***P < 0.001 (*P* = 0.0005). **h**, Relative MTT assay value of LX2 hepatic stellate cells following incubation with conditioned media from primary MDMs pretreated with OLR1 targeting or control siRNA. Dots connected by lines represent data from MDMs of 3 different CLD patients. **i**, Relative MTT assay value of LX2 hepatic stellate cells following incubation with conditioned media from primary MDMs pretreated with OLR1 blocking antibody (MEDI6570) or isotype control antibody. Dots connected by lines represent data from MDMs of 3 different CLD patients. For **e**–**i**, bars are min to max expression with line at mean. Source data

We then sought to assess drivers of *OLR1* expression and SAMac differentiation. *OLR1* expression was upregulated in THP-1 MDMs in response to phorbol 12-myristate 13-acetate (Extended Data Fig. 10a), and in primary human MDMs in response to lipopolysaccharide (Extended Data Fig. 10b). To understand conditions promoting SAMac1 and SAMac2 differentiation, we interrogated a transcriptomic dataset where human MDMs were polarized using 67 different conditions^55^. *OLR1* expression correlated positively with pro-inflammatory genes (*IL1B*, *TNF*, *IL18*, *NLRP3*, *NFKB1*) across conditions (Fig. 7a), while *GPNMB* expression correlated positively with genes such as *CD36* and *IL10* and negatively with pro-inflammatory genes (Fig. 7b). Across stimuli, the TPP model (stimulation with TNF, prostaglandin E2 and Pam3CSK4) consistently enriched expression of a human liver SAMac2 gene set (Fig. 7c) and transcription of *OLR1* and *IL1B* (Fig. 7d). The TPP model mimics chronic inflammation^55^ and more closely resembles human macrophages from chronic inflammatory diseases^56^. In contrast, in the SAMac1 gene set, *GPNMB* and *CD36* expression were enriched by fatty acids (for example, palmitic acid (PA)) or anti-inflammatory stimuli such as glucocorticoids (Fig. 7c,d). We confirmed these findings in cirrhotic patient MDMs, demonstrating upregulation of *OLR1* and *IL1B* and downregulation of *GPNMB* in response to TPP (Fig. 7e), while *GPNMB* was upregulated in response to PA (Extended Data Fig. 10c). TPP-polarized MDMs appeared to enhance fibrogenic activity in LX2 HSCs but PA-stimulated MDMs had no impact (Extended Data Fig. 10d). Hence, SAMac2 and SAMac1 represent distinct macrophage polarization states and OLR1 expression correlates with pro-inflammatory pro-fibrotic activation.

We proceeded to investigate whether targeting OLR1 itself could modulate pro-fibrotic macrophage functions. OLR1 knockdown using siRNA in THP-1 MDMs reduced *IL1B* gene expression (Extended Data Fig. 10e) and attenuated IL-1β protein secretion (Extended Data Fig. 10f). Similarly, treatment of primary human MDMs from patients with CLD with OLR1 siRNA decreased *OLR1* and *IL1B* gene expression (Extended Data Fig. 10g). Conditioned media from primary MDMs with OLR1 knockdown was less able to induce fibrogenic activity in LX2 HSCs than control media (Fig. 7f,g). Furthermore, treatment of primary human MDMs with MEDI6570, a clinically tested OLR1 blocking antibody^36^, attenuated fibrillar collagen expression in LX2 cells after conditioned media transfer (Fig. 7h). No effect was observed on LX2 survival with MDM OLR1 knockdown or antibody blockade (Extended Data Fig. 10h,i).

To determine the impact of OLR1 modulation in a more complex primary human CLD model, we used a 3D-MASH model comprising multilineage liver spheroids containing primary hepatocytes, macrophages and HSCs with the addition of lipotoxic free fatty acids (FFA^57^; Fig. 7i). OLR1 knockdown in these spheroids reduced *OLR1* expression (Fig. 7j), attenuated fibrogenic activity (Fig. 7k) and abrogated fibrillar collagen protein production in response to FFA treatment (Fig. 7l). Hence, OLR1 inhibition is a potential new therapeutic approach to selectively modulate SAMac phenotype and inhibit liver fibrogenesis.

## Discussion

Our human liver atlas, comprising 649,295 cells from 77 patients, is a valuable resource to dissect the pathobiology of liver fibrosis. We focused on the immune compartment, identifying a subpopulation of OLR1^+^ SAMacs that expands across different etiologies of human CLD, is conserved across species and has a pro-inflammatory phenotype. Attenuating macrophage OLR1 expression reduces fibrogenic activity, highlighting OLR1 as a potential therapeutic target for liver inflammation and fibrosis in CLD.

SAMacs have been shown to expand in fibrotic human liver in previous studies, but data on their function in fibrosis are conflicting. Some studies demonstrated a pro-fibrotic role for SAMacs through interactions with HSCs^13,14^. In contrast, others have suggested that TREM2^+^ SAMacs have an antifibrotic function in CLD, promoting scar resolution and the restitution of normal tissue architecture^19–22^. Our human scRNA-seq atlas has identified SAMac heterogeneity that could explain this functional dichotomy. In particular, the OLR1^+^ SAMac2 population displays a more pro-inflammatory and pro-fibrogenic phenotype, suggesting that this may be the optimal target population to inhibit pathogenic SAMac properties.

Targeting OLR1 itself represents a potential approach to selectively modulate SAMac2, reinforced by strong associations between elevated liver OLR1 expression and adverse clinical outcomes in SteatoSITE, and functional proof that attenuating OLR1 signaling reduces pro-fibrogenic macrophage properties. OLR1 has been implicated in the pathogenesis of atherosclerosis^35^, cardiac fibrosis^58^ and cancer^59^. Mechanistically, OLR1 signaling in atherosclerosis can induce pro-inflammatory responses, including activation of the NF-*κ*B pathway and/or NLRP3 inflammasome in endothelial cells^60,61^ and macrophages^62,63^. Indeed, OLR1 blockade has been proposed as an atherosclerosis therapy^35^, with the monoclonal antibody MEDI6570 having been administered to patients in phase 1 (ref. ^36^) and phase 2 clinical trials (ClinicalTrials.gov registration: NCT04610892). Exploration of OLR1 inhibition as a therapy for CLD is warranted, either through repurposing of MEDI6570 or through developing small-molecule OLR1 inhibitors. Further work should also interrogate OLR1 biology in CLD, developing a mechanistic understanding of the antifibrotic effects of OLR1 inhibition, defining activating ligands in the CLD liver and understanding the utility of OLR1 as predictive biomarker (Supplementary Discussion). In addition, other SAMac2-expressed candidate targets should be functionally explored, for example, CLEC5A that has been reported to mediate pro-inflammatory activity in myeloid cells^64^.

Our finding of SAMac heterogeneity raises new questions about SAMac biology. TREM2^+^ GPNMB^+^ SAMac1 is transcriptionally analogous to lipid-associated macrophages defined in mice^21,33,65^ and MetMacs in humans^34^, while TREM2^+^ OLR1^+^ SAMac2 reflects a discrete liver macrophage subpopulation. Here we annotated these macrophages as SAMacs due to their temporal and spatial association with scar deposition. However, TREM2^+^ macrophages expand in other diseases such as acute liver injury^33^ or liver cancer^66^. It remains unclear whether TREM2^+^ macrophage phenotypes differ according to the nature or duration of injury. This will be important to ascertain, as macrophage nomenclature may require refinement according to the disease contexts in which they emerge. Additional data on the functional roles of SAMac1 and SAMac2 in CLD pathogenesis are also crucial. While our data suggest that OLR1^+^ and GPNMB^+^ SAMacs represent distinct macrophage polarization states, targeted manipulation of these subpopulations, potentially through selective in vivo depletion, will be important in determining how they regulate local tissue responses. Macrophages are also capable of switching phenotype according to microenvironmental cues. It is unknown whether macrophages can switch between SAMac2 and SAMac1 phenotypes and which signals might regulate this. Further work using a time-controlled fate mapping system is needed to dissect potential SAMac plasticity. Intriguingly, our spatial data suggest that OLR1^+^ and GPNMB^+^ macrophages accumulate in the same niche, suggesting that local reprogramming rather than differential recruitment may be responsible for distinct macrophage phenotypes. Expanded spatial studies, using larger panels of RNA and/or protein markers, will enable improved annotation of cellular phenotypes and facilitate a more nuanced analysis of interactions between SAMac subpopulations and other pathogenic cell types within this niche.

Beyond macrophages, our atlas has facilitated the distinction of rare cell types and identified other CLD-associated cellular states. These findings can be followed up more mechanistically to identify approaches to modulate other pathogenic populations. It should be noted that our atlas is based on scRNA-seq data, enabling detailed analysis of immune cells. However, cell types such as hepatocytes and HSCs are better captured using single-nucleus RNA-seq^24,25,67–69^ or using our fixed cell profiling on frozen biopsies (Fig. 4a). Future atlases should integrate scRNA-seq, single-nucleus RNA-seq and expanded fixed cell profiling data from patient biopsies at different CLD stages, to fully dissect cellular heterogeneity. Notably, ‘healthy’ scRNA-seq samples may have confounders such as occult liver disease, field effects of disease elsewhere in the liver (for example, colorectal metastases)^70^ or technical artifacts such as tissue ischemia or dissociation^71^. It is therefore crucial that any pathogenic cell types or candidate therapeutic targets identified from single-cell analyses are validated in independent, histologically staged biopsy cohorts, as we have done for OLR1^+^ SAMacs.

In conclusion, by generating a fully annotated human liver scRNA-seq atlas, we have resolved the previously poorly studied heterogeneity in SAMacs and identified OLR1 as a potential target for liver fibrosis. This work forms the basis for a more refined approach to module SAMacs and provides a resource for the identification of additional therapeutic strategies in CLD.

## Methods

This research complies with all relevant ethical regulations. Specific approvals for different aspects of the study are described below.

### Study participants

Local approval for blood samples from patients with CLD for in vitro analyses was obtained from the Lothian NRS Human Annotated Bioresource and Tissue Governance Unit (study SR574), following review at the East of Scotland Research Ethics Service (reference 15/ES/0094). All participants provided written informed consent. No participant compensation was provided. For histological assessment of MASLD biopsies, anonymized unstained formalin-fixed paraffin-embedded liver biopsy sections were provided by the Lothian NRS Human Annotated Bioresource (study SR848). For spatial proteomic analysis, anonymized unstained formalin-fixed paraffin-embedded liver biopsy sections were provided by the Lothian NRS Human Annotated Bioresource (studies SR1865 and SR2248) and Grampian Biorepository (study TR324) under authority from the East of Scotland Research Ethics Service REC 1 (reference 15/ES/0094). For scRNA-seq analysis of liver needle biopsy tissue, snap-frozen samples from the Liver Cell Analysis study were used, under approvals from North West—Greater Manchester West Research Ethics Committee (reference 20/NW/0075). All patients provided written informed consent. No participant compensation was provided. For flow cytometry analysis of human liver macrophages, liver tissue samples were obtained through the Tissue Access for Patient Benefit Initiative at the Royal Free Hospital (approved by the UCL-Royal Free Hospital BioBank Ethical Review Committee references 11/WA/0077, 16/WA/0289 and 21/WA/0388). Unified transparent approval for the pan-Scotland SteatoSITE project 2 was provided by the West of Scotland Research Ethics Committee 4 (reference 20/WS/0002), Public Benefit and Privacy Panel for Health and Social Care (reference 1819-0091), Institutional Research & Development departments and Caldicott Guardians. For analysis of ARLD, bulk RNA-seq data were obtained from the Human Biorepository Core of the NIH-funded InTeam consortium (7U01AA021908-05) and from Cliniques Universitaires Saint-Luc, provided by J.A., E.P. and R.B. All participants provided written informed consent, and the local Ethics Committees and the Institutional Review Board of the University of North Carolina approved the protocols. Patients with ARLD and healthy control samples were then classified according to the histological fibrosis stage on biopsy (grades F0–F4). Relevant clinical data can be found in the source paper^39^. Previously described bulk RNA-seq data from histologically staged PSC biopsy tissue^38^ were provided by J.D.R., G.B., D.L. and A.Z.

### Mice

Adult male C57BL/6JCrl mice aged 8–10 weeks were purchased from Charles River. Mice were housed under specific pathogen-free conditions at the University of Edinburgh. All experimental protocols were approved by the University of Edinburgh Animal Welfare and Ethics Board in accordance with the UK Home Office Legislation, following institutional guidelines in full. For the carbon tetrachloride (CCl_4_) model, CCl_4_ (Sigma-Aldrich, 319961) was diluted 1:3 in olive oil (Sigma-Aldrich, 01514) and injected intraperitoneally twice a week for 4 weeks at a dose of 0.4 μl kg^−1^, as previously described^17^. Mice were randomly assigned to receive CCl_4_ or to serve as healthy controls. No sample size calculation or blinding was performed. For scRNA-seq, blood and liver tissue were obtained for 24 h, 96 h and 4 weeks after the final CCl_4_ injection versus healthy controls. For scATAC–seq, blood and liver tissue were obtained at 24 h or 4 weeks after the final CCl_4_ injection versus healthy controls. For flow cytometry, liver tissue only was obtained at 24 h, 96 h, 168 h and 4 weeks after the final CCl_4_ injection. For the BDL model, mice were randomly assigned to have BDL surgery or to serve as healthy controls. No sample size calculation or blinding was performed. BDL was performed as previously described^72^. For scRNA-seq, blood and liver tissue were obtained on day 14 following BDL versus healthy controls. For the choline-deficient, L-amino acid-defined, high-fat diet (CDAHFD) MASH model^73^, mice were randomly assigned to be placed on CDAHFD diet or to serve as healthy controls. No sample size calculation or blinding was performed. Mice were placed on CDAHFD diet (Research Diets, A06071309) for 10 weeks. Control mice were maintained on standard chow. Liver tissue was obtained for histological analysis. For the generation of bone-marrow chimera mice, donor LY5.1 (B6.SJL-*Ptprc*^*a*^*Pepc*^*b*^/*BoyCrl*) male mice (CD45.1/CD45.2) aged 8–10 weeks were originally obtained from Charles River and bred at the University of Edinburgh. Additional methodological details are described in Supplementary Note 5.

### Preparation of liver and blood single-cell suspensions

For mouse flow cytometry and fluorescence-activated cell sorting (FACS), liver single-cell suspensions were prepared^74^. Remaining protocol details are described in Supplementary Note 5. For human liver flow cytometry, intrahepatic leukocytes were isolated from tissue samples as described^75,76^, and processed as per Supplementary Note 5.

### Flow cytometry and FACS sorting

Mouse flow cytometry methods are described in Supplementary Note 5. Gating strategies for mouse liver macrophage subpopulations are also shown (Extended Data Fig. 8 and Fig. 5g). Human liver flow cytometry staining is described in Supplementary Note 5. Single viable cells (CD45^+^, live/dead blue^−^) were gated on immune cell lineage. Lymphoid lineage cell populations were removed (CD19^−^, CD3^−^, CD56^−^) and the myeloid compartment defined (CD64^+^). TREM2^+^ OLR1^+^ cells were gated within the myeloid compartment gate. Samples with more than 1,000 viable CD64^+^ myeloid cells were included for macrophage quantitation. FACS sorting and sample processing for mouse scRNA-seq and scATAC–seq are described in Supplementary Note 5. Cells from three mice were pooled for each scRNA-seq and scATAC–seq sample.

### Human liver biopsy scRNA-seq

Snap-frozen needle biopsy material (5 mm of 16G biopsy) was stored at −80 °C until use. scRNA-seq of 13 frozen human liver biopsies (7 MASLD and 6 healthy; Supplementary Table 12) was performed using the Chromium Next GEM Single Cell Fixed RNA Hybridization & Library Kit (10x Genomics, PN-1000415), Chromium Single Cell Fixed RNA Human Transcriptome Probe Kit and Chromium Next GEM Chip Q Kit (10x Genomics, PN-2000518) on the Chromium X Single Cell Platform (10x Genomics), according to the manufacturer’s protocol (document CG000527). For liver biopsy fixation and dissociation, samples were processed using the Chromium Next GEM Single Cell Fixed RNA Sample Preparation Kit (10x Genomics, 1000414) as described in the 10x Genomics-demonstrated protocol (document CG000553) with minor modifications. In brief, after overnight fixation, samples were dissociated in RPMI containing 0.2 mg ml^−1^ Liberase TL (Roche, 05401020001) at 37 °C using the following program on the gentleMACS Octo Dissociator (Miltenyi Biotec): spin 50 rpm for 20 min, spin 1,000 rpm for 30 s and spin −1,000 rpm for 30 s. Dissociated samples were passed through a 30 µm filter (Miltenyi Biotec), washed with PBS and resuspended in quenching buffer. Samples were then individually hybridized with unique probes at 42 °C for 20 h. Cells were counted using a NovoCyte flow cytometer (Agilent Technologies) and 20,000–30,000 cells per sample were pooled together. After the posthybridization washes, 200,000 pooled cells were loaded onto each lane of the chip. All libraries were sequenced on an Illumina NovaSeq X Plus 25B PE150.

### Spatial proteomics

Five micrometers of FFPE human liver sections from 46 patients (10 healthy (8 resection and 2 biopsy) and 36 CLD (9 MASLD explant, 4 MASLD biopsy, 9 ARLD explant, 8 PBC explant and 6 PSC explant)) were stained using the antibody panel as listed in Supplementary Table 17. For conjugation of custom antibodies, the Antibody Conjugation kit (Akoya Biosciences, 7000009) was used as described in the Akoya Biosciences-demonstrated protocol (document name PCF User Guide (v.2.1.0)). Sections were rehydrated and antigen retrieval performed in 1x AR9 buffer (Akoya Biosciences, AR900250ML) in a pressure cooker for 20 min. Sections were then stained using the sample kit for PCF (Akoya Biosciences, 7000017) as described in the Akoya Biosciences-demonstrated protocol (document name PCF User Guide (v.2.1.0)). At the end of the protocol, section photobleaching was performed as described in the Akoya Biosciences-demonstrated protocol (document name Autofluorescence Quenching Protocol for PCF). Multiplex immunofluorescence images were acquired using the 10x buffer kit for PCF (Akoya Biosciences, 700019) on the PCF instrument (Akoya Biosciences).

### Immunofluorescence staining

Methods for immunofluorescence staining and analysis of human liver, stem cell-derived three-dimensional (3D) multilineage liver organoids and mouse liver tissue are described in Supplementary Note 5.

### Cell culture

Methods for culture of THP-1 cells, human MDMs, LX2 HSCs, and stem cell-derived 3D multilineage liver organoids are described in Supplementary Note 5. Analysis was performed by qPCR, ELISA, or MTT assay (Supplementary Note 5).

### Primary liver cell 3D multilineage liver spheroid MASH model

Spheroid culture of primary human liver cells was conducted as previously described^57^, with relevant modifications. Primary human hepatocytes (PHH), stellate cells and liver macrophages were seeded at a PHH:macrophage:stellate cell ratio of 6:1.5:1 into ULA 96-well plates (Corning) in liver culture medium (William’s E medium containing 11 mM glucose, 10 ng ml^−1^ (controls) or 10 μg ml^−1^ insulin (MASH), 100 nM dexamethasone, 5.5 mg l^−1^ transferrin, 6.7 μg l^−1^ selenite, 2 mM L-glutamine, 100 U ml^−1^ penicillin, 0.1 mg ml^−1^ streptomycin) supplemented with 10% FBS at a total density of 1,875 cells per well. Serum was phased out after 5 days, once spheroids had formed. Culture media was exchanged every 2–3 days. Spheroids were transfected with siRNA against OLR1 Silencer Select Validated siRNA (Ambion, 4390824) or Silencer Select Negative Control No. 1 siRNA (Ambion, 4390843) at final concentrations of 50 nM using RNAiMAX (Invitrogen) according to the manufacturer’s instructions. The respective siRNA was mixed with serum-free Opti-MEM medium, and the mixture was incubated at room temperature for 20 min. Subsequently, the siRNA was added to the cells at the time of seeding as previously described^77,78^. For MASH induction, media was supplemented with FFAs, and 240 μM oleic acid and 240 μM PA were conjugated to 10% BSA at a molar ratio of 1:5 for 2 h at 40 °C before being added to the spheroid medium at day 5 as indicated.

### scRNA-seq analysis

#### Human data processing, QC and integration

Human scRNA-seq data from eight previously published studies^13,23–28,79^, were downloaded from the European Nucleotide Archive as paired FASTQ files (210 samples from 81 patients; Supplementary Table 1). Sequence alignment and initial sample processing were performed using Cell Ranger (v.7.0.0)^80^ and the GRCh38 reference human genome (v.GRCh38-2020-A downloaded from 10x Genomics) to generate feature barcode matrices. Subsequent processing and analysis were performed in R (v.4.3.1) using the R package Seurat (v.5.0.0)^81^, and in Python (v.3.10.16). All visualizations were produced using Seurat functions in conjunction with the ggplot2 (v.3.5.1)^82^ and grid (v.4.3.2; R Core Team (2020)) R packages.

Samples were adjusted for ambient RNA contamination using the R package DecontX (v.1.14.2)^83^, and cells failing quality thresholds (mitochondrial gene percentage >15% or total unique features <100) were removed. Patients with <500 cells passing these thresholds were excluded. As a result, all patients mentioned in ref. ^79^ were excluded, leaving 77 patients from seven studies for downstream analysis.

For patients whose genetic sex was not reported in the original publications, sex was inferred based on the expression of 20 Y chromosome-specific genes—*RPS4Y1*, *DDX3Y*, *KDM5D*, *UTY*, *USP9Y*, *ZFY*, *EIF1AY*, *TSPY1*, *PRKY*, *NLGN4Y*, *AMELY*, *PCDH11Y*, *CDY1*, *CDY2*, *VCY*, *BPY2*, *DAZ*, *HIST1H2AY*, *RBMY* and *SRY*. A total of 50 cells were randomly selected from each patient, and, if any of these genes had nonzero expression in the selected cells, the patient was classified as male; otherwise, as female.

Patient disease status was obtained either from the provided metadata or from patient characteristics reported in each study. Liver samples from patients annotated as healthy or those with no fibrosis reported on histology were considered healthy controls, whereas those annotated as diseased or with documented fibrosis on histology were classified as CLD. Where available, the etiologies of CLD were extracted from the original metadata or were inferred from published histological findings. All other patients with unspecified etiology were annotated as unknown.

To facilitate efficient integrative analysis of all cells from the 77 remaining patients, 1,000 representative cells were first selected from each sample using the Seurat function ‘SketchData’ based on a method described in ref. ^84^. Normalization, scaling, variable feature identification and principal component analysis (PCA) were performed on the ‘sketched’ data using default Seurat methods, followed by integration using Harmony^85^ and clustering using Seurat. Using the sketched integration as reference, the Seurat function ‘ProjectIntegration’ was then used to integrate the entire dataset. Cluster labels from the sketched cells were projected onto the whole dataset using the function ‘ProjectData’ from Seurat.

Wilcoxon rank-sum tests were performed using the ‘FindAllMarkers’ function from Seurat, identifying marker genes from each cluster. Clusters were then manually annotated using previously published literature, identifying 11 cell lineages.

#### Clustering, annotation and atlas construction

Cell lineages were subset individually and the previous preprocessing steps repeated (normalization, scaling, variable feature identification and PCA). As before, cells were clustered and then annotated based on the expression of known marker genes. Doublet clusters were identified manually based on their expression of multiple lineage markers and removed. Low-quality clusters (median nFeature < 800) were also removed. After QC for each lineage, the final atlas included 649,295 cells.

Annotations from each cell lineage (subsequently referred to as ‘level 3’ annotation) were mapped back to the complete atlas. Cluster similarities were assessed using the function ‘buildSNNGraph’ from R package scran (v.1.30.2)^86^ and the function ‘pariwiseModularity’ from R package bluster (v.1.12.0)^87^. Based on the results, clusters from level 3 annotation were manually merged into 34 grouped clusters, defined as the ‘level 2’ annotation. The same similarity testing and merging procedures were then applied to the level 2 clusters, resulting in the manual identification of 16 ‘level 1’ clusters.

For atlas visualization, a ShinyApp was built using R package ShinyCell (v.2.1.0)^88^ and a Loupe file was generated using LoupeR (v.1.1.4; https://github.com/10XGenomics/loupeR).

#### Differential abundance and expression testing

Differential composition analysis was performed on level 3 annotations of selected lineages using MiloR (v1.8.1)^30^. First, cellular states were modeled as neighborhoods based on the *k*-nearest neighbor graph. Differential neighborhood abundance testing was then performed comparing disease status (healthy versus CLD). For the MP lineage, additional differential abundance tests were also performed for each level 3 MP cell type, comparing healthy versus CLD, male versus female and healthy versus each annotated disease etiology. Analysis was restricted to patients with more than 50 MPs. The relative abundance of each cell type was calculated for each patient, and relative abundances among groups were tested using unpaired two-sample *t* tests, implemented through the ‘stat_compare_means’ function from the ggpubr R package. Differential gene expression analysis was performed comparing SAMac1 and SAMac2 clusters using the ‘model-based analysis of single-cell transcriptomics’ framework^89^, deployed using the Seurat function ‘FindMarkers’.

#### Cell–cell interaction analysis

Ligand–receptor analysis was performed on the level 3 atlas using Python packages liana-py (v.1.4.0)^43^ and cell2cell (v.0.7.4)^43^. To accommodate Python analysis, the dataset was transformed from a Seurat object to H5AD format using R packages sceasy (v.0.0.7) and reticulate (v.1.35.0). Analysis was limited to the MP clusters SAMac1 and SAMac2 (as senders) and level 3 mesenchymal cell clusters (as receivers). Ligand–receptor score calculations were iteratively run across individual patient samples using the liana-py function ‘rank_aggregate.by_sample’. Ligand–receptor scores were input to cell2cell as a four-dimensional tensor (sample × sender cell × receiver cell × ligand–receptor pair). Using the Tensor–cell2cell pipeline, tensor decomposition was performed, extracting unique context-dependent patterns of intracellular communication in the form of ten factors. Before decomposition, elbow analysis was performed to select the optimal number of factors. Ligand–receptor pairs associated with each factor were retrieved using the cell2cell function ‘tensor_downstream.get_lr_by_cell_pairs’.

#### Human biopsy scRNA-seq analysis

Analysis was performed on 13 human liver needle biopsy samples (*n* = 6 healthy and *n* = 7 MASLD). Sequence alignment was performed using Cell Ranger (v.7.0.0) and the GRCh38 reference human genome (version GRCh38-2020-A downloaded from 10x Genomics). Subsequent processing was performed in R (v.4.4.1) using R package Seurat (v.5.3.1.0000). Visualizations were produced using Seurat functions in conjunction with the R package ggplot2 (v.3.5.2). Cells with total unique features of <500 were removed. Normalization, scaling, variable feature identification and PCA were performed using default Seurat methods. Samples were integrated using R package Harmony (v.1.2.3). Clustering was performed iteratively, identifying and removing doublet clusters (based on their expression of multiple lineage markers), low-quality clusters (based on their high proportion of mitochondrial gene expression and low unique feature count), as well as proliferating cells. The final dataset included 71,062 liver cells. Ambient RNA removal was performed using SoupX^90^, provided with clustering from Seurat and manual cell-type annotations, using ALB as a marker of the hepatocyte lineage. Label transfer was performed on the full dataset using Seurat functions ‘FindTransferAnchors’ and ‘TransferData’, and the human liver cell atlas as reference.

#### Mouse CCl4 scRNA-seq analysis

Analysis was performed on 12 mouse scRNA-seq datasets (*n* = 6 CD45^+^ cells from liver and *n* = 6 CD45^+^ cells from blood). Sequence alignment and initial sample processing were performed using Cell Ranger (v.3.0.2)^80^ and the mm10 reference mouse genome (version mm10-3.0.0 downloaded from 10x Genomics). Subsequent processing was performed in R (v.4.2.2) using the R package Seurat (v.4.1.3). Visualizations were produced using Seurat functions in conjunction with the ggplot2 (v.3.2.1)^82^ and grid (v.3.6.3; R Core Team (2020)) R packages. Cells failing quality thresholds (mitochondrial gene percentage >30% or total unique features <300) were removed. Normalization, scaling, variable feature identification and PCA were performed using default Seurat methods. After merging the 12 datasets, dimensional reduction was performed (uniform manifold approximation and projection). Clustering was performed iteratively, identifying and removing doublet clusters based on their expression of multiple lineage markers. After QC, the final dataset included 27,070 cells. Clustering of the final dataset was then performed using default methods, and marker genes for each cluster were identified by Wilcoxon rank-sum test, facilitating manual annotation of cell types. The MP lineage was isolated and re-analyzed as described above (normalization, scaling, variable feature identification and PCA). Clustering was then performed using the first 17 principal components and a resolution of 0.7. Annotation was performed manually based on the expression of known marker genes. Differentially expressed genes between SAMac1 and SAMac2 clusters were identified using the ‘model-based analysis of single-cell transcriptomics’ approach^89^. Gene lists were filtered to include only genes that are expressed in >10% cells with an absolute log-transformed fold change >0.5 and Bonferroni-adjusted *P* value <0.01.

#### Mouse BDL scRNA-seq analysis

Analysis was performed on eight mouse scRNA-seq datasets (*n* = 4 CD45^+^ cells from liver, and *n* = 4 CD45^+^ Ly-6G^−^ cells from blood). Sequence alignment was performed using Cell Ranger (v.7.0.0) and the mm10 reference mouse genome (version mm10-2020-A downloaded from 10x Genomics). Subsequent processing was performed in R (v.4.4.1) using R package Seurat (v.5.3.1.0000). All visualizations were produced using Seurat functions in conjunction with the R package ggplot2 (v.3.5.2).

Processing was performed as described for human biopsy scRNA-seq. In brief, samples were integrated using Harmony, clustered and filtered, including removal of proliferating and nonimmune cell populations. The final dataset included 27,789 immune cells. Annotation was performed manually, based on the expression of known marker genes. Ambient RNA removal was performed using SoupX (v.1.6.2), provided with cell-type annotations, and Csf1r as a marker of the MP lineage. Integration using Harmony was repeated after ambient RNA removal, followed by clustering. Clusters expressing MP lineage markers were subsetted and reintegrated, creating a final MP dataset of 17,240 cells. Label transfer was performed using the Seurat functions ‘FindTransferAnchors’ and ‘TransferData’, and the annotated mouse CCl4 scRNA-seq dataset as reference.

#### Mouse scATAC–seq analysis

Computational methods are described in Supplementary Note 5.

### Human liver bulk RNA-seq analysis

SteatoSITE RNA-seq and histology data were generated as described in detail in the original paper^2^. For correlation between *OLR1* gene expression and time-to-event analysis, biopsy cases with available RNA-seq from the SteatoSITE multimodal database^2^ were used in R (v.4.4.3). Normalized counts for OLR1 were available as part of the dataset^2^. Decompensation events in the clinical data extract were defined by a combination of ICD codes and UK OPCS-4 codes identifying procedures relating to cirrhosis-related hospital admissions activity as previously described^91^. Decompensation event analysis was only conducted on biopsy cases for which the first decompensation-related coding was present in the clinical data extract after the biopsy date and analysis were conducted using death as a competing risk. The cutpoint for *OLR1* gene activity or SAMac2 proportion was calculated using surv_cutpoint() from the ‘survminer’ (v.0.4.9) package, applying maximally selected rank statistics of the ‘maxstat’ package (v.0.7.25) with a minimum proportion of 0.25. Kaplan–Meier estimator curves of all-cause mortality or decompensation events for assigned high/low risk groups were compared by log-rank testing in ‘survminer’.

The second MASLD dataset^37^ was downloaded from NCBI (GSE135251). The PBC dataset was downloaded from Zenodo^40^. The ARLD dataset was provided by J.A. and R.B. The PSC dataset was provided by Gilead and analyzed by J.D.R. Raw count matrices and sample metadata table of each dataset were imported and analyzed using R (v.4.3.2). Raw count data were variance stabilized using a variance-stabilizing transformation (VST) with the vst() function in the sctransform (v.0.4.2) package. Next, PCA was performed on the VST-transformed expression values, and sample clustering patterns were examined. Samples that segregated separately in PCA space (PC1/PC2) were considered potential outliers and were excluded from downstream analyses. Gene expression levels were normalized using the trimmed mean of M-values (TMM) method by calcNormFactors() function (method = ‘TMM’) from the edgeR (v.3.42.4) package. Normalized expression values were expressed as counts per million (CPM) using TMM-adjusted library sizes through the cpm() function (normalized.lib.sizes = TRUE, log = FALSE). Statistical analysis of expression differences in TMM-normalized CPM values for OLR1 across samples grouped by fibrosis stage was calculated by Spearman correlation tests (MASLD and PSC) performed using the cor.test() function (method = ‘spearman’, exact = FALSE) from the stats (v.4.3.2) R package, or group-wise comparisons (for ARLD and PBC) using the Wilcoxon rank-sum test adjusted for multiple comparisons using the Benjamini–Hochberg (BH) method, implemented through the wilcox_test() function in the rstatix (v.0.7.2) R package.

### Deconvolution of bulk RNA-seq data

Bulk RNA-seq deconvolution was performed using the BayesPrism (v.2.2) R package^92^. To generate the single-cell reference, raw count matrices were reconstructed for the MP Seurat object. Seven cell types were used as cell.type.labels, which include tissue monocytes, *STAB1*^+^ macrophages, SAMac1, SAMac2, cDCs, KCs and transitioning macrophages. Deconvolution was performed using new.prism() with default settings for model fitting and outlier handling, and final cell-type fractions were obtained from the posterior distribution. Estimated proportions across fibrosis score or fibrosis stage groups were visualized using box plots generated with ggplot2 package. Statistical testing for differences across fibrosis stages was performed using Wilcoxon rank-sum tests adjusted for multiple comparisons using the BH method.

### Human MDM microarray analysis

Human monocyte/macrophage gene expression data were processed as described^56^. In brief, microarray data from GEO accession GSE47189 (ref. ^55^) were downloaded using R package GEOquery. Gene set variation analysis was performed using the R package^93^, and the top 100 differentially expressed genes between SAMac1 and SAMac2 were used as the gene sets. The expression of pairs of genes was correlated across all conditions using Pearson correlation.

#### Spatial proteomic analysis

Qptiff files from the PCF were registered into spatial data zarr (v.2) using Sopa (v.2.0.8)^94^. Cell segmentation was performed using Cellpose (v.4.0.7)^95^. A cell segmentation layer was generated using DRAQ5 as nucleus channel and a composite channel of HLA-A, ACTB, ECAD, CD45 and CD68 for the membrane channel through 99th percentile rescaling and Contrast Limited Adaptive Histogram Equalization of each channel, followed by merging. Mean pixel intensity was extracted from each cell segmentation mask, yielding a cell × protein matrix that was analyzed in Seurat (v.5.2.1). Areas with artifactual staining and cells with low raw DAPI expression or area of <120 px^2^ or >4,000 px^2^ were removed (pixel to µm = 1:0.5050). Each sample was individually normalized using center log ratio for each feature independently. Samples were then integrated, scaled and PCA computed. Batch correction was done using reciprocal PCA with FindIntegrationAnchors and IntegrateData functions using default parameters. Clustering and cell-type annotation were then performed similarly to a previously described method^96^. In brief, clustering was done at a coarse lineage level followed by iterative cell subtype clustering. Clusters were manually annotated based on marker expression. Clusters that could not be easily annotated were subjected to manual gating of known markers (for example, T cell subtype markers). To address the issue of lateral spillover (that is, bright expression from one cell type may ‘spill’ into its neighbor, thereby causing that cell to be mislabeled), we visualized the markers on the images using Napari. Annotations of the clusters that represented lateral spillover artifacts were corrected to the appropriate cell type. Finally, for clusters that had no reliable annotation after multiple rounds of gating, we performed a final round of clustering and label transfer using FindTransferAnchors and MapQuery on default parameters from Seurat. Any remaining cells without a reliable label following this process were annotated as unknown. To confirm annotation accuracy, all cluster labels were then manually examined and confirmed on the staining images. Cell density was calculated as the number of cells per mm^2^ tissue area by converting pixel to mm^2^ using conversion factor (0.5050 µm pixel^−1^). Cell-type proportions were computed for each patient and condition by dividing the number of cells of each type by the total number of cells within the grouping unit (patient or condition). Cellular niche analysis on annotated spatial object was conducted using the scNiche package (v.1.1.1)^97^, using suggested parameters at kcutoff = 30, target_ k (clusters) = 3. Cell-type enrichment within spatial niches was assessed by dividing observed proportion of each cell type by the expected proportion. Then, enrichment was quantified by log_2_(O/E) = log_2_((mean observed + *ε*) / (mean expected + ε)), where *ε* = 10^−6^. Niche area was calculated by the sum of all cells from the niche as a percentage of total sample area per patient. Patient-level mean intensity values were computed for COL1 and COL3 across all cells assigned to a particular niche. Statistical analysis was performed in R. For comparisons of cell-type density across multiple etiology groups, one-way analysis of variance was used followed by Dunnett’s post hoc test for pairwise comparisons against the control group using the multcomp package (v.1.4-29). For comparisons across spatial niches, Kruskal–Wallis test was applied followed by pairwise Wilcoxon rank-sum tests with BH adjustment. Two group comparisons (control versus CLD) were performed using Wilcoxon rank-sum tests.

### Statistics and reproducibility

All nonbioinformatics data were analyzed using GraphPad Prism 10 software. All graphs are shown as mean ± s.e.m. Normality was tested using the Shapiro–Wilk test, and subsequent parametric and nonparametric statistical analyses were chosen accordingly. No statistical method was used to predetermine sample sizes. Sample sizes for in vivo mouse work are similar to those previously reported in mouse CLD^17^. Computational data excluded from analyses (for example, cells failing QC thresholds) are described in subsections in Methods. Human experiments were not randomized but mice were randomly allocated to different treatment groups. Data collection and analysis were not performed blindly to the conditions of the experiments. Statistically significant comparisons are highlighted as *P ≤ 0.05, **P ≤ 0.01, ***P ≤ 0.001, ****P ≤ 0.0001.

### Reporting summary

Further information on research design is available in the Nature Portfolio Reporting Summary linked to this article.

## Online content

Any methods, additional references, Nature Portfolio reporting summaries, source data, extended data, supplementary information, acknowledgements, peer review information; details of author contributions and competing interests; and statements of data and code availability are available at https://doi.org/10.1038/s41588-026-02774-w.

## Supplementary information

Supplementary Information Supplementary Notes 1–5, Supplementary Discussion and Supplementary Figs. 1–3.

Reporting Summary

Peer Review File

Supplementary Tables Supplementary Table 1: Summary of metadata for patients included in human scRNA-seq analysis. Supplementary Table 2: Level 1 cluster marker genes from human scRNA-seq analysis. Supplementary Table 3: Level 2 cluster marker genes from human scRNA-seq analysis. Supplementary Table 4: Level 3 cluster marker genes from human scRNA-seq analysis. Supplementary Table 5: Level 3 MP marker genes from human scRNA-seq analysis. Supplementary Table 6: Level 3 mesenchyme marker genes from human scRNA-seq analysis. Supplementary Table 7: Level 3 endothelial marker genes from human scRNA-seq analysis. Supplementary Table 8: Level 3 T/NK cell marker genes from human scRNA-seq analysis. Supplementary Table 9: Level 3 B cell marker genes from human scRNA-seq analysis. Supplementary Table 10: Level 3 epithelial marker genes from human scRNA-seq analysis. Supplementary Table 11: Differentially expressed genes between human SAMac1 and SAMac2. Supplementary Table 12: Patient metadata for liver needle biopsies used for scRNA-seq analysis. Supplementary Table 13: Cluster marker genes for mouse MPs. Supplementary Table 14: Differentially expressed genes between mouse SAMac1 and SAMac2. Supplementary Table 15: TF motif enrichment in mouse SAMac1 and SAMac2 from scATAC–seq data. Supplementary Table 16: Ligand–receptor interaction analysis from human scRNA-seq data between SAMac1 and SAMac2 as senders and level 3 mesenchyme as receiver cells. Summary of ligand–receptor pairs in each factor. Supplementary Table 17: Antibodies used in this study. Supplementary Table 18: qPCR primers used in this study.

Supplementary Data Statistical supporting data for Supplementary Fig. 3.

## Source data

Source Data Figs. 3–5 and 7 and Extended Data Figs. 5–8 and 10 Statistical source data.

## Data Availability

Human scRNA-seq data were analyzed from the following publicly available datasets: GSE243981 (ref. ^24^), GSE168933 (ref. ^26^), GSE192742 (ref. ^25^), GSE115469 (ref. ^23^), E-MTAB-10143 (ref. ^27^), GSE136103 (ref. ^13^), GSE181483 (ref. ^28^) and GSE129933 (ref. ^79^). An online data browser for annotated human scRNA-seq data is avallabile at https://shiny.igc.ed.ac.uk/Human_Liver_scRNA-seq_Atlasv2/. A publicly accessible browser for mouse CCl_4_ MP data is available at https://shiny.igc.ed.ac.uk/mouseMPshinyApp/. Loupe browser compatibile files and annotated Seurat objects for human atlas and mouse MP CCl_4_ scRNA-seq data are available at https://doi.org/10.7488/ds/8163 and https://doi.org/10.7488/ds/8165, respectively. Raw FASTQ files for human biopsy scRNA-seq, mouse scRNA-seq and mouse scATAC–seq are accessible at GEO (GSE298719). Hepatic bulk RNA-seq data from SteatoSITE cohort are deposited in the European Nucleotide Archive (https://www.ebi.ac.uk/ena; study accession PRJEB58625). SteatoSITE gene expression data are also freely available for user-friendly interactive browsing online at https://shiny.igc.ed.ac.uk/SteatoSITE_gene_explorer/. Other bulk RNA-seq data and metadata were downloaded directly for MASLD^37^ and PBC^40^ or provided by the original investigators for PSC and ARLD cohorts. Human MDM microarray data were downloaded from the GEO accession GSE47189. Source data are provided with this paper.

## Code availability

All codes for the scRNA-seq, scATAC–seq, bulk RNA-seq and spatial proteomic analyses are available at https://git.ecdf.ed.ac.uk/ramachandran-lab/olr1-paper and on Zenodo (https://doi.org/10.5281/zenodo.21997255)^98^.
