# Supplementary material for: A human single-cell atlas identifies OLR1+ scar-associated macrophages as a potential therapeutic target for chronic liver disease
Source: Nat Genet. 2026 Sep 28;58(10):2550–65. doi: 10.1038/s41588-026-02774-w (PMC13649586; doi:10.1038/s41588-026-02774-w)
Supplement: Supplementary file 1 [file 41588_2026_2774_MOESM1_ESM.pdf]

nature genetics

Supplementary information

https://doi.org/10.1038/s41588-026-02774-w

A human single-cell atlas identifies OLR1$^+$ scar-associated macrophages as a potential therapeutic target for chronic liver disease

In the format provided by the authors and unedited

Supplementary Information

Table of Contents

Supplementary Information **......... 1**

Supplementary Note 1 – Human scRNAseq QC and Integration 2

Supplementary Note 2 – Mouse Bone Marrow Chimeras......... 2

Supplementary Note 3 – Mouse scATACseq Analysis 2

Supplementary Note 4 – Human Multilineage Spheroids 2

Supplementary Discussion......... 3

Supplementary Note 5 -Methods......... 4

Mouse Bone Marrow Chimera Generation 4
Preparation of liver and blood single-cell suspensions 4
Flow Cytometry and FACS sorting 5
Mouse scRNAseq and scATACseq 6
Immunofluorescence staining......... 6
Cell culture......... 7
OLR1 knockdown/inhibition in macrophages......... 8
Hepatic stellate cell stimulation assay 8
Stem cell-derived 3D multilineage liver organoid 9
RNA extraction and RT-qPCR......... 9
ELISA 9
MTT proliferation assay......... 9
Mouse scATACseq analysis......... 10

Supplementary Figures 11

Supplementary References 16

Supplementary Note 1 – Human scRNAseq QC and Integration

Following initial QC and data integration of individual samples, we generated a lineage annotation overview based on previously defined canonical marker genes (Supplementary Figure 1A, B). Integration was effective across studies (Supplementary Figure 1C). Each lineage was then subclustered at high resolution to identify and remove low quality (median nFeatures<800) and doublet clusters (co-expression of multiple lineage marker genes), followed by manual annotation of cell types within each lineage, based on curated marker gene expression and previous literature. Annotated lineage subclusters were then combined to generate the final level 3 liver atlas, consisting of 649,295 high quality single cells partitioned into 125 annotated clusters reflective of different cell states.

Supplementary Note 2 – Mouse Bone Marrow Chimeras

To confirm the origin of SAMac subpopulations we generated busulfan-conditioned bone-marrow chimeric mice[1] (Extended Data Fig. 8B), with Ly5.1 (CD45.1+) donor mice enabling the use of CD45.1 expression to to track bone-marrow derived donor cells. A flow cytometry gating strategy was developed to study donor cell contribution to liver myeloid subpopulations including Ly-6C^{hi} monocytes, Timd4^{+}Vsig4^{+} resident KCs, Timd4^{-}Vsig4^{+} monocyte-derived KCs (MoKCs) and the 2 SAMac subpopulations (Extended Data Fig. 8C). Using this system there was minimal replenishment of resident KCs under homeostatic conditions (Extended Data Fig. 8D). Following chronic CCl_4, Timd4^{+} resident KCs remained mainly of host origin, suggesting minimal monocyte contribution to this population (Extended Data Fig. 8E). In contrast, SAMacs and MoKCs were almost entirely derived from donor bone marrow, with no difference in chimerism between the 2 SAMac subpopulations (Extended Data Fig. 8E).

Supplementary Note 3 – Mouse scATACseq Analysis

To identify mouse MPs for detailed scATAC-seq analysis, we performed initial QC and then broad clustering of all merged mouse blood and liver scATACseq data (Extended Data Fig. 9A). These scATACseq clusters were computationally annotated according to cell lineage via a label transfer from mouse CCl4 scRNAseq dataset (Extended Data Fig. 9B-C). ScATACseq clusters annotated as MPs by label transfer were then subsetted for more detailed clustering, annotation and tranfscription factor motif enrichment analysis.

Supplementary Note 4 – Human Multilineage Spheroids

To determine whether OLR1+ MDMs have a functional role in liver fibrosis, we developed a human multilineage spheroid culture system, containing embryonic stem (ES) cell-derived hepatocytes, hepatic stellate cells and endothelial cells as previously described[2–4], coupled with the addition of primary human monocytes from patients with CLD (Supplementary Figure 3A). The addition of monocytes, followed by 7 days of monocyte differentiation and spheroid maturation *in vitro*, resulted in engraftment of IBA1+ MDMs embedded within the spheroids (Supplementary Figure 3B). The addition of patient monocytes increased spheroid expression of SAMac2 markers *OLR1*, *SPP1* and *CD68,* and promoted spontaneous fibrogenic activity with higher levels of *COL1A1* following 1 week of maturation (Supplementary Figure 3C). No alteration was observed in hepatocyte gene *TTR*. To confirm that this spheroid system could be utilised to test the antifibrotic effects of drug therapy, the TGFβ signalling inhibitor A83-01 or vehicle control were administered to spheroids during the treatment phase, between day 8 and day 15 (Supplementary Figure 3A). TGFβ inhibition attenuated fibrogenic gene expression (*COL1A1* and *COL3A1*) in macrophage-containing

spheroids (Supplementary Figure 3D). We then proceeded to test the functional impact of MDM depletion, via administration of the of the CSF1R inhibitor Pexidartinib to spheroids during the treatment phase. CSF1R blockade significantly reduced *OLR1*, *IL1B*, *SPP1* and *CD68* and attenuated fibrillar collagen (*COL1A1* and *COL3A1)* gene expression (Supplementary Figure 3E), suggesting that human OLR1^{+} MDMs have a pro-fibrotic function.

Supplementary Discussion

We have highlighted OLR1 as a potential driver of inflammasome activation, IL-1β production and pro-fibrogenic activity of SAMac2. However, the ligands which activate OLR1 signalling in the CLD liver are yet to be defined. Oxidised LDL (oxLDL), the best described OLR1 ligand, accumulates in the CLD liver and could potentially drive SAMac2 activation and pro-inflammatory mediator production[12]. However, OLR1 is a pleiotropic scavenger receptor with numerous candidate ligands including phospholipids, activated platelets, C-reactive protein and heat shock proteins[13]. Hence, further work is needed to dissect which OLR1 ligands are most pertinent in CLD pathogenesis and how they might be selectively inhibited. A more exhaustive characterisation of the functional impacts of OLR1 inhibition on the macrophage secretome and differentiation will also provide important further insights into the antifibrotic effects of targeting SAMac OLR1. It is important to note that many existing CLD cellular models often suffer potential confounders, for example the lack of direct cell-cell contact in our conditioned media experiments or the need for dexamethasone administration in the human spheroid MASH model utilized in this manuscript. Going forwards, additional functional work on macrophage OLR1 modulation should be conducted in multiple discrete CLD models, including *in vivo* mouse models of different aetiologies and additional human cellular and tissue CLD models (e.g. precision cut liver slices). Such work is crucial to contextualize when OLR1 inhibition is likely to be beneficial.

OLR1 has been proposed as a prognostic biomarker for various types of cancer[9] and for cardiovascular risk[10]. Our data suggest that hepatic OLR1 levels may also serve as a prognostic biomarker for CLD. One previous study suggested that elevated soluble OLR1 in the circulation could differentiate patients with MASLD from healthy controls, although the degree of fibrosis or macrophage accumulation in these patients was unclear[11]. While we have been unable to demonstrate differences in soluble OLR1 between healthy and advanced CLD patient serum using a commercial assay (data not shown), the potential for OLR1 as a prognostic biomarker should be investigated in larger patient cohorts using optimised immunoassays. The prognostic value of quantifying OLR1 (and/or other SAMac2 markers) in liver biopsy tissue via immunohistochemistry should also be assessed in patients with different causes of CLD. Ultimately, the accumulation of OLR1+ SAMacs may define subgroups of CLD patients who are more likely to respond to specific anti-inflammatory therapies targeting SAMac2.

A key feature of OLR1+ SAMac2 in both human and mouse CLD is elevated expression of inflammasome components including the pro-inflammatory cytokine IL-1β. Macrophage-derived IL-1β is known to promote mesenchymal cell activation and fibrogenesis in the liver[5] and other human tissues such as heart[6,7]. However, while it has previously been unclear how to selectively attenuate macrophage IL-1β production in the fibrotic niche, our finding that targeting OLR1 on MDMs can reduce IL-1β production and fibrogenic activity. It would be interesting to explore whether macophage OLR1 may potentially have a broader role as an anti-inflammatory target in fibrotic diseases in other tissues such as heart, lung and kidney

which also show SAMac accumulation[8]. An integrated scRNAseq atlas and the application of high-resolution spatial transcriptomics across fibrotic human organs would allow a more detailed assessment of which specific diseases demonstrate *OLR1* expression on IL-1β+ SAMacs. It will be important to include samples from different disease stages and etiologies in such analyses, to elucidate when in the disease trajectory targeting of pro-inflammatory SAMac will be most efficacious.

Supplementary Note 5 -Methods

Mouse Bone Marrow Chimera Generation

To generate bone marrow chimeric mice, busilvex (Pierre Fabre, 728981) was diluted to 1 mg/ml in sterile PBS and injected intraperitoneally to a final dose of 10 mg/kg in three-month-old male C57BL/6JCrl mice (CD45.2/CD45.2) over four consecutive days. On day 5, bone marrow cells were isolated from LY5.1 male mice (CD45.1/CD45.2). Cells were then enriched for hematopoietic stem cells using the EasySep™ Mouse Hematopoietic Progenitor Cell Isolation Kit (STEMCELL Technologies, 19856), according to manufacturer's instructions. Next, 8 x 10^5 enriched cells were injected intravenously into the conditioned C57BL/6JCrl mice. Mice were allowed to recover for 6 weeks prior to CCl_4 administration.

Preparation of liver and blood single-cell suspensions

In brief, livers were perfused with ice cold PBS via the Inferior Vena Cava (IVC) and then excised and placed in RPMI (Gibco, 21875034). The livers were mechanically chopped into small fragments with a razor blade, followed by digestion in RPMI and an enzyme cocktail containing 0.625 mg ml^{-1} collagenase D (Roche, 11088882001), 0.85 mg ml^{-1} collagenase V (Sigma-Aldrich, C9263-1G), 1 mg ml^{-1} dispase (Gibco, Invitrogen, 17105-041) and 30 U ml^{-1} DNase (Roche, 10104159001) at 37°C for 20 min with agitation. Next, the suspension cells were strained through 100μm filters, centrifuged (300g 5 min) and incubated for 3 minutes in 10% red blood cell (RBC) lysis buffer (BioLegend, 420301). After centrifugation (300g 5 min), cells were resuspended in PEB buffer (PBS containing 0.05% FBS and 0.5 mM EDTA) and passed through a 35 μm filter, prior to blocking and antibody staining. Mouse peripheral blood (100-200μL) was collected from the inferior vena cava into tubes containing a 0.5 mM EDTA solution. Blood was incubated twice in 10% RBC lysis buffer (BioLegend, 420301) for 5 minutes on ice. Cells were then centrifuged (300g 5 min), washed in PEB buffer and filtered through a 35 μm filter, prior to blocking and antibody staining.

For human liver flow cytometry, tissue samples were dissociated and incubated at 37 °C, 5% CO_2 in $HBSS^{+/+}$ (Life Technologies; Thermo Fisher Scientific) containing 0.002% DNase I (Roche; Sigma-Aldrich; Merck) and 0.02% collagenase IV (Thermo Fisher Scientific). After enzymatic digestion, tissue samples were subjected to mechanical disruption using the GentleMACS (Miltenyi Biotech) dissociator and debris removed by filtration through a 70 μM filter (Griener). Parenchymal cells were removed by centrifugation on a 30% Percoll gradient (GE Healthcare; VWR) followed by leukocyte isolation by a further round of density centrifugation using a Pancoll gradient (PAN Biotech).

Flow Cytometry and FACS sorting

For mouse liver cell flow cytometry, 2 x 10^6 liver suspension cells were blocked with anti-mouse CD16/32 antibody (BioLegend, 101302; 1:100) and 10% mouse serum (Sigma-Aldrich, M5905) for 10 minutes at 4°C. Cells were then incubated with primary antibodies for 20 minutes at 4°C. All antibodies, catalogue numbers and dilutions used are listed in Supplementary Table 17. Cells were then centrifuged (300G 5 min), washed in PEB buffer and incubated with streptavidin BV650 (BioLegend 405232; 1:100) for 20 at 4°C. Flow cytometry compensations were set up using single stained UltraComp eBeads (Thermo Fisher Scientific, 01-2222-42). Controls for gating included 'fluorescence-minus-one' (FMO) samples. DAPI (1:1000) and counting beads (Biolegend, 424902; 50ul) were added immediately before sample acquisition.

For mouse intracellular flow cytometry of IL-1β, 5 x 10^6 isolated liver cells were incubated with 1ul/ml Momensin (BD Bioscience, 554724) in RPMI (Gibco, 21875034) containing 10% FBS (Thermo Fisher Scientific, 10500-064) for 3 hours at 37°C. Control samples were incubated for 3 hours at 4°C. Following incubations, cells were centrifuged (500g, 5 min) and stained with Zombie NIR viability dye (BioLegend, 423105, 1:1000). Thereafter, cells were washed with PEB buffer. Blocking and primary antibody staining were then performed as described above. All antibodies, catalogue numbers and dilutions used are in Supplementary Table 17. After surface antibody staining, cells were stained with streptavidin-BV421 (BioLegend 405225; 1:100) for 20 minutes at 4°C. Cells were then washed twice in PEB buffer before overnight incubation in fixation buffer (Invitrogen 00-5521-00) at 4°C. After fixation, cells were washed twice with permeabilisation buffer (Invitrogen, 00-8333-56) (1000g, 3 min). Next, cells were incubated with the IL-1β antibody (Supplementary table 17) in permeabilisation buffer for 60 minutes at 4°C. Cells were washed again twice with permeabilisation buffer (Invitrogen, 00-8333-56) (1000g, 3 min) and then resuspended in PEB buffer, before acquisition. For mouse peripheral blood flow cytometry, blood samples were blocked in anti-mouse CD16/32 antibody (1:100; BioLegend, 101302) and 10% normal mouse serum (Sigma, M5905) for 10 min at 4°C. Cells were then stained using antibodies described in supplementary table 16 for for 20 minutes at 4°C, washed twice in PEB buffer then resuspended in PEB buffer for analysis. DAPI (1:1000) was added immediately before sample acquisition. All samples were acquired on a six-laser BD LSRFortessa™ Cell Analyzer (BD Biosciences). Data were analysed using FlowJo™ v10.8 (BD Biosciences) Software.

For flow cytometric analysis of blood from bone marrow chimera mice, viable ($DAPI^-$) single CD45.2+ cells were gated. Monocytes were identified as $CD11b^+$ $CD115^+$ cells, having excluded neutrophils ($CD11b^+$ $Ly\text{-}6G^+$), eosinophils (Siglec F^+) and lineage+ (NK1.1, Siglec H, CD19, CD3) cells. The degree of blood monocyte donor chimerism for each mouse was calculated as the proportion of monocytes which were CD45.1+, and this value was used to normalise the degree of donor chimerism for each liver macrophage subpopulation from the same mouse.

For human liver flow cytometry, IHL were stained with a blue fixable live/dead dye (Invitrogen; Thermo Fisher Scientific) for the exclusion of dead cells and doublets. Cell surface markers were stained with saturating concentrations of monoclonal antibodies for 30 min at 4 °C in 50% diluted Brilliant Violet buffer (BD Bioscience). To prevent unwanted binding of antibodies to Fc receptors (FcR), an FcR blocking step (Miltenyi Biotech) was included 20% diluted FcR block, 10 min prior to surface antibody labelling. Once surface-stained cells were fixed using

Cytofix/Cytoperm (BD Bioscience). Intracellular markers were subsequently stained in a 0.1% saponin-based buffer (Sigma-Aldrich; Merck) containing 0.5% FBS for 30 min at 4 °C. Antibodies are summarised in Supplementary Table 17. All samples were acquired in 1x PBS on a 5-laser Cytek Aurora system running SpectraFlow (v.2.2). Data were analysed using FlowJo (v.9.9.4 or v.10.8.1; TreeStar; BD Bioscience).

For FACS sorting for mouse scRNAseq and scATACseq, following blocking the isolated liver and blood cells were incubated with primary antibodies for 20 minutes at 4°C. All antibodies, conjugates, dilutions, and their catalogue numbers used for FACS of hepatic NPCs and blood leukocytes are presented in Supplementary Table 17. After antibody staining, cells were washed with PEB buffer and centrifuged. DAPI viability staining (1:1000) was performed immediately before acquiring the samples on a BD FACSAria™ II (Becton Dickinson). Viable hepatic $CD45^+$ cells and peripheral blood $CD45^+$ Ly-$6G^-$ leukocytes were sorted from healthy and CCl_4-treated mice at stated timepoints following the final CCl_4 injection.

Mouse scRNAseq and scATACseq

ScRNAseq was performed using the Chromium™ Single Cell 3′ Library and Gel Bead Kit v2 (10X Genomics, PN-120237) and the Chromium™ Single Cell A Chip Kit (10X Genomics, PN-120236) on the Chromium™ Single Cell Platform (10X Genomics), according to manufacturer's protocol. After FACS sorting, cells were washed and counted using the Bio-Rad TC20 cell counter (Bio-Rad). Approximately 12,000 cells were loaded on each lane of the chip. For scATACseq, after FACS sorting, cells were washed and nuclei isolated as described in the 10X Genomics demonstrated protocol (document number CG000169), including modifications for low cell input nuclei isolation. Nuclei were counted using a Countess II automated cell counter and processed for scATACseq using the Chromium™ Single Cell ATAC Library & Gel Bead Kit (10X Genomics, PN-1000111) and the Chromium™ Single Cell ATAC Chip E Kit (10X Genomics, PN-1000086), according to the manufacturers protocol. A nuclei recovery of 7000 per lane was targeted. All libraries were sequenced on an Illumina HiSeq 4000.

Immunofluorescence staining

Immunofluorescence staining of human liver was performed on 5 µm paraffin-embedded tissue sections. After rehydration, heat-mediated antigen retrieval was carried out in separate runs: using pH 9 Tris-EDTA buffer (15 minutes) for the primary antibody for OLR1, and pH 6 sodium citrate buffer (15 minutes) for the primary antibody for GPNMB. Sections were then incubated in 3% hydrogen peroxide (Sigma-Aldrich, H1009) for 10 minutes, washed with PBS containing 0.1% Triton-X (PBS-T), and blocked with protein block (Abcam, ab64226) for 30 minutes. Primary antibodies, diluted in antibody diluent (Abcam, ab64211), were incubated overnight at 4 °C. All antibodies are listed in Supplementary Table 17. The following day, sections were washed with PBS-T and incubated with ImmPRESS HRP Polymer Detection Reagents (Vector Laboratories, MP-7402) for 30 minutes at room temperature (RT). After washing with PBS, initial staining was detected using Cy5 tyramide (Perkin-Elmer, NEL741B001KT) for OLR1 and Cy3 tyramide for GPNMB both at a 1:1,000 dilution. Slides were then washed with PBS-T, followed by an additional round of heat-mediated antigen retrieval using pH 6 sodium citrate buffer (15 minutes) for the second antibody CD68. After PBS washes and protein blocking, sections were incubated with CD68 for 1 hour at RT. Detection was performed using ImmPRESS Polymer and fluorescein-conjugated tyramide, as described above. This staining sequence was repeated for a third round using the antibodies COL1A1 or OLR1. Heat retrieval was performed with pH 6 sodium citrate buffer

(15 minutes) for COL1A1 and with pH 9 Tris-EDTA buffer (15 minutes) for OLR1. Both antibodies were incubated overnight at 4 °C, followed by detection with Cy3 tyramide for COL1A1 and Cy5 tyramide for OLR1. After the final staining cycle, all sections were counterstained with DAPI (Sigma-Aldrich, D3571; 1:1,000) and mounted using ProLong Gold antifade reagent (Thermo Fisher Scientific, P36930). Fluorescent images of whole tissue sections were captured using the AxioScan.Z1 slide scanner (Zeiss).

Immunofluorescence staining of stem cell-derived 3D multilineage liver organoids was performed on 4 µm paraffin-embedded tissue sections. After rehydration, heat-mediated antigen retrieval was carried out in Tris-EDTA for 10 minutes. Sections were blocked and stained with primary antibodies as described above. The day after, sections ware stained with fluorescent secondary antibodies for 2 hours. All antibodies, catalogue numbers, lot numbers and dilutions used are in Supplementary Table 17. Sections were DAPI stained and mounted as described above. Images were taken using an Olympus (Olympus Global) microscope. All images were processed and analysed on Zen Blue (Zeiss) software. Thresholds for all the fluorescent channels were set against the negative controls.

Immunofluorescence staining of mouse liver tissue was performed on 4µm paraffin-embedded sections. Sections were dewaxed and rehydrated as described above. Multiplex IF staining was performed sequentially using an automated staining system (Leica Bond), using the Opal fluorophore method. Briefly, after rehydration, heat-mediated antigen retrieval was carried out in Tris-EDTA for 10 minutes. Sections were blocked and stained with primary antibodies for 1hr. Species-specific polymer detection was then applied to the sections, followed by the corresponding Opal fluorophore. Once the first biomarker had been developed, the process was repeated sequentially for the remaining biomarkers. Antibody concentrations and Opals used are detailed in Supplementary Table 17. After multiplex IF staining, all sections were stained with DAPI (Sigma-Aldrich, D3571; 1:1000) and mounted using ProLong Gold antifade reagent (Thermo Fisher Scientific, P36930). Sections were imaged at 40x magnification using the multispectral Vectra Polaris imaging instrument, and images spectrally unmixed using inForm software (v2.7.0). Quantification of IBA1+TREM2+OLR1+ and IBA1+TREM2+OLR1-GPNMB+ cell subpopulations was performed using QuPath image analysis software (version 6). Single marker object classifiers were created, concatenated and subsequently applied to each tissue sample.

Cell culture

THP-1 human monocytes were cultured in RPMI (Gibco, 31870074) containing 1% Pen-strep (Gibco, 11548876), 1% L-glutamine (Gibco, 25030081) and 10% fetal bovine serum (FBS) (Sigma-Aldrich, F9665). THP-1 monocytes were seeded at a density of 1.14 x 10^5 cells/cm^2 and differentiated into macrophages after treatment with 100 nM PMA (Sigma-Aldrich, P1585).

For human primary peripheral blood mononuclear cells (PBMC) isolation, SepMate™-50 IVD (STEMCELL Technologies, 85450) kit was used, according to manufacturer's instructions. In brief, 15 mL of Lymphoprep™ (STEMCELL Technologies, 07801) was added to a SepMate™ tube. An equal volume of patients' blood was diluted in PBS containing 2% FBS (Gibco, A5256701) and added to the SepMate™ tube. After centrifugation (1200 g, 15 min, RT), the supernatant was collected and washed with PBS with 2% FBS. After centrifugation (300 g, 8 min, 4°C), the PBMC pellet was resuspend in PBS with 2% FBS.

For the enrichment of human primary monocytes, EasySep™ Human Monocyte Enrichment Kit (STEMCELL Technologies, 19059) was used, following manufacturer's instructions. In brief, tetrameric antibody complexes enrichment cocktail was added to the PBMC suspension and incubated for 5 min at RT. Next, magnetic particles were mixed with the sample and incubated for 5 min at RT. The tube was inserted into EasyEights™ EasySep™ magnet (STEMCELL Technologies, 18103) and incubated for 2.5 min at RT. Primary monocytes were negatively isolated and cryopreserved using CryoStor CS10 (STEMCELL Technologies, 100-1061) in liquid nitrogen. For differentiation of patient-derived monocytes into macrophages, cells were thawed and seeded at a density of 3.13 x 105 cells/cm^2 in the same media as THP-1 cells with the addition of 10 mM HEPES (Gibco, 15630080) and 50 ng/mL human M-CSF (Peprotech, 300-25).
For comparison between macrophage polarization conditions, patient-derived monocytes were either differentiated with 50 ng/mL GM-CSF (Peprotech, 17850053) for 4 days and treated with TPP cocktail for 48 hours or differentiated with 50 ng/mL M-CSF (Peprotech, 300-25) for 5 days and treated with 150 µM palmitic acid (MedChemExpress, 57-10-3) for 24 hours. TPP cocktail included 800 U/mL TNF-α (Peprotech, 17840043), 1 µg/mL PGE2 (Sigma-Aldrich, P0409) and 1 µg/mL Pam3CSK4 (InvivoGen, tlrl-pm2s-1). For the collection of supernatants to be used in conditioned media experiments on HSCs, cells were washed with PBS and subsequently incubated in fresh media in the absence of activation stimuli for an additional 24 hours.

LX2 human hepatic stellate cells were cultured in DMEM high-glucose with 1% L-glutamine (Sigma-Aldrich, D5796) containing 1% Pen-strep (Gibco, 11548876), and 10% fetal bovine serum (FBS) (Sigma-Aldrich, F9665).

Cell lines and primary cells were cultured in incubators at 37 °C with 5% CO_2 levels and humidified conditions.

OLR1 knockdown/inhibition in macrophages

For gene knockdown in THP-1 cells, OLR1 Silencer™ Select Validated siRNA (Ambion, 4390824) or Silencer™ Select Negative Control No. 1 siRNA (Ambion, 4390843) was delivered via TransIT-X2™ Dynamic Delivery System (Mirus, MIR 6003) 4 days after PMA-induced differentiation, according to the manufacturer's manual. Media was changed the day after, and RNA/supernatant collected on day 7. Gene knockdown in patient MDMs was performed 5 days after differentiation of primary monocytes, as described above. RNA/supernatants were collected on day 8. *OLR1* knockdown efficiency was assessed by RT–qPCR. OLR1 inhibition in patient-derived MDMs was performed by adding the human OLR1 Blocking Antibody Golocdacimab/MEDI6570 (MedChemExpress, HY-P99646) or human IgG1 lambda1 Isotype Antibody (MedChemExpress, HY-P99992) for 48 hours before supernatant collection. All supernatants were centrifuged (1000 g, 10 min) to remove debris and used subsequently for ELISA or conditioned media stimulation assays.

Hepatic stellate cell stimulation assay

For conditioned media experiments, LX2 cells were seeded at a density of 3.13 x 10^4 cells/cm^2. The following day, cells were serum-starved for 24 hours. On day 3, the supernatant from primary macrophages was mixed with serum-free HSC media at a ratio of 1/4 and used to treat LX2 cells. On day 4, RNA extraction or proliferation assays were performed.

Stem cell-derived 3D multilineage liver organoid

Human H9 Embryonic Stem Cells (p46) were used in collaboration with Prof. David Hay's Lab. To generate 3D multilineage liver organoids, pluripotent stem cells were differentiated into hepatocytes, hepatic stellate cells and endothelial cells and aggregated at a ratio of 10: 3: 1 (cell number 3000:900:300) in 50uL per microwell in Gri3D 96-well plates (Sun Bioscience, Gri3D-96P-S-96-500), according to a previously published protocol[2–4]. Human primary monocytes were added at a ratio of 5 (cell number 1500) in 50uL per microwell to spheroids 1 day after cell aggregation. CSF-1 (Peprotech, 300-25-10UG) prepared at a stock concentration of 10ug/mL in PBS and used at 50ng/mL, was added to the spheroid culture media and 150uL media changed was carried out every 48 hours. For optional drug treatment, Pexidartinib hydrochloride (PLX-3397, MedChemExpress) or A83-01 (Tocris Bioscience, 2939/10) were prepared at a stock concentration of 5mM in DMSO and they used at final concentration of 1 uM in spheroid culture media between day 7 and 14 post-monocyte addition. Vehicle (DMSO only) was used as a control. Spheroids were harvested for RNA extraction or immunofluorescence staining.

RNA extraction and RT-qPCR

Cell lysis and RNA extraction were performed using PureLink™ RNA Mini Kit (Invitrogen, 12183018A) or RNAqueous™-Micro Total RNA Isolation Kit (Thermo Fisher Scientific, AM1931), according to manufacturer's instructions. cDNA was synthesized using SuperScript™ IV Reverse Transcriptase (Invitrogen, 1809005) following manufacturer's manual. Quantitative PCR was conducted using LightCycler™ 480 SYBR Green I Master (Roche, 04707516001) either on QuantStudio™ 5 Real-Time PCR System (Applied Biosystems) or LightCycler™ 480 Instrument II (Roche). The $2^{-\Delta\Delta Ct}$ quantification method was used to analyse gene mRNA expression. B-ACTIN or GAPDH were used as housekeeping genes for normalisation. Sample gene expression was calculated relative to the average mRNA expression of control groups. For primary human liver cell 3D multilineage spheroids, lysis and RNA extraction were performed using the Zymo Quick-RNA Microprep kit (Zymo research) and cDNA was synthesized using SuperScript IV Reverse Transcriptase (Invitrogen, 1809005). Quantitative PCR was conducted using PowerUp SYBR Green Master Mix (Thermofisher, A25742). Relative RNA expression levels were calculated using the ΔΔCt quantification method. GAPDH was used as reference gene for normalization as previously described for primary liver cultures[14]. Primers are listed in Supplementary Table 18.

ELISA

For cytokine expression quantification, LEGENDplex™ assay (BioLegend, 740809) was performed on the supernatant of THP-1 cells, according to manufacturer's instructions. Fluorescence was acquired on a NovoCyte flow cytometer (Agilent). Data were analysed using the BioLegend's LEGENDplex™ (BioLegend) data analysis software. Procollagen Ia1 levels were measured in culture supernatants using the Human Pro-collagen I alpha 1 DuoSet ELISA (R&D Systems; DY6220-05) following the manufacturer's instructions.

MTT proliferation assay

To assess cell viability and proliferation, the Cell Proliferation Kit I MTT (Roche, 11465007001) was used, according to manufacturer's instructions. Absorbance was acquired on a BioTek Synergy HTX Multimode Reader (Agilent) at 570 nm. Background signal was subtracted to the absorbance of each sample. Cell proliferation was calculated relative to the average absorbance of control groups.

Mouse scATACseq analysis

Analysis was performed on 8 mouse scRNAseq datasets (n=4 CD45+ cells from liver and n=4 CD45+ Ly-6G- cells from peripheral blood) in total. Blood and liver cells were obtained from chronic CCl_4 treated mice at two timepoints following the final CCl_4 injection (24 hours and 4 weeks) and age-matched uninjured controls. Sequence alignment and initial sample processing was performed using Cell Ranger ATAC (v.1.1.0[15]) and the mm10 reference mouse genome (version cellranger-atac-mm10-1.1.0 downloaded from 10X Genomics). Gene annotations for the mouse genome (mm10) were retrieved from the EnsDb.Mmusculus.v79 (v.2.99.0) database. Subsequent preprocessing, clustering, annotation, motif discovery, and downstream analysis was performed in R (v.4.2.2) using the R packages Seurat (v.4.1.3) and Signac (v.1.14.0[16]) Cells failing quality control thresholds were removed (reads in peaks <300 or >50,000; fraction of reads in called peak regions (FRiP) <0.05; blacklist ratio >0.125; transcription start site (TSS) enrichment <2; number of detected features and counts <3000). After quality control, samples were integrated using Harmony (v.1.0.0), creating a single dataset with 29,272 cells.

Normalization and dimension reduction was performed using Latent Semantic Indexing (LSI), as described by Cusanovish et al[17]. Clustering was performed using LSI components 2 to 30 to construct the KNN graph, and a resolution of 3.5, to partition the dataset into 16 final clusters. LSI component 1 was omitted from the analysis due to its correlation with sequencing depth.

Cluster Identity with scRNAseq and Peak Calling

Using cross-modality integration and label transfer[18], scATACseq cells were annotated using the mouse CCl_4 scRNAseq dataset. First, anchors were identified between the query (scATACseq) and reference (scRNAseq) datasets using canonical correlation analysis (CCA), followed by the transfer of annotations from the reference to query dataset. MPs were subset (14,613 cells) and subsequent processing was performed using R package, ArchR (v.1.0.2[19]) and the precompiled mm10 genome (BSgenome.Mmusculus.UCSC.mm10). Peak calling for MPs was performed using python package MACS2 (v.3.0.3[20]) installed in R via the Anaconda package repository using R package Herper (v.1.18.0[21])

After subsetting, the top abundant peak features were recalculated and used as input for further normalization and clustering. As before, cells failing quality control thresholds were removed (reads in peaks <300 or >50,000; fraction of reads in called peak regions (FRiP) <0.05; blacklist ratio >0.125; transcription start site (TSS) enrichment <2; number of detected features and counts <3000). TF-IDF normalization was performed, followed by LSI dimension reduction. Samples were then reintegrated using Harmony implemented within ArchR.

Gene scores were calculated, providing an estimation of how highly expressed a gene will be based on the accessibility of local regulatory elements. Label transfer was then performed using the MP scRNA-seq dataset as reference.

Differential Peaks and TF Motif Enrichment

Cluster specific peaks were identified using the ArchR function '*addMarkerFeatures*' to perform pairwise Wilcoxon Rank Sum tests. By setting the 'bias' parameter to adjust for TSS enrichment and total unique fragments per cell, differences in quality between clusters are adjusted for during testing. Differential peaks were identified between the MP clusters SAMac1 and SAMac2. Using the chromVARmotifs R package (v.1.30.0[22]), motif sets from the CIS-BP repository[23] were retrieved. To identify motifs that are enriched in SAMac1 versus SAMac2, differential peaks with false discovery rate (FDR) <= 0.1 and log2 adjusted fold change (log2FC) >= 0.5 were subset and tested for motif enrichment.

Supplementary Figures

Supplementary Figure 1: Integration and lineage annotation of human liver scRNAseq data. (**A**) UMAP of integrated scRNAseq data (843,457 cells; 77 patients; 7 studies) coloured and labelled by cell lineage annotation. (**B**) Dotplot showing mean expression of marker genes and percentage of cells expressing them for each lineage cell type. (**C**) UMAP of integrated scRNAseq data split by original study. Coloured and labelled by cell lineage annotation.

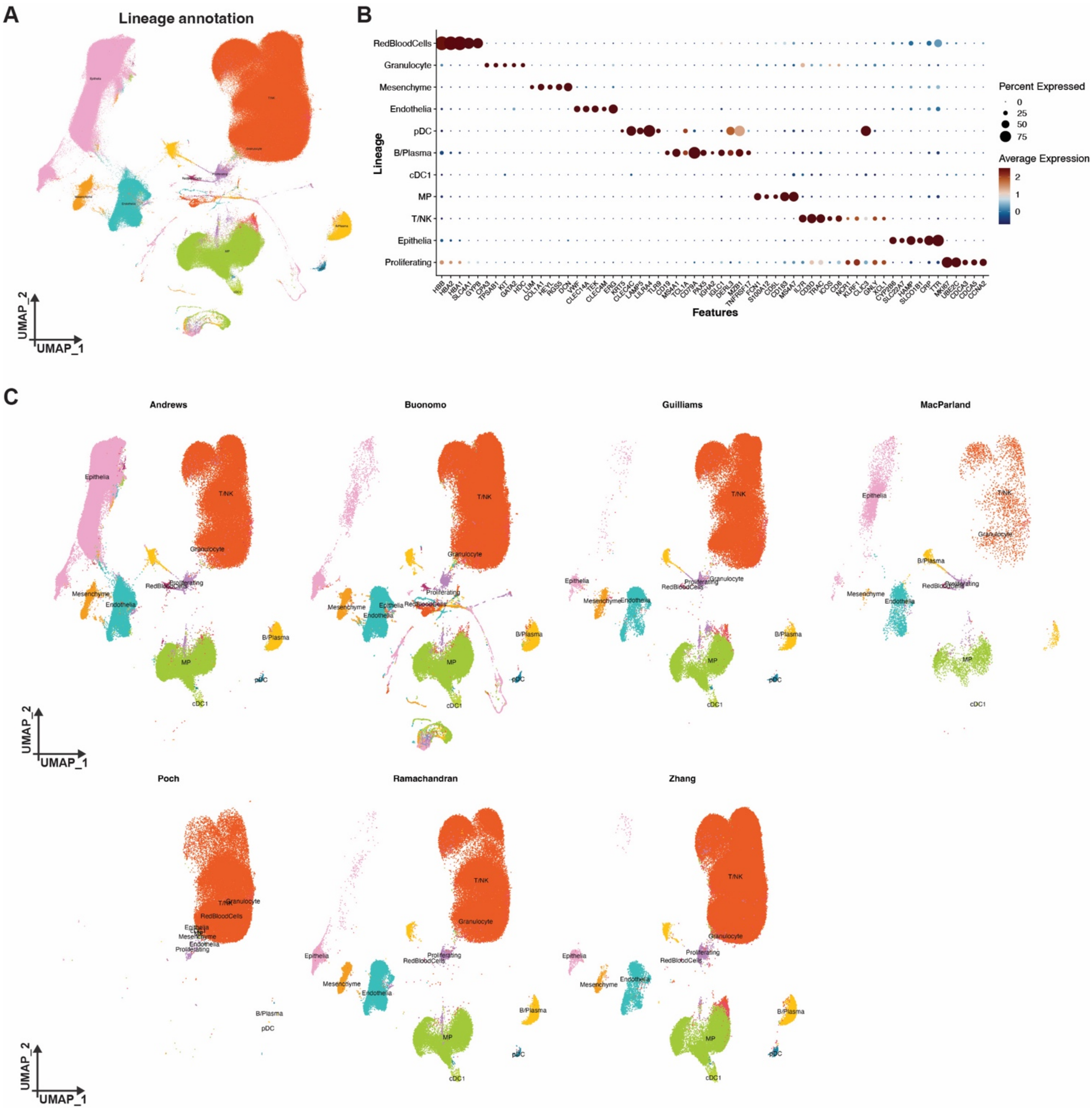

Supplementary Figure 2: Hierarchical clustering of human liver cell states. (**A**-**C**) Plots of transcriptional similarity between stated cell types. Colour scale indicates log_{10}-transformed pairwise modularity ratios between clusters. (**A**) Similarity plot for annotated level 1 cell types in scRNAseq atlas. (**B**) Similarity plot for annotated level 2 cell types in scRNAseq atlas. (**C**) Similarity plot for annotated level 3 cell types in scRNAseq atlas.

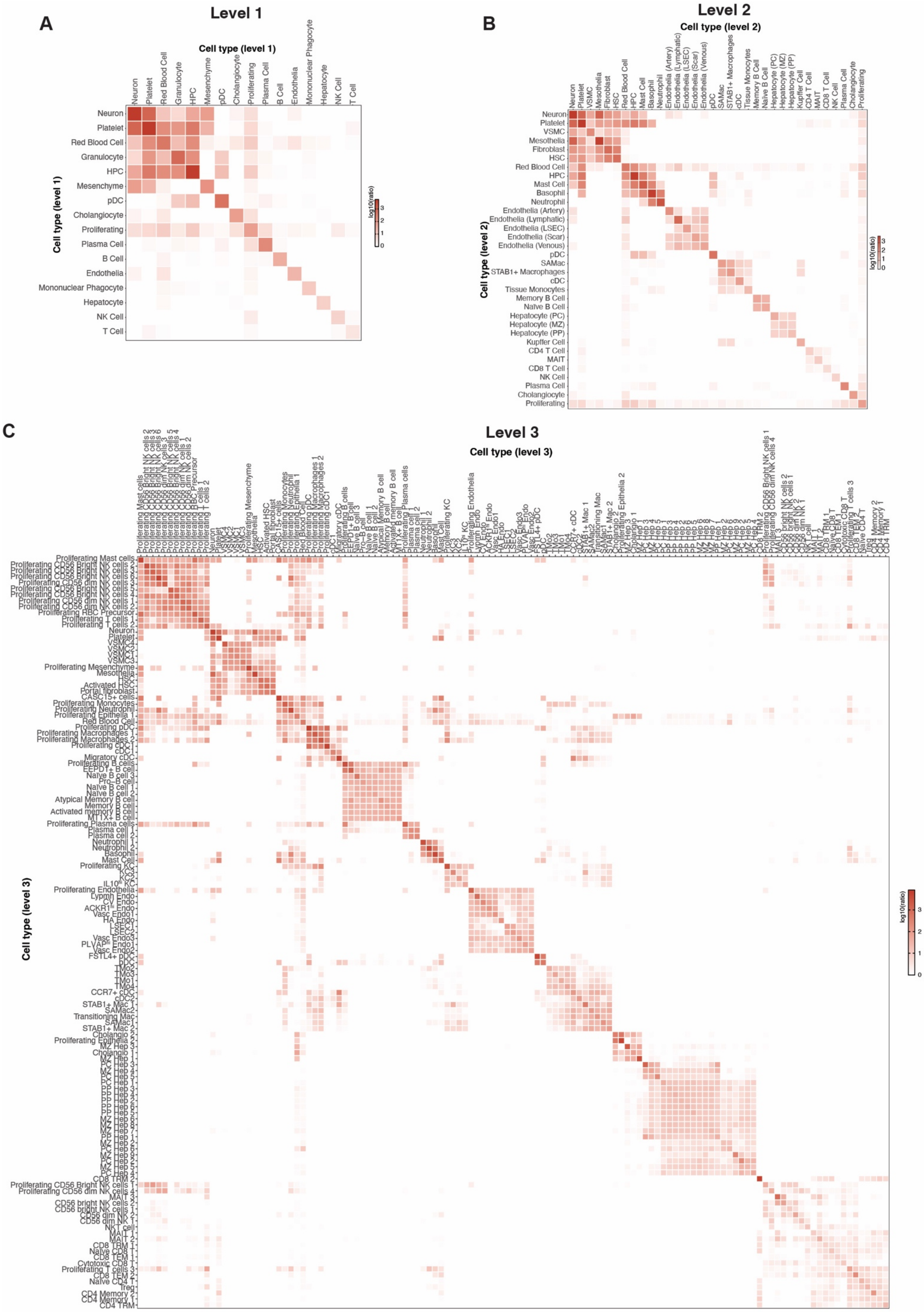
A
Level 1
Cell type (level 1)
Neuron
Platelet
Red Blood Cell
Granulocyte
HPC
Mesenchyme
pDC
Cholangiocyte
Proliferating
Plasma Cell
B Cell
Endothelia
Mononuclear Phagocyte
Hepatocyte
NK Cell
T Cell
log10(ratio)
0
1
2
3
B
Level 2
Cell type (level 2)
Neuron
Platelet
VSMC
Mesothelia
Fibroblast
HSC
Red Blood Cell
HPC
Mast Cell
Basophil
Neutrophil
Endothelia (Artery)
Endothelia (Lymphatic)
Endothelia (LSEC)
Endothelia (Scar)
Endothelia (Venous)
pDC
SAMac
STAB1+ Macrophages
cDC
Tissue Monocytes
Memory B Cell
Naïve B Cell
Hepatocyte (PC)
Hepatocyte (MZ)
Hepatocyte (PP)
Kupffer Cell
CD4 T Cell
MAIT
CD8 T Cell
NK Cell
Plasma Cell
Cholangiocyte
Proliferating
log10(ratio)
0
1
2
3
C
Level 3
Cell type (level 3)
log10(ratio)
0
1
2
3

Supplementary Figure 3: Targeting OLR1+ MDMs Abrogates Fibrosis in Human Liver Spheroids

(**A**) Workflow for generating and drug treating embryonic stem (ES) cell-derived spheroids containing human monocyte-derived macrophages (MDMs) from CLD patients. Created in BioRender. Colella, F. (2025) https://BioRender.com/sp1zyj3. (**B**) Representative immunofluorescence of a D8 liver spheroid following monocyte addition; markers IBA1 (red), VCAM1 (green) and DAPI (blue). Scale bar=20μm. Images representative of 3 spheroid samples. (**C**) qPCR of D8 spheroids with and without addition of patient monocytes (n=8 patients). Expression of stated gene in each spheroid culture relative to expression of control (no monocyte) group. *COL1A1* p=0.0469; *OLR1* p=0.0156; *CD68* p=0.0312; *SPP1* p=0.0156. (**D**) qPCR of D15 spheroids comparing treatment with TGFβ inhibitor (A83-01) vs. vehicle (n=7 per group). Expression of *COL1A1* and *COL3A1* relative to mean expression of vehicle group . *COL1A1* p=0.0156; *COL3A1* p=0.0156. (**E**) qPCR of D15 spheroids comparing treatment with CSF1R inhibitor (Pexidartinib) vs. vehicle (n=8 per group for *IL1B*; n=7 per group for other genes). Expression of stated gene relative to mean expression of vehicle group. *COL1A1* p=0.0156; *COL3A1* p=0.0156; *OLR1* p=0.0469; *CD68* p=0.0156; *SPP1* p=0.0156; *IL1B* p=0.0156; *TTR* p=0.0312. **C**-**E,** Dots represent biological replicates with monocytes from different CLD patients. Bars indicate mean ± S.E.M. Two-sided Wilcoxon matched-pairs signed rank test performed between groups for each gene; *p<0.05.

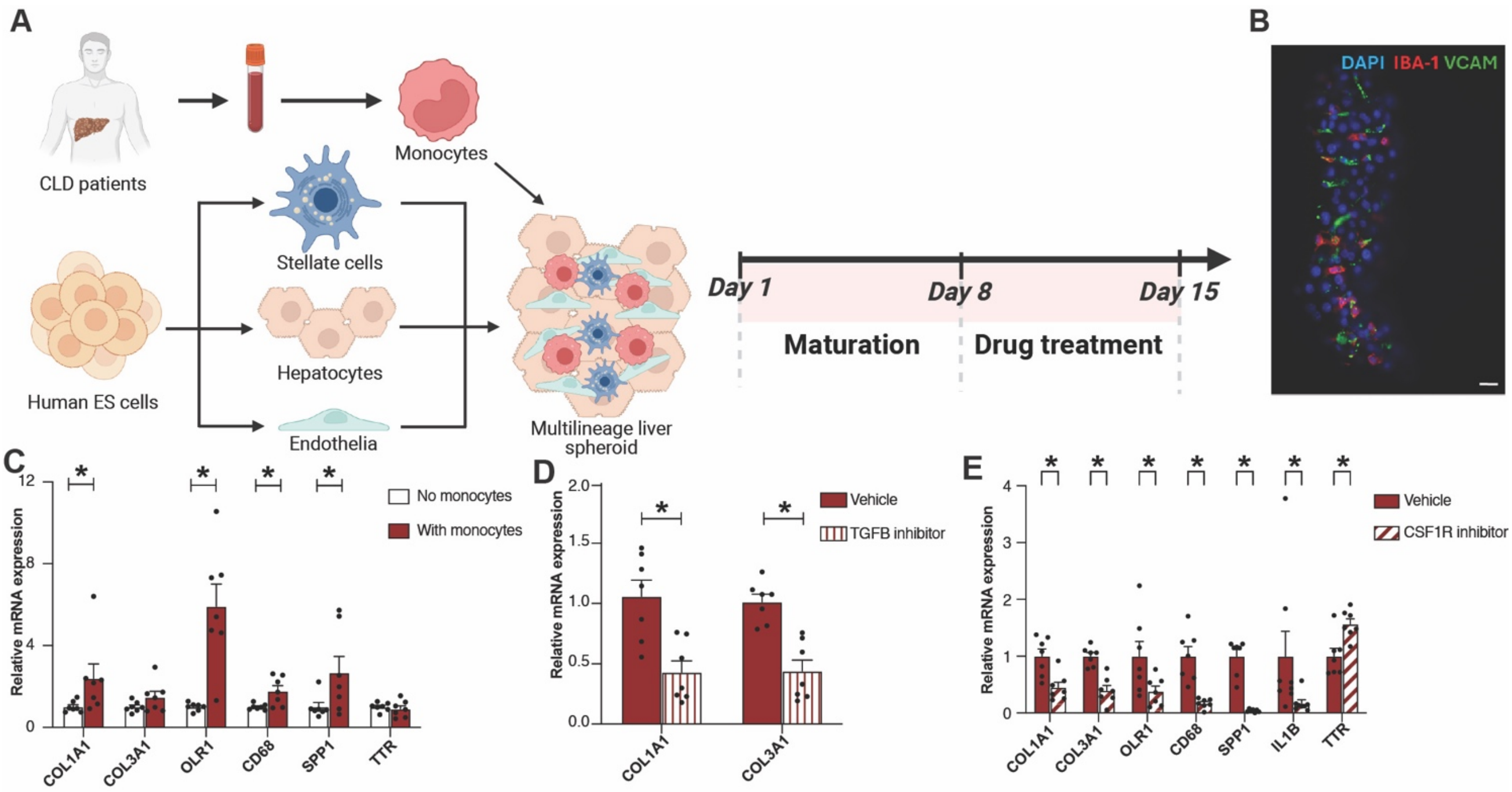
A
CLD patients
Monocytes
Stellate cells
Human ES cells
Hepatocytes
Endothelia
Multilineage liver spheroid
Day 1
Day 8
Day 15
Maturation
Drug treatment
B
DAPI IBA-1 VCAM
C
No monocytes
With monocytes
Relative mRNA expression
COL1A1
COL3A1
OLR1
CD68
SPP1
TTR
D
Vehicle
TGFB inhibitor
Relative mRNA expression
COL1A1
COL3A1
E
Vehicle
CSF1R inhibitor
Relative mRNA expression
COL1A1
COL3A1
OLR1
CD68
SPP1
IL1B
TTR

Supplementary References

1. Peake, K. *et al.* Busulfan as a myelosuppressive agent for generating stable high-level bone marrow chimerism in mice. *J. Vis. Exp.* e52553 (2015) doi:10.3791/52553.
2. Meseguer-Ripolles, J., Kasarinaite, A., Lucendo-Villarin, B. & Hay, D. C. Protocol for automated production of human stem cell derived liver spheres. *STAR Protoc.* **2**, 100502 (2021).
3. Lucendo-Villarin, B. *et al.* Development of a cost-effective automated platform to produce human liver spheroids for basic and applied research. *Biofabrication* **13**, 015009 (2020).
4. Kasarinaite, A. *et al.* Hormone correction of dysfunctional metabolic gene expression in stem cell-derived liver tissue. *Stem Cell Res. Ther.* **16**, 130 (2025).
5. Gieling, R. G., Wallace, K. & Han, Y.-P. Interleukin-1 participates in the progression from liver injury to fibrosis. *American Journal of Physiology-Gastrointestinal and Liver Physiology* **296**, G1324–G1331 (2009).
6. Alexanian, M. *et al.* Chromatin remodelling drives immune cell-fibroblast communication in heart failure. *Nature* **635**, 434–443 (2024).
7. Amrute, J. M. *et al.* Targeting immune–fibroblast cell communication in heart failure. *Nature* **635**, 423–433 (2024).
8. Bhattacharya, M. & Ramachandran, P. Immunology of human fibrosis. *Nat. Immunol.* **24**, 1423–1433 (2023).
9. Murdocca, M. *et al.* LOX-1 and cancer: an indissoluble liaison. *Cancer Gene Ther.* **28**, 1088–1098 (2021).
10. Barreto, J., Karathanasis, S. K., Remaley, A. & Sposito, A. C. Role of LOX-1 (Lectin-Like Oxidized Low-Density Lipoprotein Receptor 1) as a Cardiovascular Risk Predictor. *Arterioscler. Thromb. Vasc. Biol.* **41**, 153–166 (2021).
11. Ozturk, O. *et al.* Increased serum soluble lectin-like oxidized low-density lipoprotein receptor-1 levels in patients with biopsy-proven nonalcoholic fatty liver disease. *World J. Gastroenterol.* **21**, 8096–8102 (2015).
12. Ho, C.-M. *et al.* Accumulation of free cholesterol and oxidized low-density lipoprotein is associated with portal inflammation and fibrosis in nonalcoholic fatty liver disease. *J. Inflamm.* **16**, 7 (2019).
13. Yoshimoto, R. *et al.* The discovery of LOX-1, its ligands and clinical significance. *Cardiovasc. Drugs Ther.* **25**, 379–391 (2011).
14. Wesseler, M. F. *et al.* 3D microperfusion of mesoscale human microphysiological liver models improves functionality and recapitulates hepatic zonation. *Acta Biomater.* **171**, 336–349 (2023).
15. Satpathy, A. T. *et al.* Massively parallel single-cell chromatin landscapes of human immune cell development and intratumoral T cell exhaustion. *Nat. Biotechnol.* **37**, 925–936 (2019).
16. Stuart, T., Srivastava, A., Madad, S., Lareau, C. A. & Satija, R. Single-cell chromatin state analysis with Signac. *Nat. Methods* **18**, 1333–1341 (2021).
17. Cusanovich, D. A. *et al.* Multiplex single-cell profiling of chromatin accessibility by combinatorial cellular indexing. *Science (1979).* **348**, 910–914 (2015).
18. Stuart, T. *et al.* Comprehensive Integration of Single-Cell Data. *Cell* **177**, 1888-1902.e21 (2019).
19. Granja, J. M. *et al.* ArchR is a scalable software package for integrative single-cell chromatin accessibility analysis. *Nat. Genet.* **53**, 403–411 (2021).
20. Gaspar, J. M. Improved peak-calling with MACS2. *bioRxiv* 496521 (2018) doi:10.1101/496521.

21. Paul, M., Carroll, T. & Barrows, D. Herper: The Herper package is a simple toolset to install and manage conda packages and environments from R. *R package version 1.18.0* https://doi.org/10.18129/B9.bioc.Herper (2025) doi:10.18129/B9.bioc.Herper.
22. Schep, A. N., Wu, B., Buenrostro, J. D. & Greenleaf, W. J. chromVAR: inferring transcription-factor-associated accessibility from single-cell epigenomic data. *Nat. Methods* **14**, 975–978 (2017).
23. Weirauch, M. T. *et al.* Determination and Inference of Eukaryotic Transcription Factor Sequence Specificity. *Cell* **158**, 1431–1443 (2014).
