# Supplementary material for: A human single-cell atlas identifies OLR1+ scar-associated macrophages as a potential therapeutic target for chronic liver disease
Source: Nat Genet. 2026 Sep 28;58(10):2550–65. doi: 10.1038/s41588-026-02774-w (PMC13649586; doi:10.1038/s41588-026-02774-w)
Supplement: Supplementary file 2 [file 41588_2026_2774_MOESM2_ESM.pdf]

nature portfolio

Corresponding author(s): Prakash Ramachandran

Last updated by author(s): Aug 4, 2026

Reporting Summary

Nature Portfolio wishes to improve the reproducibility of the work that we publish. This form provides structure for consistency and transparency in reporting. For further information on Nature Portfolio policies, see our Editorial Policies and the Editorial Policy Checklist.

Statistics

For all statistical analyses, confirm that the following items are present in the figure legend, table legend, main text, or Methods section.

n/a	Confirmed	
☐	☒	The exact sample size (*n*) for each experimental group/condition, given as a discrete number and unit of measurement
☐	☒	A statement on whether measurements were taken from distinct samples or whether the same sample was measured repeatedly
☐	☒	The statistical test(s) used AND whether they are one- or two-sided<br>*Only common tests should be described solely by name; describe more complex techniques in the Methods section.*
☐	☒	A description of all covariates tested
☐	☒	A description of any assumptions or corrections, such as tests of normality and adjustment for multiple comparisons
☐	☒	A full description of the statistical parameters including central tendency (e.g. means) or other basic estimates (e.g. regression coefficient) AND variation (e.g. standard deviation) or associated estimates of uncertainty (e.g. confidence intervals)
☐	☒	For null hypothesis testing, the test statistic (e.g. *F*, *t*, *r*) with confidence intervals, effect sizes, degrees of freedom and *P* value noted<br>*Give P values as exact values whenever suitable.*
☒	☐	For Bayesian analysis, information on the choice of priors and Markov chain Monte Carlo settings
☒	☐	For hierarchical and complex designs, identification of the appropriate level for tests and full reporting of outcomes
☐	☒	Estimates of effect sizes (e.g. Cohen's *d*, Pearson's *r*), indicating how they were calculated

Our web collection on statistics for biologists contains articles on many of the points above.

Software and code

Policy information about availability of computer code

Data collection	No software was used for data collection
Data analysis	Data analysis Code for the analysis is available at Gitlab (https://git.ecdf.ed.ac.uk/ramachandran-lab/olr1-paper).<br>Initial processing of single-cell RNAseq and ATACseq was performed on the Edinburgh Compute and Data Facility (ECDF) Linux Compute Cluster, using Altair Gridengine batch system on Rocky Linux 9.<br>10X Genomics Cell Ranger versions v.7.0.0 and v.3.0.2 were used to process human and mouse scRNAseq data, respectively. Mouse scATACseq was processed using 10X Genomics Cell Ranger ATAC (v.1.1.0).<br>Analysis and visualisation of human scRNAseq was performed locally in RStudio (v. 2023.09.1+494) running R (v.4.3.2), using the following R packages: Seurat (v.5.0.0), ggplot2 (v.3.5.1), grid (v.4.3.2), DecontX (v.1.14.2), scran (v.1.30.2), bluster (v.1.12.0), ShinyCell (v.2.1.0), loupeR (v.1.1.4), MiloR (v.1.8.1) and ggpubr (v0.6.0).<br>Additional human analysis was performed locally using python (v.3.10.16), and the following python packages: liana-py (v.1.4.0), cell2cell (v.0.7.4), sceasy (v.0.0.7), reticulate (v.1.35.0).<br>Analysis and visualisation of mouse scRNAseq was performed locally in RStudio (v2023.03.0+386) running R (v.4.2.2), using the following R packages: Seurat (v.4.1.2), ggplot2 (v.3.2.1) and grid (v.3.6.3).<br>Analysis of mouse scRNAseq was performed locally in RStudio (v 2023.03.0+386) running R (v.4.2.2), using the following R packages: Seurat (v.4.1.3), Signac (v.1.14.0), Harmony (v.1.0.0), ArchR (v.1.0.2), chromVARmotifs (v.1.30.0) and python package MACS2 (v.3.0.3) installed in R via the Anaconda package repository using R package Herper (v.1.18.0 8).<br>Analysis of human liver needle biopsies was performed locally in RStudio (v 2023.03.0+386) using R (v.4.4.1) and the following R packages: Seurat (v.5.3.1.0000), ggplot2 (v.3.5.2), Harmony (v.1.2.3).

For manuscripts utilizing custom algorithms or software that are central to the research but not yet described in published literature, software must be made available to editors and reviewers. We strongly encourage code deposition in a community repository (e.g. GitHub). See the Nature Portfolio guidelines for submitting code & software for further information.

Data

Policy information about availability of data

All manuscripts must include a data availability statement. This statement should provide the following information, where applicable:

- Accession codes, unique identifiers, or web links for publicly available datasets
- A description of any restrictions on data availability
- For clinical datasets or third party data, please ensure that the statement adheres to our policy

Mouse single cell RNAseq, scATACseq data and human liver biospy scRNAseq data were deposited in the Gene Expression Omnibus (GEO) database under the accession number: GSE298719.
Human single cell RNAseq data were downloaded from the European Nucleotide Archive under accession numbers: GSE243981 , GSE168933 , GSE192742 , GSE115469 , E-MTAB-101430, GSE136103 , GSE181483 and GSE129933.
Reference genomes were downloaded from 10X Genomics, human: GRCh38-2020-A, mouse: mm10-3.0.0 and mouse-ATAC: cellranger-atac-mm10-1.1.0. Gene annotations for the mouse genome (mm10) were retrieved from the EnsDb.Mmusculus.v79 (v.2.99.0) database. Additional analysis of the mouse scATACseq data utilised the UCSC version mm10 mouse genome, based on GRCm38.p6, available from the R package BSgenome.Mmusculus.UCSC.mm10 (v.1.4.3) precompiled mouse genome.
An online data browser for annotated human scRNAseq data is available at https://shiny.igc.ed.ac.uk/Human_Liver_scRNAseq_Atlasv2/. A publicly accessible browser for mouse MP data is available at: https://shiny.igc.ed.ac.uk/mouseMPshinyApp/. Loupe browser compatible files and annotated Seurat objects for human atlas and mouse MP scRNAseq data are available at https://doi.org/10.7488/ds/8163 and https://doi.org/10.7488/ds/8165 respectively.

Research involving human participants, their data, or biological material

Policy information about studies with human participants or human data. See also policy information about sex, gender (identity/presentation), and sexual orientation and race, ethnicity and racism.

Reporting on sex and gender	Biological sex was determined based on clinical metadata where available. For samples lacking reported sex, sex was inferred from single-cell transcriptomic data by assessing the expression of 20 Y chromosome–specific genes, as described in the Methods. Sex was included as a variable in differential abundance and gene expression analyses.
Reporting on race, ethnicity, or other socially relevant groupings	Ethnicity was not included as a covariate.
Population characteristics	Patients were deinfed according to disease stage (healthy vs chronic liver disease) and disease aetiology where available. Attributes of each patient are listed in Supplemental Table 1.
Recruitment	CLD patients were recruited during attendance at the Scottish Liver Transplant Unit, Royal Infirmary of Edinburgh, United Kingdom
Ethics oversight	Local approval for procuring blood samples from CLD patients for in vitro analyses was obtained from the Lothian NRS BioResource and Tissue Governance Unit (study number SR574), following review at the East of Scotland Research Ethics Service (reference 15/ES/0094). All subjects provided written informed consent. For histological assessment of MASLD biopsies, anonymized unstained formalin-fixed paraffin-embedded liver biopsy sections were provided by the Lothian NRS Human Annotated Bioresource (study number SR848) under authority from the East of Scotland Research Ethics Service REC 1, reference 15/ES/0094. Unified transparent approval for the pan-Scotland SteatoSITE project2 was provided by the West of Scotland Research Ethics Committee 4 (Reference: 20/WS/0002), Public Benefit and Privacy Panel for Health and Social Care (PBPP; Reference: 1819-0091), Institutional Research & Development departments and Caldicott Guardians. Liver tissue samples used in for human flow were obtained through the Tissue Access for Patient Benefit Initiative (TAPB) at The Royal Free Hospital (RFH; approved by the UCL-Royal Free Hospital BioBank Ethical Review Committee references: 11/WA/0077, 16/WA/0289 and 21/WA/0388). For flow cytometry analysis of human liver macrophages, liver tissue samples were obtained through the Tissue Access for Patient Benefit Initiative (TAPB) at The Royal Free Hospital (RFH; approved by the UCL-Royal Free Hospital BioBank Ethical Review Committee references: 11/WA/0077, 16/WA/0289 and 21/WA/0388).

Note that full information on the approval of the study protocol must also be provided in the manuscript.

Field-specific reporting

Please select the one below that is the best fit for your research. If you are not sure, read the appropriate sections before making your selection.

☒ Life sciences ☐ Behavioural & social sciences ☐ Ecological, evolutionary & environmental sciences

For a reference copy of the document with all sections, see nature.com/documents/nr-reporting-summary-flat.pdf

Life sciences study design

All studies must disclose on these points even when the disclosure is negative.

Sample size	No statistical method was used to predetermine sample size and experimental group sizes were based on prior literature and tissue/sample

Sample size	availability for human analyses. Publicly available Human scRNAseq datasets were selected after thorough review of the literature, and all samples passing our quality control metrics were retained.
Data exclusions	Individual cells from scRNAseq were filtered by thresholding on mitochondrial gene percentage and total unique features. Samples with fewer than 500 cells passing these thresholds were excluded. After clustering, subsequent manual filtering was performed to remove clusters of cells expressing multiple lineage markers (doublets) and low-quality cells with low median unique features. Further details are included in the Methods and provided in the code.
Replication	All findings were reproduced in independent samples, cohorts or experiments. Reproducibility of pertinent findings was also assessed using orthogonal experimental approaches (e.g. separate transcriptomic cohorts, spatial proteomics, cross-species validation)
Randomization	Mice were randomly allocated to experiemental groups. No randomization was conducted for human experiments.
Blinding	Pathology staging of liver biopsies was performed in a blinded manner. Otherwise researchers performing experiments and analysing data were not blinded.

Reporting for specific materials, systems and methods

We require information from authors about some types of materials, experimental systems and methods used in many studies. Here, indicate whether each material, system or method listed is relevant to your study. If you are not sure if a list item applies to your research, read the appropriate section before selecting a response.

Materials & experimental systems

n/a	Involved in the study
☐	☒ Antibodies
☐	☒ Eukaryotic cell lines
☒	☐ Palaeontology and archaeology
☐	☒ Animals and other organisms
☒	☐ Clinical data
☒	☐ Dual use research of concern
☒	☐ Plants

Methods

n/a	Involved in the study
☒	☐ ChIP-seq
☐	☒ Flow cytometry
☒	☐ MRI-based neuroimaging

Antibodies

Antibodies used

All antibodies were validated by the manufacturer or referenced in published studies. When such information were not available, we validated the antibodies using the respective isotype controls. The information below were provided by the suppliers:
CD11b, BUV737, BD Biosciences, Cat: 564443, Clone: M1/70, 1:200.
CD11c, BV421, BioLegend, Cat: 117329, Clone: N418, 1:100.
CD45, BV510, BioLegend, Cat: 103138, Clone: 30-F11, 1:200.
CD45, BV785, BioLegend, Cat: 109839, Clone: 104, 1:100.
CD45.1, PEDazzle 594, BioLegend, Cat: 110748, Clone: A20, 1:100.
CD45.2, BV785, BioLegend, Cat: 109839, Clone: 104, 1:100.
Ly-6C, BV605, BioLegend, Cat: 128035, Clone: HK1.4, 1:200.
CD24, BV510, BioLegend, Cat: 101831, Clone: M1/69, 1:100.
Siglec F, Biotin, BD Biosciences, Cat: 567600, Clone: E50-2440, 1:50.
CD3, Biotin, BioLegend, Cat: 100244, Clone: 17A2, 1:50.
CD3, eflour450, Invitrogen, Cat: 48-0032-82, Clone: 17A2, 1:100.
CD19, eflour450, Invitrogen, Cat: 48-0193-82, Clone: eBio1D3 (1D3), 1:100.
Nk1.1, eflour450, Invitrogen, Cat: 48-5941-80, Clone: PK136, 1:100.
Ly-6G, BB700, BD Biosciences, Cat: 566435, Clone: 1A8, 1:200.
Ly-6C, BV570, BioLegend, Cat: 128029, Clone: HK1.4, 1:200.
Siglec F, PE, BD Biosciences, Cat: 562068, Clone: E50-2440, 1:100.
CD11b, APC-Cy7, BioLegend, Cat: 101226, Clone: M1/70, 1:200.
CD11c, PE-Cy5, BioLegend, Cat: 117316, Clone: N418, 1:100.
CD115, PE-Cy7, Invitrogen, Cat: 25-1152-82, Clone: AFS98, 1:100.
CD24, BV510, BioLegend, Cat: 101831, Clone: M1/69, 1:100.
CD19, Biotin, BioLegend, Cat: 115504, Clone: 6D5, 1:50.
Nk1.1, Biotin, BioLegend, Cat: 108704, Clone: PK136, 1:50.
Siglec H, Biotin, Miltenyi, Cat: 130-112-293, Clone: REA819, 1:50.
CD9, Percp-Cy5.5, BioLegend, Cat: 124817, Clone: MZ3, 1:100.
OLR1, PE, R&D, Cat: FAB1564P, Clone: 214012, 1:20.
Ly-6G, PE-Cy5, BioLegend, Cat: 127671, Clone: 1A8, 1:100.
TIMD4, PE-Cy7, BioLegend, Cat: 130009, Clone: RMT4-54, 1:200.
VSIG4, AF488, Invitrogen, Cat: 53-5752-82, Clone: NLA14, 1:100.
CD319, APC, BioLegend, Cat: 152003, Clone: 4G2, 1:100.
MHCII, AF700, BioLegend, Cat: 107622, Clone: M5/114.15.2, 1:400.
F4/80, APC-Cy7, BioLegend, Cat: 123117, Clone: BM8, 1:100.
CD11b, AF488, BioLegend, Cat: 101219, Clone: M1/70, 1:400.
Ly-6G, Biotin, BioLegend, Cat: 127604, Clone: 1A8, 1:50.

CD9, PEDazzle 594, BioLegend, Cat: 124822, Clone: MZ3, 1:400.
F4/80, BV785, BioLegend, Cat: 123141, Clone: BM8, 1:100.
IL-1ß, PerCP-eFlour710, Invitrogen, Cat: 46-7114-82, Clone: NJTEN3, 1:100.
CD3, BUV395, BD Biosciences, Cat: 563548, Clone: UCHT1, 1:500.
CD56, BUV737, BD Biosciences, Cat: 612767, Clone: NCAM16.2, 1:500.
CD45, BUV805, BD Biosciences, Cat: 612892, Clone: HI30, 1:500.
CD11c , BV421, BD Biosciences, Cat: 562561, Clone: B-ly6, 1:50.
CD14, BV510, BioLegend, Cat: 367123, Clone: M5E2, 1:100.
HLA-DR, BV570, BioLegend, Cat: 307637, Clone: L243, 1:100.
CD64, BV605, BioLegend, Cat: 305033, Clone: 10,1, 1:50.
CD1c, BV711, BioLegend, Cat: 331535, Clone: L161, 1:100.
CD19, BV750, BD Biosciences, Cat: 747161, Clone: HIB19, 1:100.
CD163, BV786, BioLegend, Cat: 305441, Clone: GHI/61, 1:50.
CD68, FITC, Invitrogen, Cat: 11-0689-42, Clone: eBioY1/82A, 1:50.
CD63, PerCP-Cy5.5, BioLegend, Cat: 143911, Clone: H5C6, 1:100.
OLR1, PE, BioLegend, Cat: 358603, Clone: 15C4, 1:50.
CD11b, PE-Cy5, BioLegend, Cat: 101209, Clone: M1/70, 1:50.
CD9, PE-Vio770, Miltenyi Biotech, Cat: 130-103-990, Clone: SN4 C3-3A2, 1:20.
TREM2, APC, R&D, Cat: FAB17291A, Clone: Q99NH8, 1:20.
CD16, AF647, BioLegend, Cat: 360713, Clone: 3g8, 1:100.
CD9, PE, BioLegend, Cat: 124805, Clone: MZ3, 1:100.
TIM4, AF647, BioLegend, Cat: 130007, Clone: RMT4-54, 1:200.
F4/80, PE/Cy7, BioLegend, Cat: 123113, Clone: BM8, 1:100.
CD11b, APC/Cy7, BioLegend, Cat: 101226, Clone: M1/70, 1:100.
CD45, PEDazzle 594, BioLegend, Cat: 103145, Clone: 30-F11, 1:100.
Ly-6C, PercpCy5.5, BioLegend, Cat: 128011, Clone: HK1.4, 1:400.
Ly-6G, AF488, BioLegend, Cat: 127625, Clone: 1A8, 1:200.
NK1.1, AF488, BioLegend, Cat: 108717, Clone: PK136, 1:100.
CD3, AF488, BioLegend, Cat: 100212, Clone: 17A2, 1:100.
CD19, AF488, BioLegend, Cat: 115524, Clone: 6D5, 1:200.
MHCII, BV785, BioLegend, Cat: 107645, Clone: M5/114.15.2, 1:400.
OLR1, unconjugated, R&D, Cat: AF1798, Clone: Polyclonal, 1:80.
CD68, unconjugated, Abcam, Cat: ab783, Clone: PG-M1, 1:50.
COL1A1, unconjugated, SouthernBiotech, Cat: 1310-01, Clone: Polyclonal, 1:300.
VCAM1, unconjugated, R&D, Cat: MAB8091, Clone: 1027433, 1:200.
IBA1, unconjugated, Abcam, Cat: ab221790, Clone: EPR16589, 1:1000.
GPNMB, unconjugated, R&D, Cat:AF2550, Clone: Polyclonal, 1:500.
Trem2, unconjugated, Abcam, Cat: ab305103, Clone: EPR26210-1, 1:1000.
Iba1, unconjugated, Abcam, Cat: ab283346, Clone: EPR16589, 1:1000.
Gpnmb, unconjugated, Bio-Techne, Cat: AF2330-SP, Clone: Polyclonal, 1:200.
Olr1, unconjugated, Abcam, Cat: ab317689, Clone: EPR27871-67, 1:200.
Collagen I, unconjugated, Cambridge Bioscience, Cat: 1310-01, Clone: Polyclonal, 1:100.
Timd4, unconjugated, Bio-Techne, Cat: AF2826-SP, Clone: Polyclonal, 1:200.
CD31, AF750, Akoya Biosciences, Cat: 4150017, Clone: EP3095, 1:200.
CD4, AF647, Akoya Biosciences, Cat: 4550112, Clone: EPR6855, 1:200.
SMA, AF750, Akoya Biosciences, Cat: 4450049, Clone: 1A4, 1:200.
E-cadherin, AF550, Akoya Biosciences, Cat: 4250021, Clone: 4A2C7, 1:200.
CD68, AF647, Akoya Biosciences, Cat: 4550113, Clone: KP1, 1:200.
CD45, AF550, Akoya Biosciences, Cat: 4250099, Clone: D9M8I, 1:200.
Vimentin, AF750, Akoya Biosciences, Cat: 4450050, Clone: 091D3, 1:200.
CD8, AF550, Akoya Biosciences, Cat: 4250017, Clone: 53-6.7, 1:200.
IDO1, AF647, Akoya Biosciences, Cat: 4550123, Clone: V1NC3IDO, 1:200.
HLA-A, AF550, Akoya Biosciences, Cat: 4250100, Clone: EP1395Y, 1:200.
HLA-DR, AF750, Akoya Biosciences, Cat: 4450095, Clone: AKYP0063, 1:200.
CD14, AF550, Akoya Biosciences, Cat: 4450047, Clone: EPR3653, 1:200.
Collagen IV, AF647, Akoya Biosciences, Cat: 4550122, Clone: N/A, 1:200.
CD20, AF750, Akoya Biosciences, Cat: 4450094, Clone: AKYP0049, 1:200.
Pan-Cytokeratin, AF750, Akoya Biosciences, Cat: 4450093, Clone: AE-1/AE-3, 1:200.
CD163, AF550, Akoya Biosciences, Cat: STP7000, Clone: N/A, 1:200.
CD3e, AF647, Akoya Biosciences, Cat: 4550125, Clone: EP449E, 1:200.
CD79a, AF750, Akoya Biosciences, Cat: 4450078, Clone: N/A, 1:200.
EpCAM, AF750, Akoya Biosciences, Cat: 4450088, Clone: D9S3P, 1:200.
MPO, AF550, Akoya Biosciences, Cat: 4250083, Clone: E1E7I, 1:200.
Beta-actin, AF750, Akoya Biosciences, Cat: 4450092, Clone: W16197A, 1:200.
Collagen I, AF750, Cambridge Bioscience, Cat: 1310-01, Clone: Polyclonal, 1:200.
S100A4, AF750, BioLegend, Cat: 810101, Clone: S100A4, 1:100.
GPNMB, AF647, Bio-Techne, Cat: AF2550-SP, Clone: Polyclonal, 1:200.
MyH11, AF550, Abcam, Cat: ab240983, Clone: SP314, 1:400.
MNDA, AF647, Novus Bio, Cat: NBP3-18135, Clone: Polyclonal, 1:400.
ASGR1, AF750, Bio-Techne, Cat: MAB43941-SP, Clone: 950203, 1:400.
IBA1, AF750, Abcam, Cat: ab221790, Clone: EPR16589, 1:200.
OLR1, AF647, Abcam, Cat: ab234962, Clone: EPR20750, 1:100.
Collagen III, AF750, Cambridge Bioscience, Cat: 1330-01, Clone: 1330-01, 1:100.
LYVE1, AF750, Abcam, Cat: ab232935, Clone: EPR21857, 1:100.
CD11c, AF647, Cell Signaling Technology, Cat: 97585, Clone: D1V9Y, 1:200.
CD9, AF647, Abcam, Cat: ab307085, Clone: EPR27551-92, 1:400.

CD15, AF647, BioLegend, Cat: 301902, Clone: HI98, 1:200.
Donkey anti-Mouse IgG, AF488, ThermoFisher, Cat: A32766, Clone: Polyclonal, 1:200.
Donkey anti-Rabbit IgG, AF594, ThermoFisher, Cat: A32754, Clone: Polyclonal, 1:200.
Rat IgG2A Antibody, PE, R&D, Cat: IC006P, Clone: 54447, 1:20.

Validation

CD11b, BUV737, Reactivity: Mouse (QC Testing). Application: Flow cytometry (Routinely Tested). Validated in mouse bone-marrow cells.
CD11c, BV421, Verified Reactivity Mouse. Application FC - Quality tested. Validated in mouse splenocytes.
CD45, BV510, Verified Reactivity Mouse. Application FC - Quality tested. Validated in mouse splenocytes.
CD45, BV785, Verified Reactivity Mouse. Application FC - Quality tested. Validated in mouse splenocytes.
CD45.1, PEDazzle 594, Verified Reactivity Mouse. Application FC - Quality tested. Validated in mouse splenocytes.
CD45.2, BV785, Verified Reactivity Mouse. Application FC - Quality tested. Validated in mouse splenocytes.
Ly-6C, BV605, Verified Reactivity Mouse. Application FC - Quality tested. Validated in mouse bone-marrow cells.
CD24, BV510, Verified Reactivity Mouse. Application FC - Quality tested. Validated in mouse splenocytes.
Siglec F, Biotin, Reactivity: Mouse (QC Testing). Application: Flow cytometry (Routinely Tested). Validated in mouse bone-marrow cells.
CD3, Biotin, Verified Reactivity Mouse. Application FC - Quality tested. Validated in mouse splenocytes.
CD3, eflour450, Species Reactivity Mouse. Flow Cytometry (Flow) 0.5 µg/test. Validated in mouse lymph node cells.
CD19, eflour450, Species Reactivity Mouse. Flow Cytometry (Flow) 0.5 µg/test. Validated in mouse splenocytes.
Nk1.1, eflour450, Species Reactivity Mouse. Flow Cytometry (Flow) 1 µg/test. Validated in mouse splenocytes.
Ly-6G, BB700, Reactivity: Mouse (QC Testing). Application: Flow cytometry (Routinely Tested). Validated in mouse bone-marrow leucocytes.
Ly-6C, BV570, Verified Reactivity Mouse. Application FC - Quality tested. Validated in mouse bone-marrow cells.
Siglec F, PE, Reactivity: Mouse (QC Testing). Application: Flow cytometry (Routinely Tested). Validated in mouse bone-marrow myeloid cells.
CD11b, APC-Cy7, Verified Reactivity Mouse, Human, Cynomolgus, Rhesus. Application FC - Quality tested. Validated in mouse bone-marrow cells.
CD11c, PE-Cy5, Verified Reactivity Mouse. Application FC - Quality tested. Validated in mouse splenocytes.
CD115, PE-Cy7, Species Reactivity Mouse. Flow Cytometry (Flow) 0.125 µg/test. Validated in mouse bone marrow cells.
CD24, BV510, Verified Reactivity Mouse. Application FC - Quality tested. Validated in mouse splenocytes.
CD19, Biotin, Verified Reactivity Mouse. Application FC - Quality tested. Validated in mouse splenocytes.
Nk1.1, Biotin, Verified Reactivity Mouse. Application FC - Quality tested. Validated in mouse splenocytes.
Siglec H, Biotin, Species reactivity mouse. Applications: FC. Validated in mouse splenocytes.
CD9, Percp-Cy5.5, Verified Reactivity Mouse. Application FC - Quality tested. Validated in mouse bone-marrow myeloid cells.
OLR1, PE, Species Reactivity Mouse. Applications Flow Cytometry Recommended Concentration 10 µl/106 cells. Validated in RAW 264.7 mouse cell line.
Ly-6G, PE-Cy5, Verified Reactivity Mouse. Application FC - Quality tested. Validated in mouse bone-marrow cells.
TIMD4, PE-Cy7, Verified Reactivity Mouse. Application FC - Quality tested. Validated in mouse peritoneal macrophages.
VSIG4, AF488, Species Reactivity Mouse. Flow Cytometry (Flow) 0.5 µg/test. Validated in resident mouse peritoneal macrophages.
CD319, APC, Verified Reactivity Mouse. Application FC - Quality tested. Validated in mouse splenocytes.
MHCII, AF700, Verified Reactivity Mouse. Application FC - Quality tested. Validated in mouse splenocytes.
F4/80, APC-Cy7, Verified Reactivity Mouse. Application FC - Quality tested. Validated in mouse peritoneal macrophages.
CD11b, AF488, Verified Reactivity Mouse, Human, Cynomolgus, Rhesus. Application FC - Quality tested. Validated in mouse bone-marrow cells.
Ly-6G, Biotin, Verified Reactivity Mouse. Application FC - Quality tested. Validated in mouse bone-marrow cells.
CD9, PEDazzle 594, Verified Reactivity Mouse. Application FC - Quality tested. Validated in mouse bone-marrow myeloid cells.
F4/80, BV785, Verified Reactivity Mouse. Application FC - Quality tested. Validated in mouse peritoneal macrophages.
IL-1ß, PerCP-eFlour710, Species Reactivity Mouse. Flow Cytometry (Flow) 0.25 µg/test. Validated in mouse peritoneal macrophages.
CD3, BUV395, Reactivity: Human (QC Testing). Application: Flow cytometry (Routinely Tested). Validated in human peripheral blood lymphocytes.
CD56, BUV737, Reactivity: Human (QC Testing). Application: Flow cytometry (Routinely Tested). Validated in human peripheral blood lymphocytes.
CD45, BUV805, Reactivity: Human (QC Testing). Application: Flow cytometry (Routinely Tested). Validated in human peripheral blood leukocytes.
CD11c , BV421, Reactivity: Human (QC Testing). Application: Flow cytometry (Routinely Tested). Validated in human peripheral blood lymphocytes and monocytes.
CD14, BV510, Verified Reactivity Human. Application FC - Quality tested. Validated in human peripheral blood monocytes.
HLA-DR, BV570, Verified Reactivity Human. Application FC - Quality tested. Validated in human peripheral blood lymphocytes.
CD64, BV605, Verified Reactivity Human. Application FC - Quality tested. Validated in human peripheral blood monocytes.
CD1c, BV711, Verified Reactivity Human. Application FC - Quality tested. Validated in human peripheral blood lymphocytes.
CD19, BV750, Reactivity: Human (QC Testing). Application: Flow cytometry (Routinely Tested). Validated in human peripheral blood lymphocytes.
CD163, BV786, Verified Reactivity Human. Application FC - Quality tested. Validated in human peripheral blood monocytes.
CD68, FITC, Species Reactivity Human. Flow Cytometry (Flow) 0,125 µg/test. Validated in human peripheral blood cells.
CD63, PerCP-Cy5.5, Verified Reactivity Human. Application FC - Quality tested. Validated in human thrombin-activated human peripheral blood platelets.
OLR1, PE, Verified Reactivity Human. Application FC - Quality tested. Validated in human peripheral blood monocytes.
CD11b, PE-Cy5, Verified Reactivity Mouse, Human, Cynomolgus, Rhesus. Application FC - Quality tested. Validated in mouse bone marrow cells.
CD9, PE-Vio770, Verified Reactivity Human. Application FC, MICS, IF, IHC. Validated in human peripheral blood mononuclear cells.
TREM2, APC, Species Reactivity Human, Mouse. Applications Flow Cytometry Recommended Concentration 10 µl/106 cells. Validated in human peripheral blood monocytes and TREM2-transfected HEK293 cells.
CD16, AF647, Verified Reactivity Human. Application FC - Quality tested. Validated in human peripheral blood lymphocytes.
CD9, PE, Verified Reactivity Human. Application FC - Quality tested. Validated in mouse bone-marrow cells.
TIM4, AF647, Verified Reactivity Mouse. Application FC - Quality tested. Validated in mouse peritoneal macrophages.
F4/80, PE/Cy7, Verified Reactivity Mouse. Application FC - Quality tested. Validated in mouse peritoneal macrophages.

CD11b, APC/Cy7, Verified Reactivity Mouse, Human, Cynomolgus, Rhesus. Application FC - Quality tested. Validated in mouse bone-marrow cells.
CD45, PEDazzle 594, Verified Reactivity Mouse. Application FC - Quality tested. Validated in mouse splenocytes.
Ly-6C, PercpCy5.5, Verified Reactivity Mouse. Application FC - Quality tested. Validated in mouse bone-marrow cells.
Ly-6G, AF488, Verified Reactivity Mouse. Application FC - Quality tested. Validated in mouse bone-marrow cells.
NK1.1, AF488, Verified Reactivity Mouse. Application FC - Quality tested. Validated in mouse splenocytes.
CD3, AF488, Verified Reactivity Mouse. Application FC - Quality tested. Validated in mouse splenocytes.
CD19, AF488, Verified Reactivity Mouse. Application FC - Quality tested. Validated in mouse splenocytes.
MHCII, BV785, Verified Reactivity Mouse. Application FC - Quality tested. Validated in mouse splenocytes.
OLR1, unconjugated, Species Reactivity Human. Species Reactivity Mouse. Applications Immunohistochemistry Recommended Concentration 5-15 µg/mL. Validated in paraffin-embedded sections of human placenta.
CD68, unconjugated, Anti-CD68 antibody [PG-M1] (ab783) is a mouse monoclonal antibody and is validated for use in IHC-P in human samples. Dilution info 1/10 - 1/40. Validated in human tonsil tissue sections.
COL1A1, unconjugated, Referenced species reactivities include – Human. Other referenced applications for relevant formats include - Immunohistochemistry. Validated in human fibroblasts.
VCAM1, unconjugated, Species Reactivity Human. Species Reactivity Mouse. Applications Immunohistochemistry Recommended Concentration 5-25 µg/mL. Validated in immersion fixed paraffin-embedded sections of human spleen.
IBA1, unconjugated, used to detect Iba1 in Western Blot, IP, IHC-P, IHC-Fr, ICC/IF. Suitable for Human, Mouse, Rat samples. Validated in paraffin-embedded human cerebrum tissue.
GPNMB, unconjugated, Species Reactivity Human. Applications Immunohistochemistry Recommended Concentration 5-15 µg/mL. Validated in fixed paraffin-embedded sections of human liver.
Trem2, unconjugated, Used to detect Trem2 in WB, IHC-IF, IHC-P. Suitable for Mouse. Validated in paraffin-embedded mouse brain (FFPE).
Iba1, unconjugated, Used to detect Iba1 in FC, IP, ICC-IF, IHC-P. Suitable for Human, Mouse, Rat. Validated in paraffin-embedded mouse cerebrum (FFPE).
Gpnmb, unconjugated, Species Reactivity Mouse. Applications IHC Recommended Concentration 0.1 µg/mL. Validated in fixed paraffin-embedded sections of mouse brain.
Olr1, unconjugated, Used to detect Olr1 in WB, IP, mIHC, IHC-P. Suitable for Mouse, Rat. Validated in paraffin-embedded mouse brain (FFPE).
Collagen I, unconjugated, Referenced species reactivities include – Human. Other referenced applications for relevant formats include - IHC. Validated in human fibroblasts.
Timd4, unconjugated, Species Reactivity Mouse. Applications IHC Recommended Concentration 0.1 µg/mL. Validated in fixed paraffin-embedded sections of mouse liver.
CD31, AF750, Verified Reactivity Human, Application PhenoCycler Fusion Recommended titration: 1:200 (Hu FFPE) based on screening with human tonsil (FFPE).
CD4, AF647, Verified Reactivity Human, Application PhenoCycler Fusion Recommended titration: 1:200 (Hu FFPE) based on screening with human tonsil (FFPE).
SMA, AF750, Verified Reactivity Human, Rat, Application PhenoCycler Fusion Recommended titration: 1:200 (Hu FFPE) based on screening with human tonsil (FFPE).
E-cadherin, AF550, Verified Reactivity Human, Rat, Application PhenoCycler Fusion Recommended titration: 1:200 (Hu FFPE) based on screening with human tonsil (FFPE).
CD68, AF647, Verified Reactivity Human, Non-human Primate, Rat, Cat, Application PhenoCycler Fusion Recommended titration: 1:200 (Hu FFPE) based on screening with human tonsil (FFPE).
CD45, AF550, Verified Reactivity Human, Monkey, Application PhenoCycler Fusion Recommended titration: 1:200 (Hu FFPE) based on screening with human tonsil (FFPE).
Vimentin, AF750, Verified Reactivity Human, Application PhenoCycler Fusion Recommended titration: 1:400 (Hu FFPE) based on screening with human tonsil (FFPE).
CD8, AF550, Verified Reactivity Human, Mouse, Rat, Application PhenoCycler Fusion Recommended titration: 1:200 (Hu FFPE) based on screening with human tonsil (FFPE).
IDO1, AF647, Verified Reactivity Human, Application PhenoCycler Fusion Recommended titration: 1:200 (Hu FFPE) based on screening with human tonsil (FFPE).
HLA-A, AF550, Verified Reactivity Human, Application PhenoCycler Fusion Recommended titration: 1:500 (Hu FFPE) based on screening with human tonsil (FFPE).
HLA-DR, AF750, Verified Reactivity Human, Application PhenoCycler Fusion Recommended titration: 1:200 (Hu FFPE) based on screening with human tonsil (FFPE).
CD14, AF550, Verified Reactivity Human, Application PhenoCycler Fusion Recommended titration: 1:200 (Hu FFPE) based on screening with human tonsil (FFPE).
Collagen IV, AF647, Verified Reactivity Human, Application PhenoCycler Fusion Recommended titration: 1:200 (Hu FFPE) based on screening with human tonsil (FFPE).
CD20, AF750, Verified Reactivity Human, Application PhenoCycler Fusion Recommended titration: 1:200 (Hu FFPE) based on screening with human tonsil (FFPE).
Pan-Cytokeratin, AF750, Verified Reactivity Human, Rat, Application PhenoCycler Fusion Recommended titration: 1:200 (Hu FFPE) based on screening with human tonsil (FFPE).
CD163, AF550, Verified Reactivity Human, Mouse, Rat, Application PhenoCycler Fusion Recommended titration: 1:200-1:300 (Hu FFPE) based on screening with human tonsil (FFPE).
CD3e, AF647, Verified Reactivity Human, Application PhenoCycler Fusion Recommended titration: 1:200 (Hu FFPE) based on screening with human tonsil (FFPE).
CD79a, AF750, Verified Reactivity Human, Application PhenoCycler Fusion Recommended titration: 1:200 (Hu FFPE) based on screening with human tonsil (FFPE).
EpCAM, AF750, Verified Reactivity Human, Application PhenoCycler Fusion Recommended titration: 1:200 (Hu FFPE) based on screening with human tonsil (FFPE).
MPO, AF550, Verified Reactivity Human, Application PhenoCycler Fusion Recommended titration: 1:200 (Hu FFPE) based on screening with human tonsil (FFPE).
Beta-actin, AF750, Verified Reactivity Human, Mouse, Rat, Zebrafish, Drosophila, Application PhenoCycler Fusion Recommended titration: 1:500 (Hu FFPE) based on screening with human tonsil (FFPE).
Collagen I, AF750, Referenced species reactivities include – Human. Other referenced applications for relevant formats include - IHC.

Validated in human fibroblasts.
S100A4, AF750, Verified Reactivity Human, Mouse, Rat. Application IHC-P, ICC - Quality tested by IHC staining. Validated in human tonsil (FFPE).
GPNMB, AF647, Species Reactivity Human. Applications IHC Recommended Concentration 5-15 µg/mL. Validated in fixed paraffin-embedded sections of human liver.
MyH11, AF550, Used to detect MyH11 in FC, IP, IHC-P, IHC-Fr, ICC/IF, mIHC. Suitable for Human, Mouse, Rat samples. Validated in paraffin-embedded human colon, prostate, fallopian tubes, ovary tissue.
MNDA, AF647, Reactivity: Human. Application: IHC 1 µg/mL. Validated in human spleen tissue (FFPE).
ASGR1, AF750, Reactivity: Human. Application: IHC 5-25 µg/mL. Validated in human liver tissue (FFPE).
IBA1, AF750, Used to detect IBA1 in IP, IHC-P, IHC-Fr, ICC/IF, mIHC. Suitable for Human, Mouse, Rat samples. Validated in paraffin-embedded human brain, cerebrum tissue (FFPE).
OLR1, AF647, Used to detect OLR1 in WB. Suitable for Human, Mouse, Rat samples. Validated in paraffin-embedded human brain, cerebrum tissue (FFPE).
Collagen III, AF750, Referenced species reactivities include – Human. Other referenced applications for relevant formats include - Immunohistochemistry, flow cytometry, Western blot. Validated in human cancer gastric tissue (FFPE).
LYVE1, AF750, Used to detect LYVE1 in WB, IP, IHC-P. Suitable for Human. Validated in paraffin-embedded human colon, tonsil, liver tissue (FFPE).
CD11c, AF647, Species Reactivity Mouse. Application IHC, IP, WB. Validated in mammary tumour tissue (FFPE).
CD9, AF647, Used to detect CD9 in WB, IP, IHC-P. Suitable for Human, mouse, rat samples.
CD15, AF647, Verified Reactivity Human. Application FC, IHC-F, IHC-P - Quality tested by IHC staining. Validated in human tonsil (FFPE).
Donkey anti-Mouse IgG, AF488, Species Reactivity Mouse. Applications Tested Dilutions Immunocytochemistry (ICC/IF) 1-10 µg/mL. Validated using SH-SY5Y (positive model) and T-47D (negative model) cells stained with Nestin monoclonal antibody product # 14-9843-80.
Donkey anti-Rabbit IgG, AF594, Species Reactivity Rabbit. Applications Tested Dilutions Immunocytochemistry (ICC/IF) 1-10 µg/mL. Validated using MCF 10A (positive model) and A-431 (negative model) cells stained with Vimentin polyclonal antibody product # PA5-27231.
Rat IgG2A Antibody, PE, Specificity Selected for use as rat IgG2A isotype control for flow cytometry applications. Validated in 4T1 mouse breast cancer cell line.

Eukaryotic cell lines

Policy information about cell lines and Sex and Gender in Research

Cell line source(s)	THP-1 cells were originally purchased from ATCC.<br>LX-2 cells were provided by Professor John Iredale, University of Edinburgh.
Authentication	Functional assays were performed to validate cellular responses in keeping with prior literature.
Mycoplasma contamination	All cell lines tested negative for mycomplasma infection
Commonly misidentified lines<br>(See ICLAC register)	None

Animals and other research organisms

Policy information about studies involving animals; ARRIVE guidelines recommended for reporting animal research, and Sex and Gender in Research

Laboratory animals	Adult male C57BL/6JCrl mice aged 8–10 weeks were purchased from Charles River. LY5.1 male mice (CD45.1/CD45.2) were bred in BVS, University of Edinburgh. Mice were housed under specific pathogen-free conditions in a 12hr dark/light cycle.
Wild animals	None
Reporting on sex	Findings relate to male mice
Field-collected samples	N/A
Ethics oversight	All experimental protocols were approved by the University of Edinburgh Animal Welfare and Ethics Board in accordance with UK Home Office Legislation, following ethical guidelines.

Note that full information on the approval of the study protocol must also be provided in the manuscript.

Plants

Seed stocks

Report on the source of all seed stocks or other plant material used. If applicable, state the seed stock centre and catalogue number. If plant specimens were collected from the field, describe the collection location, date and sampling procedures.

Novel plant genotypes

Describe the methods by which all novel plant genotypes were produced. This includes those generated by transgenic approaches, gene editing, chemical/radiation-based mutagenesis and hybridization. For transgenic lines, describe the transformation method, the number of independent lines analyzed and the generation upon which experiments were performed. For gene-edited lines, describe the editor used, the endogenous sequence targeted for editing, the targeting guide RNA sequence (if applicable) and how the editor was applied.

Authentication

Describe any authentication procedures for each seed stock used or novel genotype generated. Describe any experiments used to assess the effect of a mutation and, where applicable, how potential secondary effects (e.g. second site T-DNA insertions, mosiacism, off-target gene editing) were examined.

Flow Cytometry

Plots

Confirm that:

☒ The axis labels state the marker and fluorochrome used (e.g. CD4-FITC).

☒ The axis scales are clearly visible. Include numbers along axes only for bottom left plot of group (a 'group' is an analysis of identical markers).

☒ All plots are contour plots with outliers or pseudocolor plots.

☒ A numerical value for number of cells or percentage (with statistics) is provided.

Methodology

Sample preparation

For preparing samples for flow cytometry and FACS, mouse livers were perfused with ice cold PBS via the Inferior Vena Cava (IVC) and then excised and placed in RPMI (Gibco, 21875034). The livers were mechanically chopped into small fragments with a razor blade, followed by digestion in RPMI and an enzyme cocktail containing 0.625 mg ml–1 collagenase D (Roche, 11088882001), 0.85 mg ml–1 collagenase V (Sigma-Aldrich, C9263-1G), 1 mg ml–1 dispase (Gibco, Invitrogen, 17105-041) and 30 U ml–1 DNase (Roche, 10104159001) at 37°C for 20 min with agitation. Next, the suspension cells were strained through 100μm filters, centrifuged (300g 5 min) and incubated for 3 minutes in 10% red blood cell (RBC) lysis buffer (BioLegend, 420301). After centrifugation (300g 5 min), cells were resuspended in PEB buffer (PBS containing 0.05% FBS and 0.5 mM EDTA) and passed through a 35 μm filter, prior to blocking and antibody staining. Mouse peripheral blood (100-200μL) was collected from the inferior vena cava into tubes containing a 0.5 mM EDTA solution. Blood was incubated twice in 10% RBC lysis buffer (BioLegend, 420301) for 5 minutes on ice. Cells were then centrifuged (300g 5 min), washed in PEB buffer and filtered through a 35 µm filter, prior to blocking and antibody staining.
For isolation of human intrahepatic leucocytes for flow citometry, liver tissue samples were dissociated and incubated at 37 ° C, 5% CO2 in HBSS+/+ (Life Technologies; Thermo Fisher Scientific) containing 0.002% DNase I (Roche; Sigma-Aldrich; Merck) and 0.02% collagenase IV (Thermo Fisher Scientific).). After enzymatic digestion, tissue samples were subjected to mechanical disruption using the GentleMACS (Miltenyi Biotech) dissociator and debris removed by filtration through a 70 µM filter (Griener). Parenchymal cells were removed by centrifugation on a 30% Percoll gradient (GE Healthcare; VWR) followed by leukocyte isolation by a further round of density centrifugation using a Pancoll gradient (PAN Biotech), prior to blocking and antibody staining.

Instrument

Mouse samples were acquired on a six-laser BD LSRFortessa™ Cell Analyzer (BD Biosciences). Human samples were acquired on a 5-laser Cytek Aurora system running SpectraFlow (v.2.2)

Software

Data were analysed using FlowJo™ v.9.9.4 or v10.8 (BD Biosciences) Software.

Cell population abundance

FACS sorting was performed on BD FACSAriaTM II (Becton Dickinson) and routinely yielded purity of over 95% on sort checks. Flow cytometry data was generated with support from the IRR flow cytometry and cell sorting facility, University of Edinburgh.

Gating strategy

The gating strategies used for identifying liver macrophage subpopulations are shown in Extended Data Fig. 7 and Figure 4G. For flow cytometric analysis of blood from bone marrow chimera mice, viable (DAPI-) single CD45.2+ cells were gated. Monocytes were identified as CD11b+ CD115+ cells, having excluded neutrophils (CD11b+ Ly-6G+), eosinophils (Siglec F+) and lineage+ (NK1.1, Siglec H, CD19, CD3) cells. The degree of blood monocyte donor chimerism for each mouse was calculated as the proportion of monocytes which were CD45.1+, and this value was used to normalise the degree of donor chimerism for each liver macrophage subpopulation from the same mouse.
For human liver macrophages, single viable cells (CD45+, Live dead blue-) were gated on. Lymphoid lineage cell populations were then accounted for (CD19-, CD3-, CD56-) and the myeloid compartment was defined (CD64+). TREM2+ OLR1+ cells were gated within the CD64+ myeloid compartment gate. Samples with more than 1,000 viable CD64+ myeloid cells were included for macrophage quantitation.

☒ Tick this box to confirm that a figure exemplifying the gating strategy is provided in the Supplementary Information.
