# Supplementary material for: A human single-cell atlas identifies OLR1+ scar-associated macrophages as a potential therapeutic target for chronic liver disease
Source: Nat Genet. 2026 Sep 28;58(10):2550–65. doi: 10.1038/s41588-026-02774-w (PMC13649586; doi:10.1038/s41588-026-02774-w)
Supplement: Supplementary file 3 [file 41588_2026_2774_MOESM3_ESM.pdf]

nature portfolio

Peer Review File

A Human Single-cell Atlas Identifies OLR1+ Scar-associated Macrophages as a Potential Therapeutic Target for Chronic Liver Disease

Corresponding Author: Professor Prakash Ramachandran

Version 0:

Decision Letter:

29th Jul 2025

Dear Professor Ramachandran,

Your Article, "A Human Single-cell Atlas Identifies OLR1+ Scar-associated Macrophages as a Therapeutic Target for Chronic Liver Disease" has now been seen by 3 referees. You will see from their comments copied below that while they find your work of considerable potential interest, they have raised quite substantial concerns that must be addressed. In light of these comments, we cannot accept the manuscript for publication, but would be very interested in considering a revised version that addresses these serious concerns.

Reviewer #1 appreciates the dataset but has a range of issues with the heterogeneity of the cohort, which is also not sufficiently characterized, and thinks that both cell annotations and the functional part of your study require careful attention and extra validation.

Reviewer #2 also appreciates the resource potential of your dataset but thinks that proposing OLR1 as a potential therapeutic target would need more extensive validation in alternative mouse models. All in all, they think that the biological validation is insufficient.

Reviewer #3 is unsure if your atlas contains new samples and thinks that your results (functional implications of the SAMs populations; ORL1 as a potential therapeutic target) need stronger functional validation with alternative perturbation experiments and mouse models.

We hope you will find the referees' comments useful as you decide how to proceed. If you wish to submit a substantially revised manuscript, please bear in mind that we will be reluctant to approach the referees again in the absence of major revisions.

To guide the scope of the revisions, the editors discuss the referee reports in detail within the team, including with the chief editor, with a view to identifying key priorities that should be addressed in revision and sometimes overruling referee requests that are deemed beyond the scope of the current study. In this case, we ask you to address reviewers' comments in full, clarifying the novelty of your dataset and providing a stronger functional validation of your results, including the use of alternative perturbation experiments and mouse models (in line with what required by Reviewers). We hope that you will find the prioritised set of referee points to be useful when revising your study. Please do not hesitate to get in touch if you would like to discuss these issues further.

If you choose to revise your manuscript taking into account all reviewer and editor comments, please highlight all changes in the manuscript text file. At this stage we will need you to upload a copy of the manuscript in MS Word .docx or similar editable format.

We are committed to providing a fair and constructive peer-review process. Do not hesitate to contact us if there are specific requests from the reviewers that you believe are technically impossible or unlikely to yield a meaningful outcome.

If revising your manuscript:

*1) Include a “Response to referees” document detailing, point-by-point, how you addressed each referee comment. If no

action was taken to address a point, you must provide a compelling argument. This response will be sent back to the referees along with the revised manuscript.

*2) If you have not done so already please begin to revise your manuscript so that it conforms to our Article format instructions, available here.
Refer also to any guidelines provided in this letter.

*3) Include a revised version of any required Reporting Summary: https://www.nature.com/documents/nr-reporting-summary.pdf
It will be available to referees (and, potentially, statisticians) to aid in their evaluation if the manuscript goes back for peer review.
A revised checklist is essential for re-review of the paper.

Please be aware of our guidelines on digital image standards.

EXTENDED DATA FIGURES

When re-submitting your manuscript, please ensure that any supplementary figures and tables that are crucial to the manuscript's conclusions are converted into Extended Data figures and tables to increase visibility of these data. Extended Data figures and tables are online-only (present in the online PDF and full-text HTML versions of the paper), peer-reviewed display items that provide essential background to the article but are not included in the main article due to space constraints. A maximum of ten Extended Data display items (figures and tables) is permitted.

You may use the link below to submit your revised manuscript and related files:

Link Redacted

Note: This URL links to your confidential home page and associated information about manuscripts you may have submitted, or that you are reviewing for us. If you wish to forward this email to co-authors, please delete the link to your homepage.

If you wish to submit a suitably revised manuscript we would hope to receive it within 6 months. If you cannot send it within this time, please let us know. We will be happy to consider your revision so long as nothing similar has been accepted for publication at Nature Genetics or published elsewhere. Should your manuscript be substantially delayed without notifying us in advance and your article is eventually published, the received date would be that of the revised, not the original, version.

Please do not hesitate to contact me if you have any questions or would like to discuss the required revisions further.

Nature Genetics is committed to improving transparency in authorship. As part of our efforts in this direction, we are now requesting that all authors identified as 'corresponding author' on published papers create and link their Open Researcher and Contributor Identifier (ORCID) with their account on the Manuscript Tracking System (MTS), prior to acceptance. ORCID helps the scientific community achieve unambiguous attribution of all scholarly contributions. You can create and link your ORCID from the home page of the MTS by clicking on 'Modify my Springer Nature account'. For more information please visit please visit www.springernature.com/orcid.

Thank you for the opportunity to review your work.

Sincerely,
Chiara

Chiara Anania, PhD
Associate Editor
Nature Genetics
https://orcid.org/0000-0003-1549-4157

Referee expertise:

Referee #1: hepatology, multiomics

Referee #2: liver disease

Referee #3: hepatology, immunology

Reviewers' Comments:

Reviewer #1 (Remarks to the Author):

The article by Papachristoforou, Kong, Collela et al entitled ‘A Human Single-cell Atlas Identifies OLR1+ Scar-1 associated Macrophages as a Therapeutic Target for Chronic Liver Disease’ integrates different public data sets to generate a comprehensive liver cell atlas from 77 patients, containing 649,295 cells in total. The resulting atlas, made available as an online resource, is a particular strength of this paper and to the research community. The study places particular emphasis on myeloid cells, identifying two subtypes of scar-tissue associated macrophages based on the expression of OLR1. The relevance of OLR1+ macrophages is further investigated in a previously published MASLD cohort with clinical outcome data, and using in vivo and in vitro experimental models. Though an interesting and comprehensive approach, there are some limitations, especially concerning the annotations, included aetiologies and models.

Major concerns.
-A specific concern is the classification of ‘healthy’ liver samples, many of which were derived from resection specimens or discarded donor livers that lack proper histological screening. For example, the 5 ‘healthy’ donors from the MacParland study included2 individuals with histories of heavy alcoholism and drug use, and 2 with obesity (and hence possible MASLD). This introduces a bias and impacts the interpretation of the results, meaning that the current study largely compares end-stage cirrhotic and heterogeneously diseased livers with a few normal samples. For instance, the proportion of monocytes in ‘healthy’ is extremely high. This should be taken into account and the insufficiently characterised ‘healthy’ controls, especially the ones lacking clinicopathological data, should be excluded.

- The heterogeneity of the CLD samples, which include end-stage livers from multiple aetiologies, creates an issue concerning the definition of ‘scar-assoiated’ cells. Since liver fibrosis can differ based on aetiology and stage - sinusoidal, centrolobular or portal fibrosis. with variable patterns of bridging (centrolobular-portal or portal-portal), there is a need for greater granularity in the analysis of fibrosis stages and aetiologies, especially when looking at OLR1+ cells.

-The cluster annotation strategy, particularly the a priori selection of cluster marker genes, raises concerns about potential bias and clarity. The second level clustering divides the myeloid cells into KCs, monocytes, SAMs and STAB1+ macrophages. One would expect more granularity here (intermediate or transMACs?). The annotation of the SAMs, for example, relies heavily on the increased expression of one or two markers within that cluster. This does not make all of the cells in that cluster positive for TREM2+CD9+. The cluster also expresses SPP1 and GPNMB, so it might better align with lipid-associated macrophages (LAMs) rather than SAMs? Supp table S3 shows that TREM2 is also highly significant in STAB1+ macrophages. Cluster annotation should be done more unbiased and previously reported subsets should be taken into account.

-Similarly, the criteria for defining scar-associated endothelium are not well defined: it is not clear whether these cells are functionally unique to fibrosis or simply more prevalent in end-stage disease? The naming based solely on association rather than proven causality is confusing. Features like neo-angiogenesis and hepatocellular senescence also occur together in end-stage liver disease but one would not call them senescence-associated endothelium. The general criteria for defining clusters require more attention.

-A 4-week CCl4 model is rather acute and the fibrosis deposition will be limited. A previous publication (PMID: 35750138) reported Trem2+ cells in an acute MASH model (ldlr-/- mice on 2 weeks of HFC diet), highlighting associated markers as Cd9, Gpnmb, Lgals3 or Cd63. Are the ‘SAMs’ acute responders rather than fibrosis-associated? This could have a profound impact on the nomenclature/annotation. In addition, how relevant is the CCL4 model (a toxic model) to the different human aetiologies presented in this paper? Either different models should be included to mirror the different aetiologies or be taken out altogether.

-Line 254. ‘Having identified 2 distinct populations of TREM2+ CD9+ SAMac’. The paper presents expression mainly using dot plots. This is rather confusing as it only shows some enrichment of certain markers in that cluster. This does not mean that all the cells within that cluster express these markers. STAB1 also seems to be expressed in SAMs The paper would benefit from including some feature plots, especially for the main markers (OLR1, TREM2, CD9, etc). What is the percentage of these cells within the myeloid population?

-Table S11. Differentially expression of SAM1 with SAM2. Looking at the DEGs, one would almost say that this is a comparison between LAMs and SAMs (FABP5, GPNMB, CD36, LPL). Assuming that this is a differentially expression analysis between clusters, it would make more sense to compare TREM2+CD9+ cells from cluster 1 with TREM2+CD9+ cells from cluster 2.

-Line 266 “OLR1+ CD68+ SAMac2 (Fig. 3D; red arrows) were distinct from GPNMB+ CD68+ SAMac1 (Fig. 3D; yellow arrows)”. The used antibodies are rather limited and will capture different cell populations as well, making it difficult to make any conclusions. Adding markers to the staining panel, such as TREM2 and CD9, well help to identify SAMs with more certainty. Including different aetiologies and stages reflecting the transcriptomics data would strengthen the paper.

Minor
-Statistical significance should be added in the main figures where possible, especially with the MiloR plots.

-In chronic liver disease, zonation is gradually lost. How can the authors explain zonated KCs in cirrhosis? Furthermore, KC have been shown to be mobile within the parenchyma (Peiseler M et al 2023).

-Shiny app. Cholangiocyte markers are expressed in a subset of macrophages and mesenchymal cells. Is this a limitation of

the annotation or a technical issue?

-OLR1 is widely expressed in different macrophage clusters (Shiny app). In line, OLR1 is widely expressed in different myeloid clusters and high in monocytes (livercellatlas). How does this impact clinical outcome? Are monocytes predictive rather than OLR1+ SAMs?

-The MASLD data set from Gibben C et al 2024 indicates a poor overlap between TREM2 and OLR1. Is there an aetiology impact?

-Table S3: level 2 annotations. Numbers for the repeats are added to column 1 (e.g. TREM21 and TREM2 gene IDs are used). Best to remove column 1.

-Receptor ligand interaction overstated. Supp Table 15 suggests a wide interactions with different cell types. Integrins are widespread receptors for ECM and can interact with a multitude of cell types. Moreover, some of the highlighted markers in the myeloid cells will be rather lowly lowly expressed on RNA level, making the biological relevance of these interactions questionable. For instance, CCL20 is mainly expressed by epithelial cells, complement is mainly produced by hepatocytes.

-Format supp tables. Some genes are annotated as dates.

Reviewer #2 (Remarks to the Author):

This study by Papachristoforou, Kong, Colella et al. aims at providing an extended cellular atlas of chronically diseased livers based on multiple, previously published, single cell transcriptomic datasets. The authors then identified OLR1 as a marker of a specific scar-associated macrophage population particularly enriched in CLD livers, and explored the functional implications of OLR1 on the macrophage – fibroblast interactions.
The manuscript opens by describing the integration and re-analysis of previously published single cell transcriptomic datasets. The analyses appear very well done to the Reviewer, with the highest standards in terms of data quality and data sharing. For this, the Reviewer wants to congratulate the authors. This aspect truly makes the manuscript timely and this will serve as an extremely valuable resource for the field. Nonetheless, the aspects on OLR1 validation as a potential therapeutic target appear less convincing and additional attention is required on the related experiments. Overall, the Reviewer is convinced by the data that there are SAMac1 and SAMac2 cell subsets that are relevant for further studies and characterization in CLD, yet the importance of OLR1 as a stand-alone therapeutic target remains to be corroborated. Could instead OLR1 be one of several markers identifying SAMac2, and that potentially other genes or proteins should be co-targeted for an effective response in vivo and in vitro?

The reference list seems appropriate. The text is very well written and the abstract, introduction and conclusions reflect the content of the manuscript.

Specific comments
- Considering this relative low number of patients and heterogeneities in disease etiologies, individual patient characteristics may have profound impact on the pool of “rare” cells herein described. This comes with additional complexity due to study variations. While this is well described into the manuscript, how can the authors take this more into considerations? Are the identified SAMac2 more prominent in some datasets compared to others?
- Maybe this Reviewer missed the information, but could the authors provide some information on how efficient their (and the ones from earlier studies) method for single cell RNAseq is? Meaning, how many cells are effectively analysed per amount of tissue? And could the authors provide this value for each individual sample for QC and comparison?
- Fig.1D is particularly valuable in understanding the specificities of each dataset used. Could the authors elaborate on how the discrepancies in cell types could be explained? How much does that influence the following analyses?
- The transcriptomic data pointing towards OLR1 seems very clear. However, the OLR1 staining data presented in Fig.3C/3D and Extended Fig.7A is moderately convincing and additional validation at the protein level would appear important, for OLR1 to be considered a valid therapeutic target. Can the authors provide additional images with a better resolution, and rule out unspecific signal? How many positive cells have been observed in the tissues? Is this observation consistent between different etiologies of CLD? The Reviewer has similar questions on the flow cytometry shown in Extended Fig. 9C. The population of OLR1+ macrophages seems very small and not well defined in the flow cytometry results, meaning there seems to be a gradient of OLR1 expression across SAMac1 and SAMac2. This may question the specificity of an OLR1-targeted therapy if, at least at the protein level, there does not seem to be strong differences in levels of OLR1 expression (if we assume the protocols used for IF and FC are robust).
- There is a typo in the title of Fig. 5I.
- May the authors describe the overall morphology of the spheroid in Fig.6B? And provide a scale bar, as well as potentially additional images and characterization of the in vitro culture system.
- There is a typo page 15 “of the of the CSF1R inhibitor Pexidartinib”.
- The effects of the siRNA OLR1 appear moderate, so does the use of the conditioned media on LX2 gene expression pattern. Besides, additional experiments would be needed to confirm LX-2 are less fibrogenic in this set-up. One may consider evaluating the effect of siRNA OLR1 on monocytes or macrophages prior to seeding them into the multilineage spheroids?
- The Reviewer does not understand why a CSF1R was used to block OLR1+ macrophages. Is that a specific approach? Could the authors instead use a blocking antibody against OLR1?

Reviewer #2 (Remarks on code availability):

The Reviewer does not have the expertise to review the code.

Reviewer #3 (Remarks to the Author):

This is an important article from leading experts in the field with some intriguing results.

General comment #1: The main value of the paper is the Human Single-cell Atlas of healthy vs diseased livers. This work builds on the prior Nature paper (from 2019) by these authors. However, there is a note of caution. In their initial Nature paper, the authors labeled a population of “scar-associated macrophages” (SAMs) as “pro-fibrogenic). A large series of subsequent articles, mainly with mouse models, demonstrated clearly that most of these SAMs are anti-fibrotic and help to counteracting inflammation/fibrosis. In the current work, the authors show that SAMs have different populations, of which some are profibrogenic whereas others have a more anti-fibrotic phenotype. The functional experiments supporting this assumption (which is grounded on the transcriptomic and spatial analyses) are comparatively weak, and I am concerned that the authors over-interpret their findings again (similar as in the 2019 Nature paper).

General comment #2: The authors apparently built their Human Single-cell Atlas entirely on previously published data-sets. This comes with a lot of challenges (different cell isolation protocols, different sequencing depth, heterogeneous patient sources, limited clinical data). In addition, it dampens my enthusiasm regarding the novelty of the data. By the way, the fact that no original scRNA-seq data from human liver were generated for this study is a bit hidden and only apparent from a supplementary table. Also, for linking marker expression (e.g. OLR1) to outcome, the authors use an already published data-set (Steato-SITE), which limits originality of the work.

Additional specific comments:

3.The authors identify OLR1+ CD68+ SAMac2 (inflammatory profile) vs. GPNMB+ CD68+ SAMac1 (anti-inflammatory) in diseased livers as populations of CD9+ TREM2+ SAMs. I am not convinced that the functional implications of these cells are clarified by the authors. For instance, Fig. 6 does only include experiments on reducing OLR1, but not on the other population. For a better functional understanding, one would need either selective depletion models in mice (ideally in clinically relevant MASH-fibrosis models, depleting either one of the two SAM subsets) and convincing in vitro settings, in which liver-educated primary SAM populations are co-cultured with primary hepatocytes and primary HSCs. The work with cell-lines (LX2, H9 spheroids) and the lack of clear experimental conditions comparing SAMac1 vs SAMac2 in a multicellular system is not sufficient.

4. The authors do not show clear data whether the both CD9+ TREM2+ SAMs, OLR1+ CD68+ SAMac2 (inflammatory profile) vs. GPNMB+ CD68+ SAMac1 (anti-inflammatory), have a distinct spatial pattern in the liver (mouse and human). The few “representative pictures” cannot clarify this, a statistical analysis of the spatial organization is missing. In this regard, it would be important to show that the balance of the two populations is affected in progression vs regression conditions.

5. One prerequisite for studying functional consequences of macrophage populations in mice is that they are conserved across species. The authors show this for the CCl4 mode, but this certainly limited translational value. Do these populations also occur (and if so, when and where) in MASH-fibrosis mouse models? Interventions in such models would be more informative.

6. The siRNA-silencing experiments with THP-1 cells are not sufficient to convince me that targeting OLR1 in liver macrophages would be a useful strategy. An inducible genetic depletion in fibrosis models in mice or the selective targeting of primary human SAMs from liver are missing.

Version 1:

Decision Letter:

5th May 2026

Dear Professor Ramachandran,

Your Article, "A Human Single-cell Atlas Identifies OLR1+ Scar-associated Macrophages as a Therapeutic Target for Chronic Liver Disease" has now been seen by 3 referees. You will see from their comments below that while they find your work of interest, some remaining points are raised. We are interested in the possibility of publishing your study in Nature Genetics, but would like to consider your response to these concerns in the form of a revised manuscript before we make a final decision on publication.

To guide the scope of the revisions, the editors discuss the referee reports in detail within the team, including with the chief editor, with a view to identifying key priorities that should be addressed in revision and sometimes overruling referee requests that are deemed beyond the scope of the current study. In this case, please address the remaining points in full. We

hope that you will find the prioritized set of referee points to be useful when revising your study. Please do not hesitate to get in touch if you would like to discuss these issues further.

We therefore invite you to revise your manuscript taking into account all reviewer and editor comments. Please highlight all changes in the manuscript text file. At this stage we will need you to upload a copy of the manuscript in MS Word .docx or similar editable format.

We are committed to providing a fair and constructive peer-review process. Do not hesitate to contact us if there are specific requests from the reviewers that you believe are technically impossible or unlikely to yield a meaningful outcome.

When revising your manuscript:

*1) Include a "Response to referees" document detailing, point-by-point, how you addressed each referee comment. If no action was taken to address a point, you must provide a compelling argument. This response will be sent back to the referees along with the revised manuscript.

*2) If you have not done so already please begin to revise your manuscript so that it conforms to our Article format instructions, available
here.
Refer also to any guidelines provided in this letter.

*3) Include a revised version of any required Reporting Summary: https://www.nature.com/documents/nr-reporting-summary.pdf
It will be available to referees (and, potentially, statisticians) to aid in their evaluation if the manuscript goes back for peer review.
A revised checklist is essential for re-review of the paper.

Please be aware of our guidelines on digital image standards.

EXTENDED DATA FIGURES

When re-submitting your manuscript, please ensure that any supplementary figures and tables that are crucial to the manuscript's conclusions are converted into Extended Data figures and tables to increase visibility of these data. Extended Data figures and tables are online-only (present in the online PDF and full-text HTML versions of the paper), peer-reviewed display items that provide essential background to the article but are not included in the main article due to space constraints. A maximum of ten Extended Data display items (figures and tables) is permitted.

Please use the link below to submit your revised manuscript and related files:

Link Redacted

Note: This URL links to your confidential home page and associated information about manuscripts you may have submitted, or that you are reviewing for us. If you wish to forward this email to co-authors, please delete the link to your homepage.

We hope to receive your revised manuscript within four to eight weeks. If you cannot send it within this time, please let us know.

Please do not hesitate to contact me if you have any questions or would like to discuss these revisions further.

Nature Genetics is committed to improving transparency in authorship. As part of our efforts in this direction, we are now requesting that all authors identified as 'corresponding author' on published papers create and link their Open Researcher and Contributor Identifier (ORCID) with their account on the Manuscript Tracking System (MTS), prior to acceptance. ORCID helps the scientific community achieve unambiguous attribution of all scholarly contributions. You can create and link your ORCID from the home page of the MTS by clicking on 'Modify my Springer Nature account'. For more information please visit please visit www.springernature.com/orcid.

We look forward to seeing the revised manuscript and thank you for the opportunity to review your work.

Sincerely,
Chiara

Chiara Anania, PhD
Associate Editor
Nature Genetics
https://orcid.org/0000-0003-1549-4157

Reviewers' Comments:

Reviewer #1 (Remarks to the Author):

Congratulations on the substantial improvements made to the manuscript. The revisions have clearly strengthened the study, and the overall quality has increased. I only have a few remaining comments, mainly related to the spatial proteomics and to explicitly acknowledging limitations in this study.

1. First, the newly generated human scRNA-seq MASLD cohort does not appear to be accessible via GSE298719 as indicated. If this dataset has been previously published, please provide the appropriate reference.
2. Regarding my previous comment on cell cluster annotation, I agree with the authors' decision to avoid labeling these cells as LAMs. However, the current annotation of SAMac appears to rely on the increased expression of a limited number of genes. Importantly, this population may reflect disease activity rather than fibrosis-specific macrophages, as also suggested by the acute CCL4 mouse model. In the referenced dataset (GSE135251), OLR1 and TREM2 expression are more strongly associated with disease activity (NAS score) rather than fibrosis stage. Given this, the limitations of the current nomenclature and interpretation should be more explicitly discussed. Why SAMacs and not disease activity-associated?
3. For the spatial proteomics data, the strategy used to annotate cell populations is not clearly described. The detection of GPNMB in neutrophils is unexpected and not supported by the scRNA-seq data, suggesting possible annotation inaccuracies or autofluorescence artifacts. What background substration approaches have been implemented?
Furthermore, it remains unclear how SAMacs were defined at the protein level. Based on the supplementary panel, one would assume a TREM2+CD9+ definition. However, TREM2 is not included in the panel shown in Fig. 3c. If this omission is due to technical constraints, then the annotated population likely represents OLR1+ macrophages rather than bona fide OLR1+ SAMacs. This distinction is important, as it limits the strength of the conclusions and may lead to overinterpretation. It would be more accurate to describe cells strictly based on the markers that were directly measured, rather than using an unclear annotated population.
The spatial proteomics has been done on 10 healthy and 36 CLD sections. The underlying aetiology and, more importantly, the stage of the disease has not clearly been stated or shown. As it seems now, the quantification heavily relies on transplanted material or resected material, and not on needle biopsies.
Additionally, Fig. 3H does not show enrichment of GPNMB+ SAMacs in hepatocytic regions, which appears inconsistent with Fig. 3M and should be clarified.
It is also unclear how ductular versus fibrotic regions were distinguished, as these compartments often overlap. The inclusion of periportal hepatocytes within ductular areas may partly explain the observed differences and should be addressed.
In Fig. 3I, the presence of double-positive cells at the protein level is evident and should be acknowledged in the text. From a visualisation perspective, using red instead of orange for GPNMB would improve clarity.
4. Fig ED 7F. Are the statistics missing here or is there no sig difference?
5. Finally, in the multi-organoid culture system, the use of relatively high concentrations of dexamethasone should be discussed as a limitation. While beneficial for hepatocyte survival, dexamethasone is known to influence macrophage polarisation and may affect the interpretation of the results.

Reviewer #2 (Remarks to the Author):

The Authors have comprehensively and appropriately addressed the Reviewer's comments.

Reviewer #2 (Remarks on code availability):

This Reviewer does not have the expertise to review the code.

Reviewer #3 (Remarks to the Author):

I am generally happy with the revised manuscript, it reports an interesting observation and will stimulate further research in the field.

However, some of my major comments were not really addressed (e.g. deciphering the true function of the SAMac subsets). I am also puzzled by the fact that the authors added scRNA-seq data from the BDL mouse model, but not from MASH-fibrosis models. This makes it more difficult to link the data to the human liver atlas, in which different stages of MASLD/MASH are represented, but neither cholestatic nor toxic injury (as modelled by CCl4 or BDL).

I understand that the in-vivo targeting of SAMac populations or their key distinguishing molecular marker was beyond the scope of the paper (or the capacity of the team). Hence, I support publication.

Typo: Resmiterom (should be resmetirom).

Version 2:

Decision Letter:

Our ref: NG-A69391R1

14th Jul 2026

Dear Dr. Ramachandran,

Thank you for submitting your revised manuscript "A Human Single-cell Atlas Identifies OLR1+ Scar-associated Macrophages as a Therapeutic Target for Chronic Liver Disease" (NG-A69391R1). It has now been seen by the original referees and their comments are below. The reviewers find that the paper has improved in revision, and therefore we'll be happy in principle to publish it in Nature Genetics, pending minor revisions to comply with our editorial and formatting guidelines.

If the current version of your manuscript is in a PDF format, please email us a copy of the file in an editable format (Microsoft Word or LaTex)-- we can not proceed with PDFs at this stage.

We are now performing detailed checks on your paper and will send you a checklist detailing our editorial and formatting requirements soon. Please do not upload the final materials and make any revisions until you receive this additional information from us.

Thank you again for your interest in Nature Genetics Please do not hesitate to contact me if you have any questions.

Congratulations!

Sincerely,
Chiara

Chiara Anania, PhD
Senior Editor
Nature Genetics
https://orcid.org/0000-0003-1549-4157

Reviewer #1 (Remarks to the Author):

The authors have fully answered my comments. Congratulations with the nice paper.

INTERNAL USE ONLY AIP modules required: ["Clinical","Life Science"]

Open Access This Peer Review File is licensed under a Creative Commons Attribution 4.0 International License, which permits use, sharing, adaptation, distribution and reproduction in any medium or format, as long as you give appropriate credit to the original author(s) and the source, provide a link to the Creative Commons license, and indicate if changes were made.
In cases where reviewers are anonymous, credit should be given to 'Anonymous Referee' and the source.
The images or other third party material in this Peer Review File are included in the article's Creative Commons license, unless indicated otherwise in a credit line to the material. If material is not included in the article's Creative Commons license and your intended use is not permitted by statutory regulation or exceeds the permitted use, you will need to obtain permission directly from the copyright holder.
To view a copy of this license, visit https://creativecommons.org/licenses/by/4.0/

Response to Reviewers

We are grateful to the reviewers and editors for their careful evaluation and constructive feedback, which have helped strengthen the manuscript. Below, we respond point by point to each comment, detailing the additional experimental data generated, clarifications provided, and revisions made to the text and figures. Key new data in the paper are also highlighted in red font in the main manuscript. We believe that the revised manuscript has been substantially improved as a result of this process, and we appreciate your consideration of our resubmission.

Reviewer #1 (Remarks to the Author):

The article by Papachristoforou, Kong, Collela et al entitled 'A Human Single-cell Atlas Identifies OLR1+ Scar-1 associated Macrophages as a Therapeutic Target for Chronic Liver Disease' integrates different public data sets to generate a comprehensive liver cell atlas from 77 patients, containing 649,295 cells in total. The resulting atlas, made available as an online resource, is a particular strength of this paper and to the research community. The study places particular emphasis on myeloid cells, identifying two subtypes of scar-tissue associated macrophages based on the expression of OLR1. The relevance of OLR1+ macrophages is further investigated in a previously published MASLD cohort with clinical outcome data, and using in vivo and in vitro experimental models. Though an interesting and comprehensive approach, there are some limitations, especially concerning the annotations, included aetiologies and models.

Major concerns.
-A specific concern is the classification of 'healthy' liver samples, many of which were derived from resection specimens or discarded donor livers that lack proper histological screening. For example, the 5 'healthy' donors from the MacParland study included 2 individuals with histories of heavy alcoholism and drug use, and 2 with obesity (and hence possible MASLD). This introduces a bias and impacts the interpretation of the results, meaning that the current study largely compares end-stage cirrhotic and heterogeneously diseased livers with a few normal samples. For instance, the proportion of monocytes in 'healthy' is extremely high. This should be taken into account and the insufficiently characterised 'healthy' controls, especially the ones lacking clinicopathological data, should be excluded.

We entirely agree with the reviewer that the "healthy" liver samples included in scRNAseq studies worldwide are often imperfect. As the reviewer correctly points out, given the prevalence of liver disease in the general population, there are likely to be underlying or subclinical risk factors for CLD in the patients included in these studies. Indeed, there may be additional confounders such as perioperative tissue ischaemia and the presence of disease elsewhere in the liver (e.g. colorectal metastasis), which may have systemic or microenvironmental effects on otherwise non-lesional tissue and specific cell populations. This represents a recognised limitation across the field and highlights the need to validate any findings or candidate therapeutic targets using orthogonal approaches and independent, histologically-staged cohorts. In response to the reviewer's suggestion, we have now explicitly acknowledged this important point in the Discussion section (Page 16).

To directly address the reviewer's concern about potential biases caused by heterogeneity in the healthy liver scRNAseq group, we have conducted additional analyses and generated further data to confirm our key finding of an expansion of OLR1+ macrophages in diseased vs histologically normal liver.

Firstly, as suggested by the reviewer, we have subsetted the scRNAseq data from patients who have histologically normal liver (n=25) reported in the original manuscripts and compared these to CLD livers. As shown in Reviewer Fig 1, our key finding of the expansion of OLR1+ SAMacs still holds true. We have not currently included these data in the manuscript as we feel it adds unnecessary complexity to the interpretation and would rather emphasise the need for validation of findings using independent approaches and cohorts – we believe this is the most important message for the field. Of course, if the reviewer and editors feel that these data should be added, we would be happy to do so.

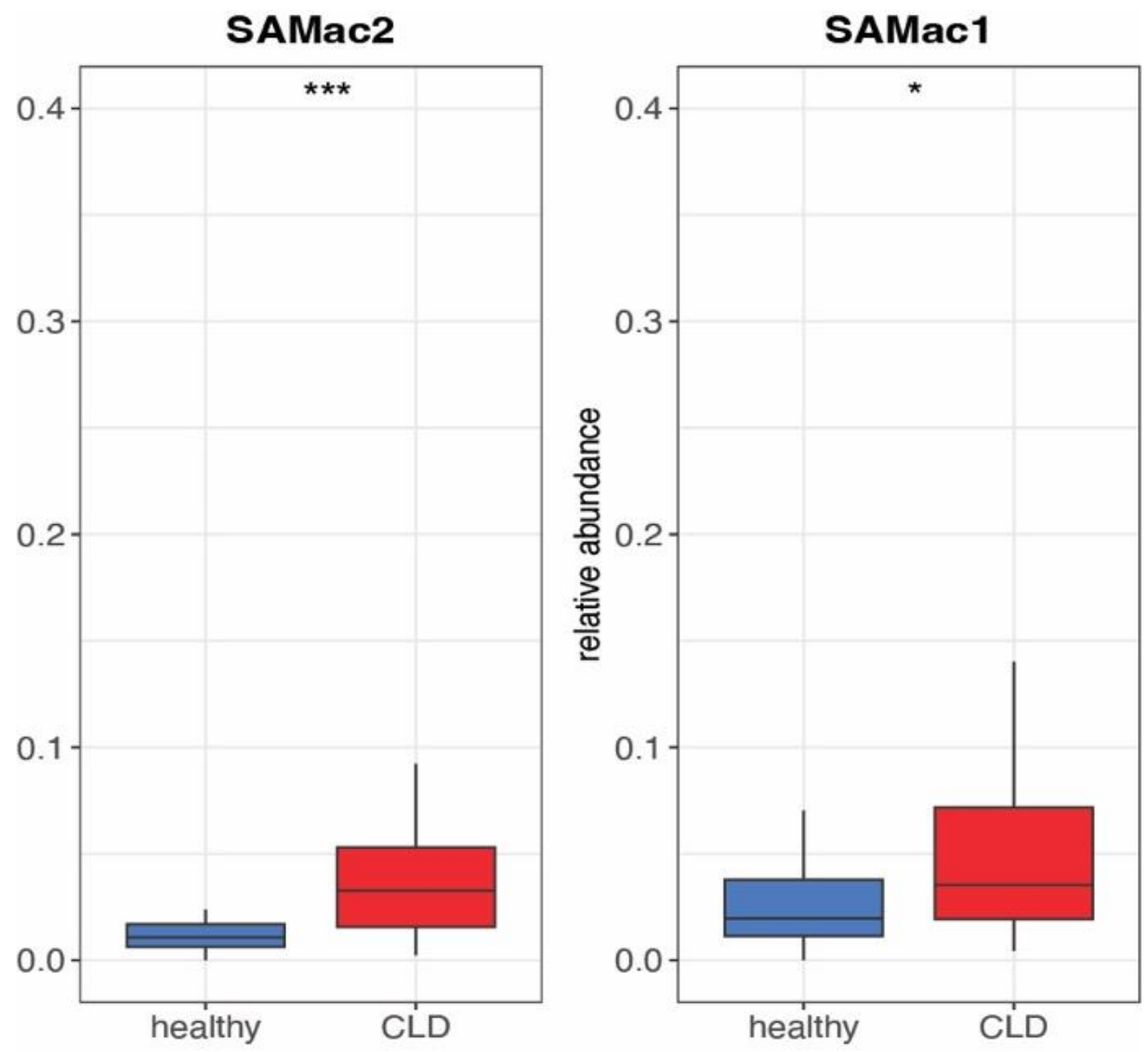

*Reviewer Figure 1: Differential abundance analysis of SAMac2 and SAMac1 clusters between histologically normal liver and CLD livers subsetted from scRNAseq atlas. Statistical comparisons performed by Welsh's t-tests; *p<0.05, ***p<0.001.*

Secondly, we have generated an independent scRNAseq dataset from liver needle biopsy samples, comparing tissue from patients with histologically normal liver to those with fully staged MASLD. Crucially, these needle biopsies substantially reduce confounding factors, such as perioperative tissue ischaemia or the presence of disease elsewhere in the liver. Moreover, comprehensive clinical metadata are available for all cases and controls (Supplementary Table 12). Using our atlas as a reference, we confirm an expansion of SAMacs and an upregulation of SAMac OLR1 expression in MASLD biopsies vs histologically normal controls (Fig. 4A-D).

Thirdly, we have assessed OLR1 expression and SAMac2 accumulation in other human liver biopsy cohorts with linked histological data. Notably, hepatic OLR1 expression increases with histological fibrosis stage in a range of CLDs, whilst deconvolution analysis shows progressive expansion of SAMac2 according to histological fibrosis stage in these different conditions (Figure 4G, I-J, Extended Data Figure 7I-P).

Finally, we have conducted spatial proteomic analysis in histologically-staged patient samples, again confirming expansion of OLR1+ SAMacs in CLD patients (Fig. 3C-G).

Hence, we have shown and reinforced the close association between OLR1+ SAMac expansion and histological disease stage using a range of modalities in a number of independent cohorts, confirming that our findings are robust, irrespective of the potential confounders in the healthy samples included in the atlas. We do not feel that the removal of samples from the atlas dataset will be helpful, because the improved reference data and annotation that we provide for the field is critically dependent on the large number of patients and cells that we have included. Rather, we feel that it is important that researchers using the data should be aware of the limitations of the "healthy" samples and the need for rigorous validation of pertinent findings in separate cohorts with linked clinicopathological data. Our data on OLR1 provides a framework for how best to achieve this.

- The heterogeneity of the CLD samples, which include end-stage livers from multiple aetiologies, creates an issue concerning the definition of 'scar-assoiated' cells. Since liver fibrosis can differ based on aetiology and stage - sinusoidal, centrolobular or portal fibrosis. with variable patterns of bridging (centrolobular-portal or portal-portal), there is a need for greater granularity in the analysis of fibrosis stages and aetiologies, especially when looking at OLR1+ cells.

We agree with the reviewer that there may be differences in scar-associated cells dependent on the stage and aetiology of CLD and the patterns of fibrosis. There are undoubtedly limitations of existing scRNAseq datasets when it comes to addressing this important point, as they are mainly focused on end-stage liver disease. To comprehensively address this across all cell types, it would require more detailed single-cell and spatial profiling of liver biopsy tissue from patients with different disease aetiologies and stages – this remains a work in progress, but is of course something the field should strive for in the forthcoming years.

However, focussing specifically on the OLR1+ cells, we have provided additional data which demonstrates that OLR1+ SAMacs expand across different aetiologies and stages of CLD. We have assessed OLR1 expression in histologically-staged human liver biopsy tissue from a range of CLD aetiologies. Hepatic OLR1 increases with fibrosis stage in MASLD, ARLD, PSC and PBC (Fig. 4G, Extended Data Fig. 7I, K, M, O). Furthermore, deconvolution analysis confirms expansion of SAMac2 across these aetiologies (Fig. 4H, Extended Data Fig. 7J, L, N, P). We have also generated spatial proteomic data using a custom PhenoCycler-Fusion antibody panel. These data confirm that OLR1+ SAMacs expand across different aetiologies of CLD (Extended Data Fig. 7F), and are localised within the fibrotic niche (Fig. 3H-M). Hence, we believe that OLR1+ SAMacs are potentially a target cell population across different aetiologies and stages of CLD.

Importantly, the new data we have added (PhenoCycler-based spatial profiling of immune niches (Fig. 3C-M) and scRNAseq of patient liver needle biopsy material (Fig. 4A-F)) provide methodological advances which could be applied to larger pan-aetiology CLD biopsy cohorts in the future, generating greater understanding of the aetiology and stage-specific changes in the fibrotic niche.

-The cluster annotation strategy, particularly the a priori selection of cluster marker genes, raises concerns about potential bias and clarity. The second level clustering divides the myeloid cells into KCs, monocytes, SAMs and STAB1+ macrophages. One would expect more granularity here (intermediate or transMACs?). The annotation of the SAMs, for example, relies heavily on the increased expression of one or two markers within that cluster. This does not make all of the cells in that cluster positive for TREM2+CD9+. The cluster also expresses SPP1 and GPNMB, so it might better align with lipid-associated macrophages (LAMs) rather than SAMs? Supp table S3 shows that TREM2 is also highly significant in STAB1+ macrophages. Cluster annotation should be done more unbiased and previously reported subsets should be taken into account.

We apologise for the confusion about the cluster annotation strategy. For clarity, clustering was performed in an *unsupervised* manner. Annotation was then performed manually, based on statistically significant marker genes for each cluster. No *a priori* selection of cluster marker genes was carried out for cluster definition and annotation – selected marker genes have been chosen purely for visualisation purposes, but the full marker genes for each cluster are available in the Supplementary Tables. With regards to the level annotation, we first defined level 3 clusters in an unsupervised manner. Level 2 and level 1 were generated after level 3, by merging transcriptionally similar clusters from level 3. We feel that the level 1 and level 2 annotation will be useful for the field, depending on the downstream application, but the level 3 unbiased clustering is the most granular for detailed study of specific cell types, such as SAMacs.

With regard to the MP clusters, the SAMac subtypes were identified not only on one or two marker genes but based on unsupervised clustering. As shown in Supplementary Table 5, there are a total of 1,271 significant marker genes for SAMac1 and 1,499 for SAMac2. Of course, the reviewer is correct that not all of the cells within these clusters are positive for TREM2 and CD9; however, this is always an issue with single-cell RNA-seq data due to dropouts of individual genes, highlighting the need for cell type identification based on unsupervised clustering and expert annotation rather than relying on individual genes. This is the approach we have undertaken in this study.

The reviewer makes a good point about relating MP subpopulations to previously reported subsets. We have attempted to do this, without being bound by nomenclature which predominantly derives from mouse studies. For intermediate/transMACs, there is a cluster of these cells annotated as "Transitioning Mac" in the level 3 MP data. This is a challenging population to annotate, as it is an intermediate cell state and often lacks many unique marker genes. The STAB1+ Macs do express some TREM2 and CD9, but this is much lower than the 2 SAMac populations (Fig. 2B and Supplementary Table 5), hence we annotated them as a distinct type of macrophage. With regard to the LAM vs SAMac nomenclature, the reviewer is right that there is overlap between the transcriptome of LAM and SAMac – indeed, we feel that SAMac1 (highest in GPNMB, FABP5 and CD36) represents the human equivalent of the LAM population defined in mouse and have now referenced this point in the manuscript (Page 8). However, we prefer to call them SAMac rather than LAMs because this population is not purely associated with steatotic disease and lipids, but rather accumulates in areas of scar across different aetiologies of CLD, and indeed is also observed in fibrotic diseases in other organs. We are, of course, happy to consider revising the nomenclature if the reviewer and editors feel strongly about this.

-Similarly, the criteria for defining scar-associated endothelium are not well defined: it is not clear whether these cells are functionally unique to fibrosis or simply more prevalent in end-stage disease? The naming based solely on association rather than proven causality is confusing. Features like neo-angiogenesis and hepatocellular senescence

also occur together in end-stage liver disease but one would not call them senescence-associated endothelium. The general criteria for defining clusters require more attention.

We agree with this point about annotation based on association rather than causality. We annotated the scar-associated endothelial cells as SAEndo1 and SAEndo2 for consistency with our previous publication (Ramachandran P et al, Nature 2019; PMID: 31597160), where we located them in areas of scar tissue. However, the reviewer is correct that these cells could have other functions, and the functional assessment of these populations is beyond the scope of this study. We have therefore refined the annotation so it is based on marker genes (SAEndo1: PLVAP^{hi} endo; SAEndo2: ACKR1^{hi} endo) rather than spatial distribution or inferred function.

-A 4-week CCl4 model is rather acute and the fibrosis deposition will be limited. A previous publication (PMID: 35750138) reported Trem2+ cells in an acute MASH model (ldlr-/- mice on 2 weeks of HFC diet), highlighting associated markers as Cd9, Gpnmb, Lgals3 or Cd63. Are the 'SAMs' acute responders rather than fibrosis-associated? This could have a profound impact on the nomenclature/annotation. In addition, how relevant is the CCL4 model (a toxic model) to the different human aetiologies presented in this paper? Either different models should be included to mirror the different aetiologies or be taken out altogether.

We used the 4-week CCl_4 model based on our previous work, where we had significant ECM deposition using the same CCl_4 protocol and were able to study macrophage kinetics during disease regression (Ramachandran P et al, PNAS 2012; PMID: 23100531). Similarly, in this current study, we were able to define the kinetics of the SAMac subpopulations using this model. The reviewer is correct that this is still a fairly short model compared to others, and the level of fibrosis is much less than that observed in advanced human CLD, something which is often a challenge when comparing mouse models to human pathology. As suggested by the reviewer, we have substantially strengthened the links between our human findings and mouse models by expanding our analyses to include additional mouse CLD data. Firstly, given the striking expansion of OLR1+ SAMac2 in human biliary diseases (Extended Data Fig. 4E; Extended Data Fig. 7J, L, N, P), we have now performed scRNAseq on the mouse bile duct ligation (BDL) model. Consistent with the CCl_4 model, BDL also results in expansion of an OLR1+ SAMac2 population (Extended Data Fig. 8C-F). We extended these findings even further by performing multiplex immunofluorescence in an additional mouse model of MASH (CDAHFD) (Extended Data Fig. 8I), again confirming that OLR1+ SAMacs expand across different models of CLD and are distinct from GPNMB+ SAMacs. Hence, accumulation of hepatic OLR1+ SAMacs is a common feature across multiple different mouse models of CLD, which recapitulate the different features of human liver pathophysiology.

Regarding the role of SAMacs in acute response, this is an excellent question. The reviewer is correct that Trem2+ macrophages also accumulate in acute injury in mice, for example in acute MASH as highlighted, and also in APAP as recently shown by the Scott group (PMID: 39862865). Interestingly, the data from the Scott group does suggest some transcriptional differences in liver LAMs/SAMacs between acute APAP and chronic CCl_4, indicating that there may be alterations in SAMac properties dependent on the duration and nature of injury. However, whether there are functional differences between these contexts remains unclear. This would of course be interesting to follow up on in future work, ideally also including human comparative data. Whilst beyond the scope of this current study, we believe our atlas data will support such comparisons in the future, enabling more nuanced annotation of human liver MPs, which can then be compared in abundance and transcriptional profile between different durations and causes of disease.

-Line 254. 'Having identified 2 distinct populations of TREM2+ CD9+ SAMac'. The paper presents expression mainly using dot plots. This is rather confusing as it only shows some enrichment of certain markers in that cluster. This does not mean that all the cells within that cluster express these markers. STAB1 also seems to be expressed in SAMs The paper would benefit from including some feature plots, especially for the main markers (OLR1, TREM2, CD9, etc). What is the percentage of these cells within the myeloid population?

We thank the reviewer for the suggestion. They are absolutely correct that not all cells in a given cluster will express a given marker – this is a recognised issue with scRNAseq data where gene dropouts are common, highlighting the need for clustering to define cell types and states rather than relying on individual genes. Throughout the manuscript, we have shown selected statistically-significant marker genes for each cluster to aid with annotation and interpretation for the reader. We chose dot plots as they can concisely represent the expression of multiple genes across multiple clusters in a space-efficient manner. However, we also included full lists of marker genes for each cluster in Supplementary Tables and readers will be able to utilise the online data browser and downloadable Loupe file to generate feature plots for specific genes of interest.

As suggested by the reviewer, we have now also added feature density plots showing co-expression patterns of TREM2, OLR1, CLEC5A and GPNMB (Extended Data Fig. 7b). SAMac2 demonstrates co-expression of TREM2/OLR1 and TREM2/CLEC5A, whilst SAMac1 demonstrates co-expression of TREM2/GPNMB and TREM2/FABP5, further highlighting that SAMac1 and SAMac2 represent distinct subpopulations of TREM2+ macrophages. In terms of percentages, on average SAMac2 represents 1.36% of the myeloid cells in healthy livers, increasing to 3.78% in CLD. SAMac1 represents 3.11% of the myeloid cells in healthy livers, increasing to 6.70% in CLD.

-Table S11. Differentially expression of SAM1 with SAM2. Looking at the DEGs, one would almost say that this is a comparison between LAMs and SAMs (FABP5, GPNMB, CD36, LPL). Assuming that this is a differentially expression analysis between clusters, it would make more sense to compare TREM2+CD9+ cells from cluster 1 with TREM2+CD9+ cells from cluster 2.

We agree with the reviewer that SAMac1 is more akin to the mouse LAM phenotype, although the transcriptional differences, if any, between LAMs and SAMacs have not been previously described. The reviewer is correct that this is differential expression analysis between clusters. We decided to do this at a cluster level, because reliance on individual genes (e.g. TREM2 or CD9) to select cells for subsequent analysis risks introducing biases due to the potentially stochastic nature of gene dropouts in scRNAseq. However, even when selecting the TREM2+ CD9+ cells from each SAMac cluster and comparing gene expression, we detect similar transcriptional differences in the key markers discussed in this manuscript (Reviewer Fig. 2). There are no differences in the proportion of cells expressing TREM2 and CD9 between the SAMac1 and SAMac2 clusters (Extended Data Fig. 7B).

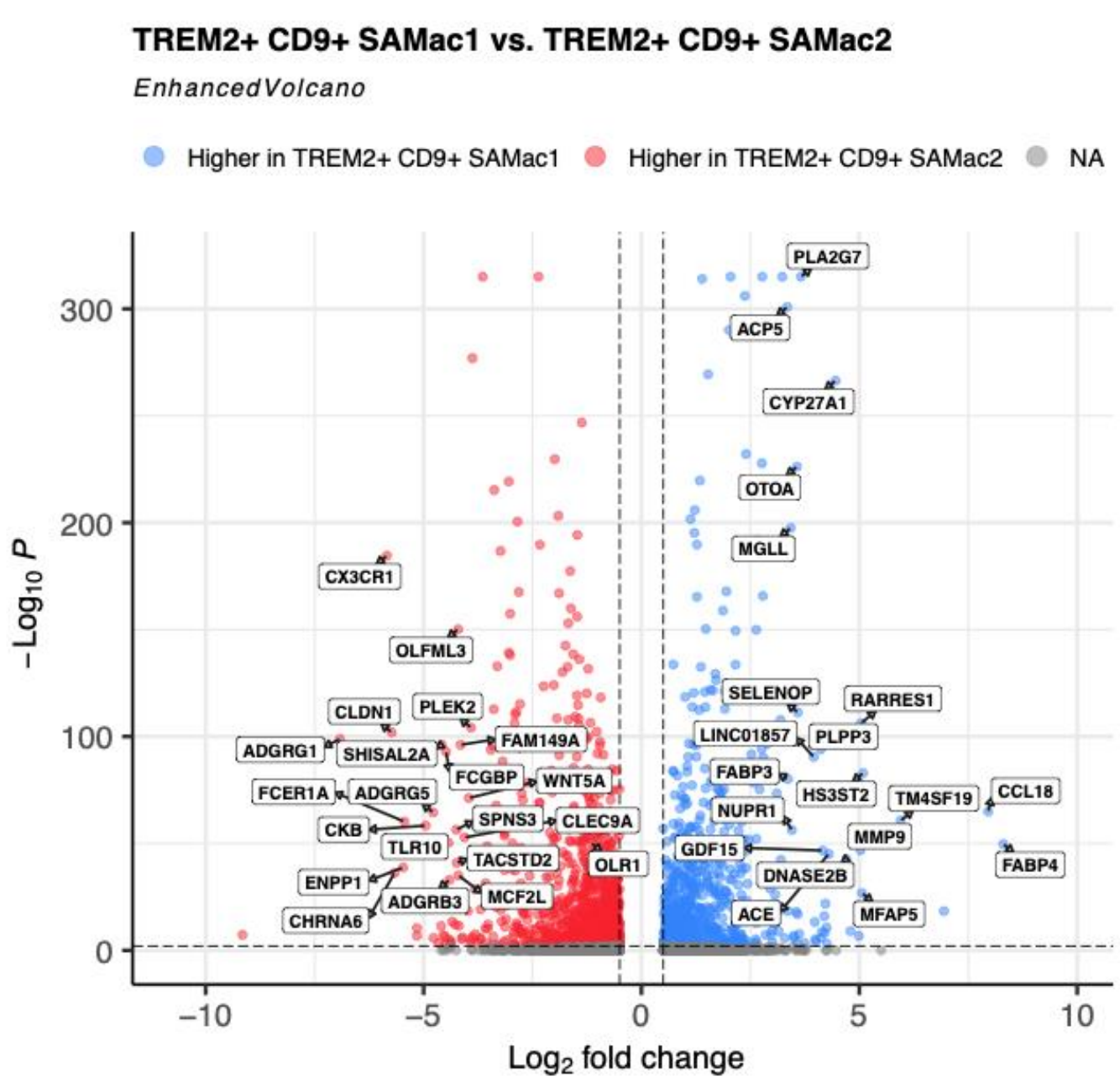

Reviewer Fig. 2: Differential expression analysis between TREM2+CD9+ SAMac2 and TREM2+CD9+ SAMac1, having selected TREM2+ CD9+ cells from each SAMac cluster.

-Line 266 "OLR1+ CD68+ SAMac2 (Fig. 3D; red arrows) were distinct from GPNMB+ CD68+ SAMac1 (Fig. 3D; yellow arrows)". The used antibodies are rather limited and will capture different cell populations as well, making it difficult to make any conclusions. Adding markers to the staining panel, such as TREM2 and CD9, well help to identify SAMs with more certainty. Including different aetiologies and stages reflecting the transcriptomics data would strengthen the paper.

Thank you for this suggestion. We have now conducted a spatial proteomics experiment using a 36-plex antibody panel on the Akoya PhenoCycler-Fusion applied to 46 patient samples. Both OLR1+ and GPNMB+ SAMac subpopulations can be annotated alongside other hepatic cell types (Fig. 3C). Quantification confirms expansion of OLR1+ SAMac2 and GPNMB+ SAMac1 in CLD across different aetiologies (Fig. 3E-F, Extended Data Fig. 7F-G) and cellular neighbourhood analysis demonstrates that both SAMac subpopulations accumulate within the fibrotic niche (Fig. 3H-M). Our deconvolution analysis (see response to Q1) further

confirms the accumulation of OLR1+ SAMac2 with fibrosis stage across different aetiologies of CLD (Fig. 4I, Extended Data Fig. 7J, L, N, P).

Minor
-Statistical significance should be added in the main figures where possible, especially with the MiloR plots.

The beeswarm plots used to represent MiloR results already incorporate a statistical analysis, with only neighbourhoods which are significantly expanded coloured. Non-significant neighbourhoods are coloured in grey. Key findings most relevant to the manuscript, specifically the expansion of SAMac1 and SAMac2 in CLD, were also confirmed using an alternative statistical approach (Extended Fig. 4C). Statistical testing for other analyses are described in the relevant Figure legends, but we are of course happy to clarify any uncertainties.

-In chronic liver disease, zonation is gradually lost. How can the authors explain zonated KCs in cirrhosis? Furthermore, KC have been shown to be mobile within the parenchyma (Peiseler M et al 2023).

The reviewer is correct that we cannot make any comment about KC zonation in this manuscript, as we have not performed any spatial validation to substantiate this. We based the periportal (PP KC) KC annotation on the higher levels of IL-10 expression, similar to the PP KCs described by Miyamoto et al (Nature, 2024; PMID: 38658756). However, we did not prove a spatial location for this population in our data, so have adjusted the annotation to IL10^{hi} KC throughout the manuscript. As the reviewer suggests, KCs could potentially be mobile, which we cannot assess in this study.

-Shiny app. Cholangiocyte markers are expressed in a subset of macrophages and mesenchymal cells. Is this a limitation of the annotation or a technical issue?

We believe this is technical and likely reflects a data scaling issue when visualising genes which are lowly expressed in certain lineages. We do not see significant expression of cholangiocyte markers in MPs or mesenchymal cells, either at a whole atlas level or when focussed on MPs alone (Reviewer Fig. 3).

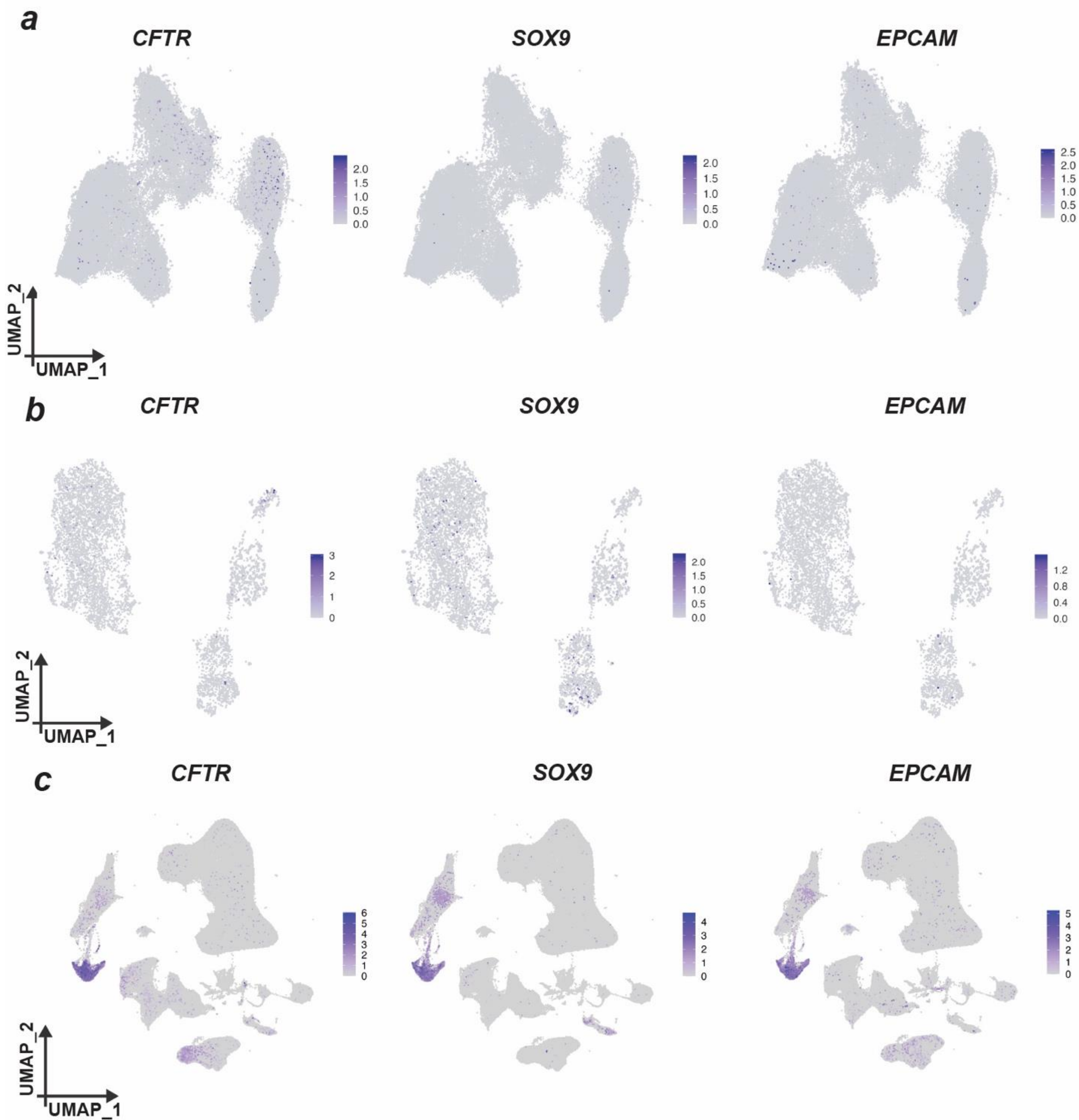

*Reviewer Fig. 3: Feature plots showing expression levels of selected cholangiocyte markers in: (**a**) level 3 MPs; (**b**) level 3 mesenchyme; (**c**) across the whole atlas.*

-OLR1 is widely expressed in different macrophage clusters (Shiny app). In line, OLR1 is widely expressed in different myeloid clusters and high in monocytes (livercellatlas). How does this impact clinical outcome? Are monocytes predictive rather than OLR1+ SAMs?

Within the Level 3 MP atlas, OLR1 is a statistically significant marker gene of 3 clusters – SAMac2, Transitioning Mac and Tissue Monocyte 1 (TMo1) (Supplementary Table 5), but expression is highest in SAMac2. SAMac2 is derived from differentiation of monocytes, most probably via the intermediate transitioning mac state. It is likely that the OLR1 expression is on a continuum between these different monocyte-macrophage states. We believe it to be the OLR1+ SAMacs that are the driver of clinical outcomes

rather than monocytes. Firstly, whilst OLR1+ SAMacs expand significantly in CLD livers, we see no significant expansion of OLR1-expressing monocytes (TMo1) when analysing scRNAseq data (Fig. 2D, Extended Data Fig. 4C). To further assess this, we have performed deconvolution of bulk RNA-seq data from SteatoSITE to estimate cell type proportions. We identify an expansion of OLR1+ SAMac2 with fibrosis stage and demonstrate that a higher proportion of OLR1+ SAMacs is associated with adverse clinical outcomes (Fig. 4I, L, N). However, we do not see a significant increase in the proportion of tissue monocytes with fibrosis stage (Reviewer Fig. 4a) or an association between TMo proportion and adverse outcomes (Reviewer Fig. 4b), further suggesting that it is the SAMac population that is the key driver of disease pathogenesis.

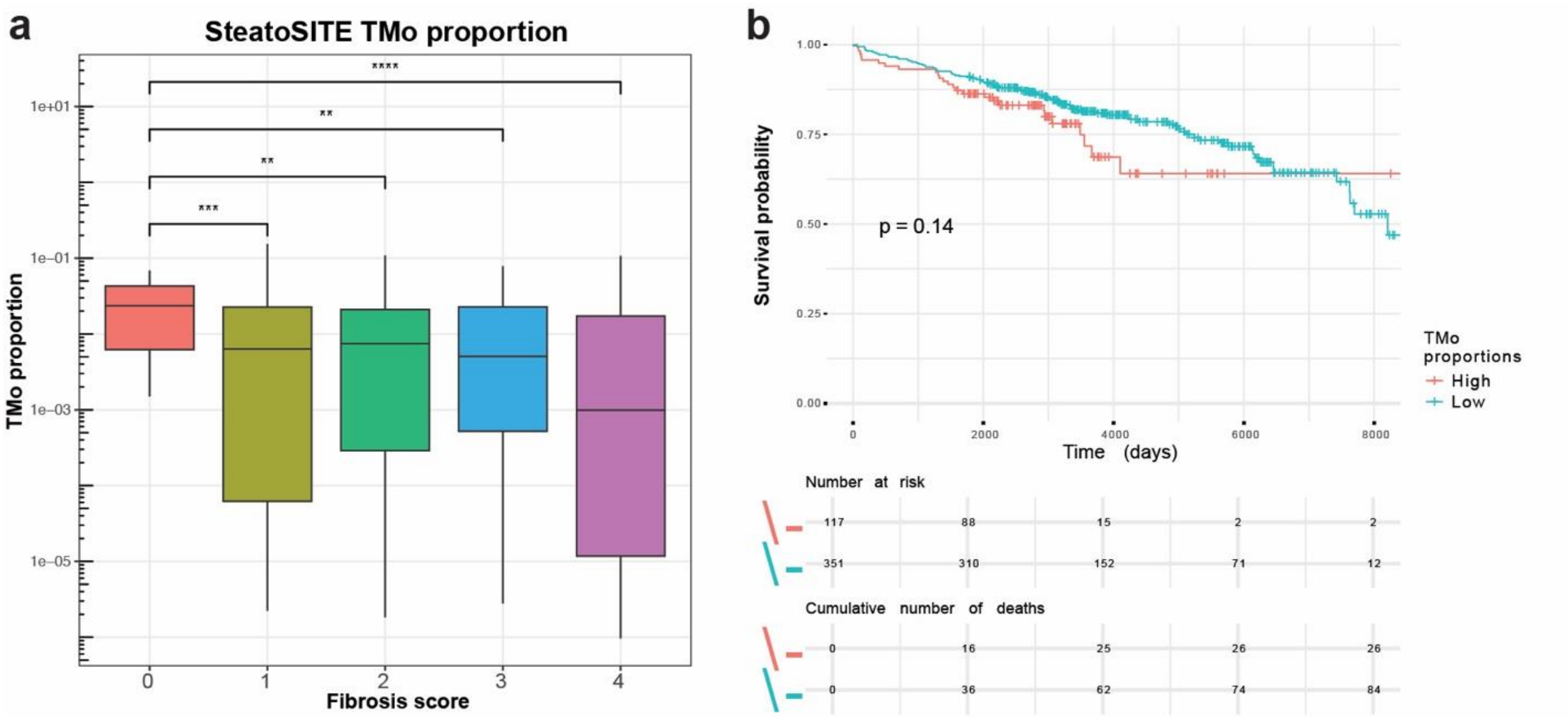

*Reviewer Figure 4: (**a**) Deconvolution of SteatoSITE bulk RNA-seq data using scRNAseq MP atlas, showing estimated TMo proportions according to histological fibrosis stage. Kruskal-Wallis test with pairwise Wilcoxon and BH correction, **p<0.01, ***p<0.001, ****p<0.0001. (**b**) Biopsy cases from the SteatoSITE dataset with available RNA-seq and deconvolution data were used for time-to-all cause mortality analysis. TMo proportions were used to divide cases into low and high risk of death (Kaplan–Meier estimator curves with log-rank test p-value shown).*

-The MASLD data set from Gibben C et al 2024 indicates a poor overlap between TREM2 and OLR1. Is there an aetiology impact?

We believe this to be a technical difference between studies. The data from Gribben et al. was single-nucleus RNA-seq (snRNAseq) as opposed to single-cell RNA-seq (scRNAseq) in this study. SnRNAseq is much less sensitive for cytoplasmic transcripts, especially in immune cells, so direct comparisons between expression patterns of individual genes between modalities tend to be challenging to interpret. A longer-term aim for the field should be to fully integrate published snRNAseq data, including the data from Gribben et al, with the scRNAseq atlas we present, to allow a more comprehensive assessment of the differences between modalities.

We don't believe this to be a MASLD-specific aetiology difference because:

1) **OLR1 increases with fibrosis stage in 2 independent MASLD cohorts (Fig. 4G and Extended Data Fig. 7I)**
2) **Deconvolution analysis confirms expansion of OLR1+ SAMac2 in MASLD patients (Fig. 4I and Extended Data Fig. 7J)**
3) **Our newly added biopsy scRNAseq data confirms upregulation of OLR1 in SAMacs from MASLD patients (Fig. 4A-D)**
4) **Our new spatial proteomic data demonstrates the accumulation of OLR1+ SAMacs in the fibrotic niche of patients with MASLD (Fig. 3G-M, Fig. 4F, Extended Data Fig. 7F).**

-Table S3: level 2 annotations. Numbers for the repeats are added to column 1 (e.g. TREM21 and TREM2 gene IDs are used). Best to remove column 1.

This has been corrected

-Receptor ligand interaction overstated. Supp Table 15 suggests a wide interactions with different cell types. Integrins are widespread receptors for ECM and can interact with a multitude of cell types. Moreover, some of the highlighted markers in the myeloid cells will be rather lowly lowly expressed on RNA level, making the biological relevance of these interactions questionable. For instance, CCL20 is mainly expressed by epithelial cells, complement is mainly produced by hepatocytes.

We appreciate this comment. We have purposely performed a focussed interactome analysis between SAMacs and mesenchymal cells here to align with the narrative of our manuscript. A broader interactome analysis across the atlas, ideally combined with spatial context, will be informative in refining the specificity and biological relevance of particular interactions. We have now acknowledged this point in the discussion (Page 16).

-Format supp tables. Some genes are annotated as dates.

This has been corrected

Reviewer #2 (Remarks to the Author):

This study by Papachristoforou, Kong, Colella et al. aims at providing an extended cellular atlas of chronically diseased livers based on multiple, previously published, single cell transcriptomic datasets. The authors then identified OLR1 as a marker of a specific scar-associated macrophage population particularly enriched in CLD livers, and explored the functional implications of OLR1 on the macrophage – fibroblast interactions.
The manuscript opens by describing the integration and re-analysis of previously published single cell transcriptomic datasets. The analyses appear very well done to the Reviewer, with the highest standards in terms of data quality and data sharing. For this, the Reviewer wants to congratulate the authors. This aspect truly makes the manuscript timely and this will serve as an extremely valuable resource for the field. Nonetheless, the aspects on OLR1 validation as a potential therapeutic target appear less convincing and additional attention is required on the related experiments. Overall, the Reviewer is convinced by the data that there are SAMac1 and SAMac2 cell subsets that are relevant for further studies and characterization in CLD, yet the importance of OLR1 as a stand-alone therapeutic target remains to be corroborated. Could instead OLR1 be one of several markers identifying SAMac2, and that potentially other genes or proteins should be co-targeted for an effective response in vivo and in vitro?

We are grateful to the reviewer for highlighting the utility of our work to the field. In response to the reviewer's comments we have undertaken additional functional work to support the potential for modulating OLR1 as a therapeutic target in CLD (see below). The reviewer is absolutely correct that OLR1 is likely to be one of several markers which could promote pro-inflammatory pro-fibrotic functions of SAMac2. Another example would be CLEC5A, which is specifically expressed by OLR1+ SAMac2 (Extended Data Fig. 7B) and has previously been shown to promote myeloid cell activation and inflammatory mediator production in other disease contexts (e.g. PMID: 22005300, PMID: 20212065). Targeting of CLEC5A (or other proteins) alongside OLR1 could potentially have additive anti-inflammatory antifibrotic effects and warrants exploring in the future. We have acknowledged this point in the Discussion section.

The reference list seems appropriate. The text is very well written and the abstract, introduction and conclusions reflect the content of the manuscript.

Specific comments
- Considering this relative low number of patients and heterogeneities in disease etiologies, individual patient characteristics may have profound impact on the pool of "rare" cells herein described. This comes with additional complexity due to study variations. While this is well described into the manuscript, how can the authors take this more into considerations? Are the identified SAMac2 more prominent in some datasets compared to others?

The reviewer is correct that the meta-analysis of such scRNAseq data poses challenges due to study variations, necessitating the painstaking integration and annotation that we have performed. Individual patient characteristics could contribute to variability, and study-specific differences in cell isolation protocols result in differences in the abundances of different cell types captured. It should be noted that the statistical method for identifying changes in cell type abundance between conditions using the Milo package (PMID: 34594043), incorporates modelling to account appropriately for batch effects of varying magnitudes across complex experimental designs, controlling false discoveries and ensuring that only robust cell composition changes are identified. We have used this model as the basis for identifying disease-expanded populations such as OLR1+ macrophages. This expansion is evident even when looking at individual CLD samples across included scRNAseq studies (Reviewer Fig. 5). We would also stress the importance of not solely relying on scRNAseq data to study cell type abundance differences, but also validating changes in key pathogenic populations using completely independent samples and methodologies.

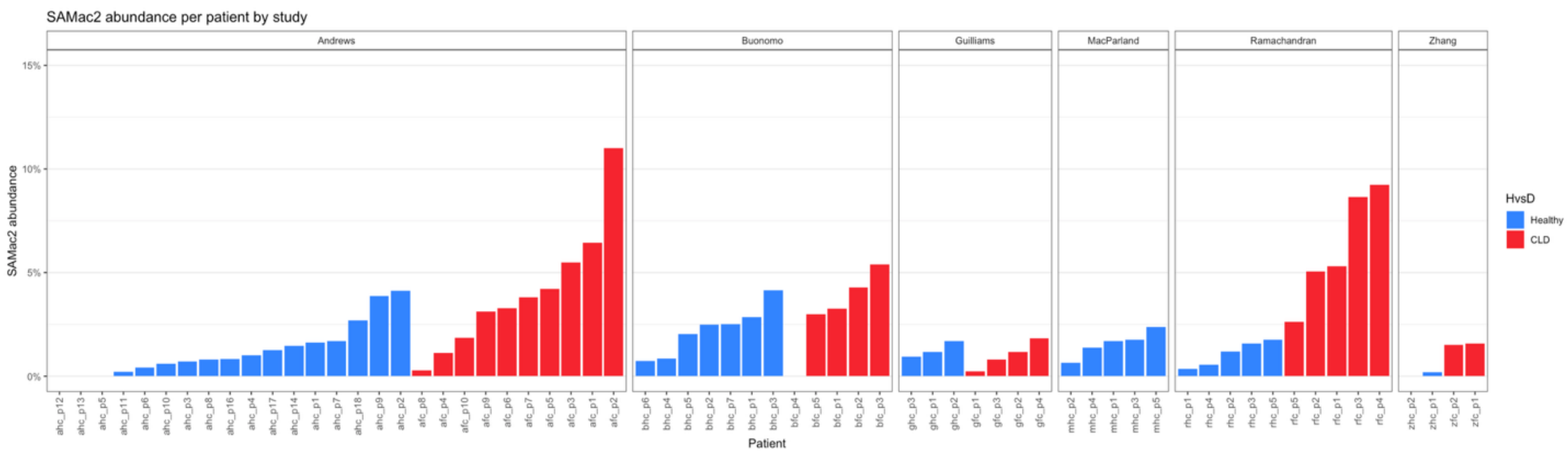

Reviewer Fig. 5: SAMac2 abundance as a percentage of total mononuclear phagocytes, grouped by original study. Each bar represents an individual patient. Bar colour reflects healthy (blue) vs CLD (red) patients.

With regard to the rare cells, the reviewer is absolutely right that the impact of interindividual or study-specific variability is likely to be more pronounced, as exemplified for basophils in Reviewer Fig. 6. We have added to the field by identifying markers for rare cell type identification to aid more in-depth study, but have not focussed our analyses on changes in rare cells between disease states or aetiologies. The need for extra validation of such changes should be considered by any researcher wishing to pursue this.

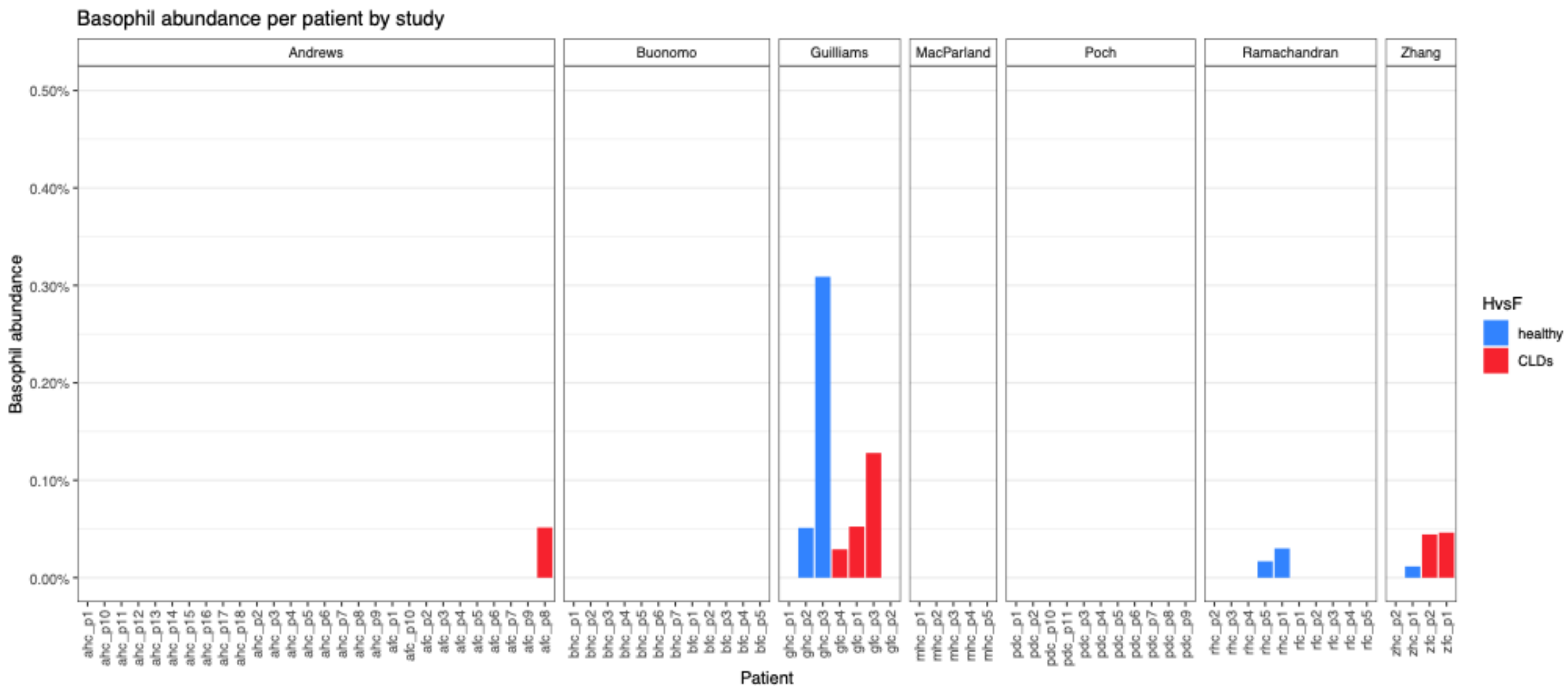

Reviewer Fig. 6: Basophil abundance as a percentage of total cells, grouped by original study. Each bar represents an individual patient. Bar colour reflects healthy (blue) vs CLD (red) patients.

- Maybe this Reviewer missed the information, but could the authors provide some information on how efficient their (and the ones from earlier studies) method for single cell RNAseq is? Meaning, how many cells are effectively analysed per amount of tissue? And could the authors provide this value for each individual sample for QC and comparison?

This is an interesting question but unfortunately is not possible to address in the data presented in the atlas. Generally, there are an excess of cells following tissue dissociation, the exact number dependent on the dissociation protocol used and the size/freshness of the tissue. A set number of cells are then loaded for barcoding and sequencing, according to reagent availability, budget and desired sequencing depth.

However, for our newly added liver biopsy scRNAseq data (Fig. 4A), we generally obtain around 35,000 single cells per patient from a 5mm 16G core of liver tissue. We individually hybridize samples then pool together 20,000 to 30,000 cells from each sample and load approximately 200,000 total cells on a lane of the 10X Gemonics chip, with the aim of sequencing approximately 10,000 cells per sample.

- Fig.1D is particularly valuable in understanding the specificities of each dataset used. Could the authors elaborate on how the discrepancies in cell types could be explained? How much does that influence the following analyses?

The differences in cell type composition between studies are generally due to the different cell isolation and enrichment methods used in each original study. The statistical model used to assess differential cell abundance (Milo package) accounts for batch effect and differences in cell type enrichment between samples, so where there is adequate representation of that cell lineage across studies, no impact on these results is anticipated. However, for certain cell lineages (e.g. hepatocytes) the vast majority of cells originate from a subset of studies (see Extended Data Fig. 6C). In such cases, we have elected not to study changes in cell type composition across the atlas due to study-related batch effects.

- The transcriptomic data pointing towards OLR1 seems very clear. However, the OLR1 staining data presented in Fig.3C/3D and Extended Fig.7A is moderately convincing and additional validation at the protein level would appear important, for OLR1 to be considered a valid therapeutic target. Can the authors provide additional images with a better resolution, and rule out unspecific signal? How many positive cells have been observed in the tissues? Is this observation consistent between different etiologies of CLD? The Reviewer has similar questions on the flow cytometry shown in Extended Fig. 9C. The population of OLR1+ macrophages seems very small and not well defined in the flow cytometry results, meaning there seems to be a gradient of OLR1 expression across SAMac1 and SAMac2. This may question the specificity of an OLR1-targeted therapy if, at least at the protein level, there does not

seem to be strong differences in levels of OLR1 expression (if we assume the protocols used for IF and FC are robust).

We thank the reviewer for highlighting the importance of further protein validation of increased OLR1+ cells in CLD livers. Consequently, we have conducted additional experiments and analyses to address this point. Firstly, we have performed a spatial proteomic analysis using a 36-plex antibody panel on the PhenoCycler-Fusion. Using this approach, we identified OLR1+ SAMacs at the protein level (Fig. 3C, I), demonstrated that this population expands in CLD liver tissue of different aetiologies (Fig. 3E, Extended Data Fig. 7F), and showed that it is spatially localised in the fibrotic niche (Fig. 3I-L, Fig. 4F). On average, we identify 66.5 +/- 8.63 OLR1+ SAMacs per mm^2 in CLD livers vs 3.68 +/- 0.636 in healthy human liver. Additionally, we have performed flow cytometry on myeloid cells from human liver, indicating expansion of TREM2+ OLR1+ SAMacs in CLD livers (Extended Data Fig. 7C)

To extend the protein level validation of murine OLR1+ SAMac expansion, originally shown by flow cytometry (FC) in Fig. 5G-I, we have now optimised multiplex immunofluorescence (IF) for SAMac subpopulations in mouse liver tissue (Extended Data Fig. 8G). Using this approach, we confirm expansion of OLR1+ TREM2+ SAMacs in mouse CCl_4 and CDAHFD models of CLD (Extended Data Fig. 8H-I) and demonstrate spatial localiation adjacent to areas of collagen 1 deposition (Extended Data Fig. 8G).

We have used validated and previously published protocols for IF and FC, but depending on the antibody clone and epitope there maybe differences in the sensitivity of OLR1 protein detection. However, the fact we have confirmed our findings at a gene and protein level in two species, gives us confidence in the validity and reproducibility of our data. The reviewer is correct that because SAMacs are formed by the recruitment and differentiation of blood monocytes and the fact that there is likely to be plasticity within the liver macrophage compartment, there will be a gradient of expression of OLR1 and other markers between monocytes, transitioning macs and SAMacs. Indeed, there may be plasticity between the SAMac1 and SAMac2 states, something which we intend to explore in future work.

- There is a typo in the title of Fig. 5I.

Thank you for highlighting the typo. This has been corrected.

- May the authors describe the overall morphology of the spheroid in Fig.6B? And provide a scale bar, as well as potentially additional images and characterization of the in vitro culture system.

We have added a scale bar. The morphology of this spheroid system has been previously described in more detail in PMID: 33007774 and PMID: 40069876.

- There is a typo page 15 “of the of the CSF1R inhibitor Pexidartinib”.
Thank you for highlighting the typo. This has been corrected.

- The effects of the siRNA OLR1 appear moderate, so does the use of the conditioned media on LX2 gene expression pattern. Besides, additional experiments would be needed to confirm LX-2 are less fibrogenic in this set-up. One may consider evaluating the effect of siRNA OLR1 on monocytes or macrophages prior to seeding them into the multilineage spheroids?

We thank the reviewer for the excellent suggestion. We have now supplemented our LX2 data with additional experiments to assess the impact of OLR1 knockdown on fibrogenic activity in a primary human multicellular 3D MASH model, comprising macrophages, HSCs and hepatocytes (PMID: 39605182). Treatment of these spheroids with OLR1 siRNA attenuates OLR1 expression and significantly inhibits fibrogenic activity at both the gene and protein level (Fig. 7I-L). We believe these data strengthen our conclusions about the functional impacts of OLR1 modulation.

- The Reviewer does not understand why a CSF1R was used to block OLR1+ macrophages. Is that a specific approach? Could the authors instead use a blocking antibody against OLR1?

We appreciate this comment and apologise for any confusion. The purpose of the CSF1R blockade in spheroids following the addition of patient monocytes was to deplete the MDMs as a whole, to demonstrate that this cell type could promote fibrogenic activity. We did not intend to infer any conclusions about specific modulation of OLR1 in these experiments, so have softened the language around this. As suggested by the

reviewer, we have now included additional functional experiments with direct targeting of OLR1 in primary human liver MASH spheroids (Fig. 7I-L).

Reviewer #2 (Remarks on code availability):

The Reviewer does not have the expertise to review the code.

Reviewer #3 (Remarks to the Author):

This is an important article from leading experts in the field with some intriguing results.

General comment #1: The main value of the paper is the Human Single-cell Atlas of healthy vs diseased livers. This work builds on the prior Nature paper (from 2019) by these authors. However, there is a note of caution. In their initial Nature paper, the authors labeled a population of “scar-associated macrophages” (SAMs) as “pro-fibrogenic). A large series of subsequent articles, mainly with mouse models, demonstrated clearly that most of these SAMs are anti-fibrotic and help to counteracting inflammation/fibrosis. In the current work, the authors show that SAMs have different populations, of which some are profibrogenic whereas others have a more anti-fibrotic phenotype. The functional experiments supporting this assumption (which is grounded on the transcriptomic and spatial analyses) are comparatively weak, and I am concerned that the authors over-interpret their findings again (similar as in the 2019 Nature paper).

The reviewer makes a great point about the controversy in the literature regarding the dichotomous functional roles of SAMacs in fibrosis, following our original description of this population in 2019. Our previous human functional data and data from other groups (e.g., the Fabre group; PMID: 37027478) suggested that SAMacs have a pro-fibrogenic role, whilst other subsequent murine studies (e.g., PMID: 35750138, PMID: 39172787, PMID: 37028770) have indicated that these cells have an anti-inflammatory/antifibrotic role. This is exactly the controversy that we have tried to resolve in this manuscript. Due to the similarities between SAMac1 and SAMac2 and the potential plasticity in this compartment, it is only by large-scale single cell atlassing that we have been able to resolve two subtypes of TREM2+ SAMac with distinct transcriptional programs. One of the main challenges with the original definition of SAMacs was the lack of understanding of how to selectively target or modulate the pro-fibrogenic functions. Due to the specificity and conservation across tissues, much of the focus has been on TREM2 modulation, which has transpired to be a molecule with anti-inflammatory functions in the liver. However, our data shows that TREM2 is actually equally expressed by both subtypes of SAMac (Extended Data Fig. 7A-B), so targeting TREM2 is not specific to the population with a pro-inflammatory phenotype. In contrast, expression of OLR1 (and other SAMac2 markers such as CLEC5A) is more restricted to pro-inflammatory TREM2+ SAMacs, suggesting the capacity for more selective therapeutic targeting. In this revised manuscript, we have added further functional data confirming OLR1 as a candidate target to modulate pathogenic SAMac functions, but of course the reviewer is right that more work needs to be done to fully understand the functional consequences of our findings in different disease contexts. However, as with our 2019 paper, we hope that our novel description of SAMac heterogeneity provides the basis for future studies in this area over the forthcoming years.

General comment #2: The authors apparently built their Human Single-cell Atlas entirely on previously published data-sets. This comes with a lot of challenges (different cell isolation protocols, different sequencing depth, heterogeneous patient sources, limited clinical data). In addition, it dampens my enthusiasm regarding the novelty of the data. By the way, the fact that no original scRNA-seq data from human liver were generated for this study is a bit hidden and only apparent from a supplementary table. Also, for linking marker expression (e.g. OLR1) to outcome, the authors use an already published data-set (Steato-SITE), which limits originality of the work.

We are sorry that the reviewer felt the data sources for atlas generation were a bit hidden. We always strive to make our work as transparent and interpretable as possible. We did highlight in Fig. 1A which datasets were used to generate the atlas and also included this information in the data availability statement, with relevant citations. We are happy to reiterate this point elsewhere if the reviewer and editors feel this is helpful.

There are, of course, many challenges in the integration of different data sources as highlighted by the reviewer. This requires painstaking QC, integration, clustering and manual annotation to ensure that findings are biologically robust. The level of expertise needed to complete this type of analysis, to place the data in the relevant biological context and to make it accessible and interpretable to other researchers from a

biological rather than computational background is precisely where the originality of this aspect of our manuscript lies. The reviewer is right that we also used previously published SteatoSITE bulk RNA-seq data – this is the only available dataset with large-scale bulk RNA-seq and linked clinical outcomes, so is unique in being able to link OLR1 gene expression or SAMac expansion with clinical disease trajectory. It is not the SteatoSITE data itself that is novel, but rather the application of these data to understand molecular drivers of adverse clinical outcomes that will be of interest to the field.

To further address the reviewer's concerns about originality, we have generated and added additional datasets to the original manuscript. These novel 'omics datasets include:

1) **Human liver needle biopsy scRNAseq comparing healthy vs MASLD patients, using a newly developed protocol**
2) **Human liver spatial proteomic data employing a 36-plex antibody panel across 46 patient samples using the Akoya PhenoCycler-Fusion**
3) **Mouse liver and blood scRNAseq data from the bile duct ligation (BDL) model**
4) **Mouse liver and blood scRNAseq at multiple timepoints from the mechanistically divergent CCl_4 model**
5) **Mouse liver and blood scATACseq data from the CCl_4 model**

We hope this combination of using published and novel data to answer an important biological question and identify novel therapeutic targets highlights the originality of this body of work.

Additional specific comments:

3.The authors identify OLR1+ CD68+ SAMac2 (inflammatory profile) vs. GPNMB+ CD68+ SAMac1 (anti-inflammatory) in diseased livers as populations of CD9+ TREM2+ SAMs. I am not convinced that the functional implications of these cells are clarified by the authors. For instance, Fig. 6 does only include experiments on reducing OLR1, but not on the other population. For a better functional understanding, one would need either selective depletion models in mice (ideally in clinically relevant MASH-fibrosis models, depleting either one of the two SAM subsets) and convincing in vitro settings, in which liver-educated primary SAM populations are co-cultured with primary hepatocytes and primary HSCs. The work with cell-lines (LX2, H9 spheroids) and the lack of clear experimental conditions comparing SAMac1 vs SAMac2 in a multicellular system is not sufficient.

We agree with the reviewer that a useful experiment would be to selectively deplete either SAMac1 or SAMac2 in an *in vivo* setting to further study their functional role. Unfortunately, the necessary tools to achieve this are not currently available. Both SAMac1 and SAMac2 populations derive from circulating monocytes, so any attempts to block recruitment or deplete monocyte-derived cells would result in the loss of both populations, whilst more general macrophage depletion strategies, such as CSF1R inhibition or liposomal clodronate, lack specificity. There is also the possibility of plasticity between the SAMac2 and SAMac1 states, which needs to be further explored using fate mapping approaches before *in vivo* modulation can be fully interpreted.

In response to the reviewer's suggestions we have better studied the differences between SAMac1 and SAMac2 in primary human MDMs by defining which *in vitro* polarisation conditions promote SAMac1 vs SAMac2 transcriptional programs, highlighting that the OLR1+ SAMac2 state is driven by pro-inflammatory stimuli such as TPP, whilst the GPNMB+ SAMac1 state is favoured by stimuli such as palmitic acid or glucocorticoids (Fig. 7A-E, Extended Data Fig. 11H). These distinct macrophage polarisation states appear to have differing effects on HSC activation (Extended Data Fig. 11I). These data, coupled with our cross-species scRNAseq and mouse scATACseq, all point towards SAMac1 and SAMac2 reflecting functionally distinct macrophage polarisation states.

We also developed a human liver flow cytometry panel to distinguish OLR1+ and OLR1- TREM2+ SAMacs (Extended Data Fig. 7C). However, we are currently unable to FACS-isolate sufficient numbers of SAMacs for robust *in vitro* assays such as co-cultures – this should, of course, be a focus of future studies, which also incorporate detailed analysis of stability of macrophage phenotypes *ex vivo* following isolation.

4. The authors do not show clear data whether the both CD9+ TREM2+ SAMs, OLR1+ CD68+ SAMac2 (inflammatory profile) vs. GPNMB+ CD68+ SAMac1 (anti-inflammatory), have a distinct spatial pattern in the liver (mouse and human). The few "representative pictures" cannot clarify this, a statistical analysis of the spatial organization is missing. In this regard, it would be important to show that the balance of the two populations is affected in progression vs regression conditions.

We thank the reviewer for the helpful suggestion. Accordingly, we developed a custom 36-plex antibody panel and performed spatial proteomic analysis on 46 human livers using the Akoya PhenoCycler-Fusion. We have annotated OLR1+ SAMacs and GPNMB+ SAMacs and confirmed that both populations expand in CLD livers (Fig. 3C-F), across different aetiologies (Fig. 3G, Extended Data Fig. 7F-G). Cellular neighbourhood analysis confirms that both SAMac subpopulations accumulate in the human liver fibrotic niche (Fig. 3H-M).

In mouse, we have optimised a multiplex immunofluorescence panel, showing accumulation of distinct TREM2+OLR+ and TREM2+GPNMB+ SAMacs adjacent to areas of collagen deposition (Extended Data Fig. 8G-I). Both populations decline during fibrosis regression (Extended Data Fig. 8H).

5. One prerequisite for studying functional consequences of macrophage populations in mice is that they are conserved across species. The authors show this for the CCl4 mode, but this certainly limited translational value. Do these populations also occur (and if so, when and where) in MASH-fibrosis mouse models? Interventions in such models would be more informative.

In response to the reviewer's suggestion to broaden the translational value of our observations, we have confirmed expansion of OLR1+ macrophages in additional mouse models of CLD. Due to the notable expansion of OLR1+ SAMacs in human biliary diseases (Extended Data Fig. 4E; Extended Data Fig. 7J, L, N, P), we performed scRNAseq on the mouse BDL model, showing that both SAMac subpopulations also expand in this context (Extended Data Fig. 8C-F). Furthermore, multiplex immunofluorescent staining confirms that both OLR1+ and GPNMB+ SAMacs expand across different mouse models of CLD, including the CDAHFD MASH model (Extended Data Fig. 8I), and are spatially located adjacent to areas of collagen deposition (Extended Data Fig. 8G).

6. The siRNA-silencing experiments with THP-1 cells are not sufficient to convince me that targeting OLR1 in liver macrophages would be a useful strategy. An inducible genetic depletion in fibrosis models in mice or the selective targeting of primary human SAMs from liver are missing.

In our revised manuscript, we have strengthened the functional data demonstrating that modulating macrophage OLR1 expression is a potential therapeutic strategy for liver inflammation and fibrosis. In addition to THP-1 cells, targeting OLR1 in primary human monocyte-derived macrophages isolated from patients with cirrhosis, using either siRNA or antibody blockade, reduced activation of hepatic stellate cells (Fig. 7G-H). Furthermore, in a human spheroid 3D-MASH model containing primary human hepatocytes, macrophages and hepatic stellate cells, genetic targeting of OLR1 abrogated fibrogenic activity at both the gene and protein level (Fig. 7I-L).

We agree with the reviewer that an inducible genetic depletion model *in vivo* would be ideal; however, the tools to achieve this do not currently exist. It should be noted that due to the kinetics and potential plasticity of MDMs and SAMacs in a dynamic inflammatory context, inducible genetic depletion may well be challenging and will require both new transgenic tools and a significant amount of validation work. This should be a focus of further work, both for the study of CLD but also fibroinflammatory disorders in other organs where SAMacs also play a role.

Reviewer #1 (Remarks to the Author):

Congratulations on the substantial improvements made to the manuscript. The revisions have clearly strengthened the study, and the overall quality has increased. I only have a few remaining comments, mainly related to the spatial proteomics and to explicitly acknowledging limitations in this study.

Thanks to the reviewer for the suggestions which really helped us improve the study. We have addressed the comments below.

1. First, the newly generated human scRNA-seq MASLD cohort does not appear to be accessible via GSE298719 as indicated. If this dataset has been previously published, please provide the appropriate reference.

These are new data which have not previously been published. Apologies for the oversight in not adding these to a GEO submission prior to our last revision. The FASTQ files and Cellranger outputs from these new human scRNAseq samples will be added to GSE298719 prior to publication.

2. Regarding my previous comment on cell cluster annotation, I agree with the authors' decision to avoid labeling these cells as LAMs. However, the current annotation of SAMac appears to rely on the increased expression of a limited number of genes. Importantly, this population may reflect disease activity rather than fibrosis-specific macrophages, as also suggested by the acute CCL4 mouse model. In the referenced dataset (GSE135251), OLR1 and TREM2 expression are more strongly associated with disease activity (NAS score) rather than fibrosis stage. Given this, the limitations of the current nomenclature and interpretation should be more explicitly discussed. Why SAMacs and not disease activity-associated?

Thanks for the suggestion which we agree with. We have added a section to the discussion explicitly describing the limitations of the current nomenclature and the need for further studies in different contexts and durations of injury to determine differences (if any) between LAMs, SAMacs and other disease-associated macrophage states.

3. For the spatial proteomics data, the strategy used to annotate cell populations is not clearly described.

We have used a combination of iterative unsupervised clustering and supervised analyses to generate the annotations, using a similar analytical workflow to that which was recently developed for analysis of CODEX data (DOI: 10.1016/j.cell.2024.04.013). We have added this reference and more detail to the methods section describing our approach. Analysis code will also be available on the Github repository related to the manuscript.

The detection of GPNMB in neutrophils is unexpected and not supported by the scRNA-seq data, suggesting possible annotation inaccuracies or autofluorescence artifacts. What background substration approaches have been implemented?

In the PhenoCycler-Fusion (PCF) platform, autofluorescence subtraction is performed in an automated manner by spectral unmixing within the InForm software package. Blank imaging cycles (with only DAPI and no added fluorescent reporters for antibody detection) are included at the start and the end of each run and used to calculate the autofluorescence profile of the tissue, which is then subtracted from the fluorescent images prior to the subsequent analyses. Of note, the imaging platform is multispectral which improves the differentiation of tissue autofluorescence from genuine fluorescent signals. We also included an autofluorescence quenching step prior to imaging, using a photobleaching protocol as described by Akoya (https://www.akoyabio.com/wp-content/uploads/2020/07/Customer-Demonstrated-Protocol-Autofluorescence-Quenching-Mar2020.pdf).

We don't believe the GPNMB detected in neutrophils represents autofluorescence. GPNMB staining was imaged on the AlexaFluor647 fluorophore and none of the other antibodies imaged on the same fluorophore across different imaging cycles exhibit this staining pattern. The reviewer is correct that annotation inaccuracies or lateral spillover from neighbouring cells can sometimes explain unexpected staining patterns. However, we have carefully reviewed the images and don't believe GPNMB expression in neutrophils is due to annotation inaccuracies or lateral spillover. As shown in Reviewer Figure 1, cells which are positive for neutrophil markers (CD15 and MPO) and negative for macrophage markers (IBA1 and CD68)

show low level expression of GPNMB (white arrows). We therefore believe this to be genuine low-level staining.

GPNMB protein has been identified in mouse neutrophils before (PMID: 38566120), but the reviewer is correct that GPNMB expression is not detected in neutrophils in our human scRNAseq data. ScRNAseq can be sparse, especially in certain cell types like neutrophils with low amounts of RNA, resulting in potential discrepancies between gene and protein expression. GPNMB protein can also be transferred between cells via extracellular vesicles (EVs) (DOI: https://doi.org/10.1038/s41593-026-02317-w), whilst neutrophils are known to be capable of scavenging macrophage-derived EVs via endocytosis (PMID: 40448602). It is therefore possible that the GPNMB protein expression detected in some neutrophils represents uptake of macrophage-dervied GPNMB in the fibrotic niche. Of course further studies will be needed to confirm this hypothesis in the liver and to assess the functional relevance of it.

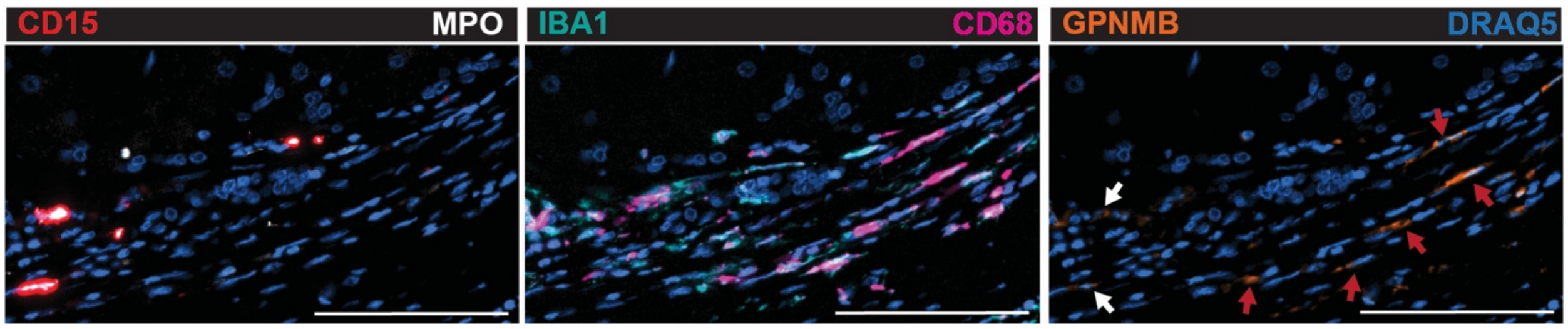

Reviewer Figure 1: Example PCF multiplex staining image showing neutrophil markers CD15 and MPO (left), macrophage markers IBA1 and CD68 (middle) and GPNMB (right). White arrows indicate GPNMB+ cells co-expressing CD15 and MPO. Red arrows indicate GPNMB+ cells co-expressing IBA1 and CD68. Scale bar=100um

Furthermore, it remains unclear how SAMacs were defined at the protein level. Based on the supplementary panel, one would assume a TREM2+CD9+ definition. However, TREM2 is not included in the panel shown in Fig. 3c. If this omission is due to technical constraints, then the annotated population likely represents OLR1+ macrophages rather than bona fide OLR1+ SAMacs. This distinction is important, as it limits the strength of the conclusions and may lead to overinterpretation. It would be more accurate to describe cells strictly based on the markers that were directly measured, rather than using an unclear annotated population.
As suggested by the reviewer during the first revision, we attempted to add a custom conjugated TREM2 antibody to our spatial proteomics panel. Unfortunately, this did not yield consistent staining results in the PCF platform, so we were unable to include these data in our final PCF panel and subsequent analyses. We annotated the OLR1+ and GPNMB+ macrophages as SAMacs in the PCF data based on CD9 expression, but the reviewer is correct that the number of markers included in the PCF panel are not currently sufficient to definitively distinguish OLR1+ or GPNMB+ SAMacs from other OLR1+ or GPNMB+ macrophages. Therefore, as suggested by the reviewer, we have amended the annotation for this part of the study to OLR1+ Mac and GPNMB+ Mac and included this point in the discussion.

The spatial proteomics has been done on 10 healthy and 36 CLD sections. The underlying aetiology and, more importantly, the stage of the disease has not clearly been stated or shown. As it seems now, the quantification heavily relies on transplanted material or resected material, and not on needle biopsies.
Thanks. A better description of these cases has been added to the text. The reviewer is correct that the majority of CLD cases for spatial proteomics analysis were from explants.

Additionally, Fig. 3H does not show enrichment of GPNMB+ SAMacs in hepatocytic regions, which appears inconsistent with Fig. 3M and should be clarified.
Fig 3H shows enrichment (plotted as Log2 overexpression) of different cell types in each computed niche relative to other niches, as described in the original scNiche manuscript (https://doi.org/10.1038/s41467-025-57029-9). An enrichment score of 0 does not mean that the cell type is completely absent in this niche, rather that it is not enriched in this niche compared to others. In contrast Fig 3M shows absolute cell counts per mm2 tissue in each niche. For GPNMB+ Macs, these are present in the hepatic niche but are significantly enriched in the ductular and fibrotic niches when compared to the hepatic niche.

It is also unclear how ductular versus fibrotic regions were distinguished, as these compartments often overlap. The

inclusion of periportal hepatocytes within ductular areas may partly explain the observed differences and should be addressed.
Niches were identified using the scNiche methodology (https://doi.org/10.1038/s41467-025-57029-9). For each annotated cell in the spatial dataset, this algorithm assesses the cellular composition of the local neighbourhood, the cellular proteomic profile and the neighbourhood proteomic profile, implemented in a multiple graph autoencoder, to identify consistent cellular niches across all of the tissue samples in an unsupervised manner. The scNiche approach reproducibly identified 3 separate liver cellular niches, which we annotated as hepatic, ductular and fibrotic based on their distinct cellular composition and proteomic profile.

As shown in Figure 3C, we did not have sufficient markers in our panel to separately annotate periportal hepatocytes. The cellular annotation is taken forward into the niche identification in scNiche, so we don't believe the location of periportal hepatocytes will affect our findings, as these have not been distinctly annotated. In the future, with larger panels and the inclusion of single-cell resolution spatial transcriptomics (e.g. Xenium or CosMx), it may be possible to further refine niche identification based on more granular annotation of hepatocyte subtypes.

In Fig. 3I, the presence of double-positive cells at the protein level is evident and should be acknowledged in the text. From a visualisation perspective, using red instead of orange for GPNMB would improve clarity.
Thanks. We have updated the text to explicitly state that there were some double-positive cells, although the majority of cells identified did not co-express both markers. We have used orange rather than red for colour blindness compatibility. We can of course change this colour scheme if the editors and reviewer feel this is necessary.

4. Fig ED 7F. Are the statistics missing here or is there no sig difference?
Apologies for the oversight. We have added the statistics to Extended Data Figures 7F and G.

5. Finally, in the multi-organoid culture system, the use of relatively high concentrations of dexamethasone should be discussed as a limitation. While beneficial for hepatocyte survival, dexamethasone is known to influence macrophage polarisation and may affect the interpretation of the results.
Thanks. We have added this to the discussion section.

Reviewer #2 (Remarks to the Author):

The Authors have comprehensively and appropriately addressed the Reviewer's comments.

Thanks to the reviewer for their input which helped us strengthen our manuscript during the first revision.

Reviewer #2 (Remarks on code availability):

This Reviewer does not have the expertise to review the code.

Reviewer #3 (Remarks to the Author):

I am generally happy with the revised manuscript, it reports an interesting observation and will stimulate further research in the field.However, some of my major comments were not really addressed (e.g. deciphering the true function of the SAMac subsets).
Thanks to the reviewer for their suggestions which helped us improve this study. We concur that this work will hopefully stimulate further research into this field, and ultimately identify more targeted ways of modulating pathogenic macrophages in CLD.

We agree with the reviewer that more work is needed to decipher the functional differences between SAMac1 and SAMac2 and have now explicitly discussed this in the manuscript.

I am also puzzled by the fact that the authors added scRNA-seq data from the BDL mouse model, but not from MASH-fibrosis models. This makes it more difficult to link the data to the human liver atlas, in which different stages of MASLD/MASH are represented, but neither cholestatic nor toxic injury (as modelled by CCl_4 or BDL).

In our initial assessment of SAMac abundance data from our human scRNAseq atlas, we saw an increase in OLR1+ SAMac2 across all CLD aetiologies (including biliary diseases PSC and PBC, alcohol-related liver disease and MASH), whilst the GPNMB+ SAMac1 population was more expanded in the biliary diseases PSC and PBC (Ext Data Fig 4E). OLR1 upregulation and SAMac2 expansion were also observed with increasing fibrosis stage in toxin (alcohol)-related liver disease and cholestatic diseases when analysing biopsy data from independent cohorts (Ext Data Fig 7K-P). Hence, our human data indicates that SAMac expansion occurs in toxic and biliary injury as well in MASH. Given this strong association between human biliary pathology and OLR1+ SAMac accumulation, we therefore wanted to add in a biliary mouse model for additional scRNAseq validation. Our multiplex staining does indicate similar SAMac heterogeneity in mouse MASH, with accumulation of both OLR1+ and GPNMB+ SAMacs (Ext Data Fig 8I), but future analyses of scRNAseq and spatial transcriptomic data from additional mouse MASH models will also be informative as to whether there are aetiology-specific differences in SAMac phenotype.

I understand that the in-vivo targeting of SAMac populations or their key distinguishing molecular marker was beyond the scope of the paper (or the capacity of the team). Hence, I support publication.

Thank you. We have also added the in vivo targeting of different SAMac subpopulations as a point for future work in the discussion section, as we agree that this is important.

Typo: Resmiterom (should be resmetirom).
Thanks for identifying this error. This has been corrected.
